# Exploring the Biodiversity and Antibacterial Potential of the Culturable Soil Fungi in Nyingchi, Tibet

**DOI:** 10.3390/jof11040276

**Published:** 2025-04-01

**Authors:** Shan-Shan Huang, Haishan Liu, Xia-Fei Li, Chun-Ying Wang, Xiujun Zhang, Juan-Juan Wang, Fuhang Song, Jie Bao, Hua Zhang

**Affiliations:** 1 School of Biological Science and Technology, University of Jinan, 336 West Road of Nan Xinzhuang, Jinan 250022, China; huangshanshanyq123@163.com (S.-S.H.); bio_liuhs@ujn.edu.cn (H.L.); 15350370512@163.com (X.-F.L.); w2518885679@163.com (C.-Y.W.); bio_zhangxj@ujn.edu.cn (X.Z.); bio_wangjj@ujn.edu.cn (J.-J.W.); 2 Key Laboratory of Geriatric Nutrition and Health, Ministry of Education of China, School of Light Industry, Beijing Technology and Business University, Beijing 100048, China; songfuhang@btbu.edu.cn

**Keywords:** Qinghai-Tibet Plateau, soil fungi, biodiversity, antibacterial activity, cultivable fungi

## Abstract

The Qinghai-Tibet Plateau, known as “the third pole” of the Earth, boasts unique climatic conditions with abundant sunlight, low temperature, and significant annual temperature variations, nurturing a diverse array of soil microorganisms with rich metabolic products. In this study, 132 fungal isolates were isolated and identified from the soil samples collected in Nyingchi, Tibet, belonging to 32 genera and 59 species, while Ascomycota, Mucoromycota, and Basidiomycota accounted for 91.7%, 7.5%, and 0.8%, respectively. The evolutionary relationships among 59 representative strains were revealed by constructing a phylogenetic tree, while *Penicillium*, *Fusarium,* and *Aspergillus* were the most widespread fungal genera here, and the antibacterial activity of these strains was evaluated by the agar diffusion assay, leading to 27 active strains. Notably, six of them demonstrated significant activities against two or more tested bacteria. The antibacterial efficacy of the extracts of these six fungi, which were derived from four distinct media, was further evaluated at a concentration of 500 μg/mL. This study provides a valuable supplement to the investigations of cultivable soil fungi in Nyingchi, Tibet, laying a foundation for the development of soil fungi and antibacterial lead compounds in the area.

## 1. Introduction

From the discovery of penicillin in 1928 to the golden age of antibiotic research during the 1940s–1970s, more than 20 types of antibiotics were discovered from bacteria or fungi, mainly including aminoglycosides, tetracyclines, β-lactams, and macrolides, which played an important role in human health [1]. However, with the widespread and excessive use of antibiotics, bacteria have developed various drug resistance mechanisms, such as target modification or mutation, secreting antibiotics hydrolase, an efflux pump, or initiation of self-repair systems, while rapidly evolving bacterial resistance has become one of the greatest threats to public health [2]. In 2017, the World Health Organization (WHO) published the first-ever list of antibiotic-resistant “priority pathogens”, covering 12 bacterial families that posed the most harmful factors to human health [3]. This list was updated in 2024 to 15 types with four key priority pathogens, seven high-priority pathogens, and four medium-priority pathogens [4]. The WHO Financial Model suggested that the average period for a new antimicrobial agent from discovery and preclinical to registration took about 10.1 years, and an antimicrobial product in the preclinical stage stood a chance of around 12.5% to successfully get through the registration phase [5]. From July 2017 to November 2021, only 12 new antibacterial drugs received approval from either the US Food and Drug Administration (FDA), the European Medicines Agency (EMA), or both [6]. Thus, the discovery of natural antibacterial products with low toxicity, high safety, and novel mechanisms of action is extremely significant for new antibiotics and public health security.

Soil is a vital carrier for a multitude of natural and biological resources, with fungi being one of its important constituents. As a significant repository of active lead compounds, soil fungi metabolites possess excellent biological activities. Zhang et al. [7] summarized the new compounds derived from soil fungi from 2011 to 2022; a total of 546 new metabolites were involved, mainly including alkaloids, terpenes, steroids, ketones, phenylpropanoids, and quinones. Of these, 51% of compounds exhibited good biological activities such as anticancer, antimicrobial, immunosuppressive, anti-inflammatory, and antioxidant, while molecules with antibacterial activities accounted for 30.8%, revealing great potential in the development of antibacterial lead compounds.

The region of Nyingchi, located on the Qinghai-Tibet Plateau known as “the third pole” of the Earth, boasts unique natural conditions, combining rich climatic diversity and distinctive geological features. This area is a typical high-altitude canyon region with an altitude variation in up to 7627 m, and the average annual rainfall ranges from 400 to 2200 mm [8]. The various vertical soil profiles, including lateritic red soil, yellow soil, brown soil, and chestnut soil under the forest vegetation, support the development of unique soil fungal communities that possess significant scientific value and considerable potential for further exploration [8,9]. Although earlier research has been conducted on the fungal diversity of the Qinghai-Tibet Plateau, such as the investigations on the fungal diversity of the root soil of *Oxytropis glacialis* [10], the soil fungi richness in the central Qinghai-Tibet Plateau [11], and the diversity of soil fungi in the Qaidam Basin and subalpine forests in Tibet [12,13], this research mainly focused on the fungal diversity by high-throughput sequencing. Research on the biodiversity and bioactivity of soil cultivable fungi in Tibet remains limited. In order to evaluate the fungal diversity of the soil in Nyingchi, Tibet, and their antibacterial effects, six soil samples from this region were collected and investigated, providing a reference for the development of fungal resources and antibacterial lead compounds.

## 2. Materials and Methods

### 2.1. Sampling Sites

**Figure 1:**
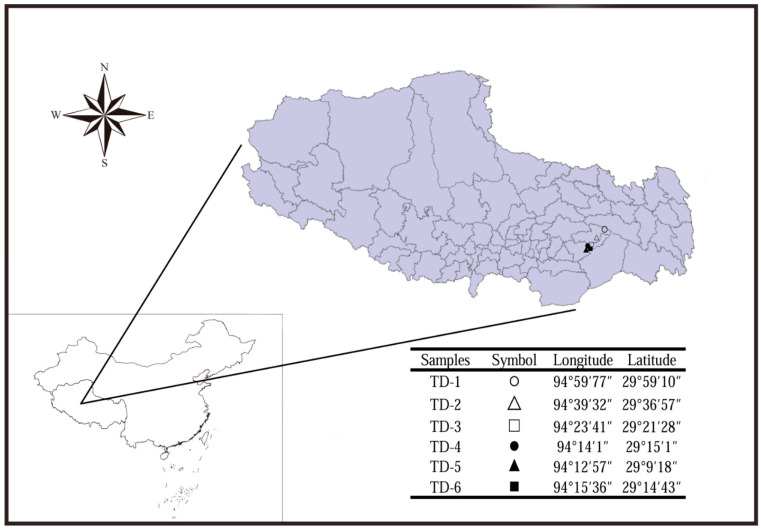
The map related to the sampling sites.

The six soil samples (TD-1~TD-6) were collected on 25 August 2023 from the Nyingchi area of Tibet (Figure 1 and Table 1). Each sample was gathered from two adjacent places and about 300 g for each sample.

### 2.2. Isolation and Identification of Fungi

The culturable fungi in the soil samples were isolated by diluted methods. The soil samples were diluted with sterile water to 10^−2^ and 10^−3^, and 100 μL of each suspension was plated onto different culture media, including yeast extract peptone dextrose medium (YPD), potato dextrose agar (PDA), malt extract agar (MEA), corn syrup agar (CSA), soluble starch agar (SSA), rose bengal agar (RBA), and Czapek dox agar (CDA) (Table 2), with 20 μg/mL kanamycin to suppress bacterial growth. Three replicates of each type of culture medium were used. Subsequently, the plates were incubated at 28 °C for three weeks, while the colonies were monitored daily and transferred to fresh PDA plates using sterile bamboo sticks. The purified fungal isolates were stored in glycerol suspensions (20%, *v*/*v*) and identified by morphological characteristics (morphology of colony and hyphal and sporulation characteristics) combined with internal transcribed spacer (ITS) sequences. The fungal culture was applied for DNA extraction, and primers ITS1 (5′-TCCGTAGGTGAACCTGCGC-3′) and ITS4 (5′-TCCTCCGCTTATTGATGC-3′) were employed to amplify the fungal internal transcribed spacer (ITS) region through polymerase chain reaction (PCR). The PCR amplification was performed using a 25 uL reaction system, including 12.5 µL of Taq premix, 1 µL template DNA, 1 µL (10 µM) of each primer, 1 µL DMSO, and 8.5 µL ddH_2_O (94 °C 2 min; 94 °C 20 s, 56 °C 20 s, 72 °C 30 s, 30 cycles; 72 °C 10 min), and the amplified products were sequenced by Qingdao Weilai Biotechnology Co., Ltd. (Qingdao, China). Finally, the results were compared with the sequences in the GenBank by Basic Local Alignment Search Tool (BLAST) programs (https://blast.ncbi.nlm.nih.gov/Blast.cgi?PROGRAM=blastn&PAGE_TYPE=BlastSearch&LINK_LOC=blasthome, accessed on 25 March 2025) to assess the sequence homology with closely related organisms.

### 2.3. Phylogenetic Analysis

Phylogenetic analysis independently for each fungal genus belonging to 59 different fungal taxa was conducted using Muscle in MEGA 12.0.9 based maximum likelihood (ML) [14,15], and provided in Supporting Information. The rDNA-ITS sequences of the target strains were entered into the Blastn search box to query the ITS sequences from the fungi type database in GenBank; the top 10 to 30 sequences with high coverage and similarity were selected based on the search results and imported into MEGA 12.0.9; the Muscle tool was used for multiple sequence alignment, while the sequences before the 3′ end marker (*) and after the 5′ end marker (*) were removed; the aligned sequences were then exported as a MEG file and re-imported into MEGA 12.0.9 to find optimal evolutionary models. Finally, a phylogenetic tree was constructed using the Maximum Likelihood (ML) with the following settings: standard bootstrap based on 1000 bootstrap replicates, the optimal evolutionary model (Supporting Information Table S1), complete deletion in Gaps/Missing Data, and the number of threads as eight. The ITS sequences of these 59 isolates were submitted to GenBank (accession numbers PQ152240–PQ152298).

### 2.4. Screening of Antibacterial Fungi by Agar Diffusion Assay

The modified agar diffusion assay [16] was applied to assess the antibacterial properties of the 59 representative fungal strains conveniently. The fungal isolates were initially cultured on PDA plates at 28 °C for 7 days. Subsequently, 6 mm plugs in diameter from tested fungi were prepared and placed on the agar media inoculated with pathogenic bacteria *Escherichia coli* (*E. coli* ATCC8739), *Vibrio parahaemolyticus* (*V. parahaemolyticus* ATCC17802), *Vibrio alginolyticus* (*V. alginolyticus* ATCC17749), and *Staphylococcus aureus* (*S. aureus* ATCC25923), respectively, for 12 h at 37 °C. The diameters of inhibition zones were recorded.

### 2.5. Fermentation and Extraction

On the basis of the agar diffusion assay, fungi with an inhibition zone greater than 10 mm against two or more pathogenic bacteria were selected for fermentation. To monitor the effects of the fermentation media on the fungal metabolites and their antibacterial activity, four growth media were adopted, and the compositions are listed in Table 3. The purified fungi were inoculated in 500 mL flasks containing 150 mL liquid medium or 350 g solid medium and cultured under static conditions at 28 °C for 30 days. The harvested culture was extracted with ethyl acetate (EtOAc) three times at a 1:1 volume ratio (culture: EtOAc), each time for 1 h. The organic layer was evaporated under reduced pressure (Heidolph Hei-VAPG3, Buchi, Switzerland) to obtain the crude extract, which was then dissolved in methanol, filtered with a 0.2 μm filter, and analyzed by HPLC on an Agilent 1260 instrument (Agilent Technologies Inc., Waldbronn, Germany) with YMC-Pack ODS-A columns (5 µm, 4.6 × 150 mm, YMC Co., Ltd., Tokyo, Japan). The detailed analysis conditions and results were provided in Supporting Information.

### 2.6. Antibacterial Activity Tests by Filter Paper Method and 96-Well Plate Method

The filter paper method [17] was employed to evaluate the antibacterial activity of the obtained extracts using the four pathogenic bacteria as those in 2.4. Bacterial solutions were seeded in LB medium, and sterile 6 mm filter papers (each with 100 μg sample) were placed on the plates with pathogenic bacteria at 37 °C for 24 h. Each sample was carried out in triplicate, and the inhibition zone diameters were measured and averaged.

Following the above trial, the inhibition rate (500 μg/mL) of the samples with ≥8 mm zones in the filter paper method was tested by the 96-well plate method [18] with norfloxacin and enrofloxacin as positive controls at 200 μg/mL.

## 3. Results and Analysis

### 3.1. Soil Fungal Diversity in Nyingchi, Tibet

From six soil samples collected in Nyingchi, Tibet, 132 fungal strains were isolated (Table 4). According to the rDNA-ITS sequences, which were submitted to NCBI for blast analysis, combined with morphological analysis, the 132 fungal strains belonged to 32 genera and 59 species, while Ascomycota, Mucoromycota, and Basidiomycota accounted for 91.7%, 7.5%, and 0.8%, respectively. In Ascomycota, Eurotiomycetes and Sordariomycetes were the dominant classes, representing 51.5% and 27.3% of the total strains, respectively, while the Aspergillaceae made up 46.7% of the strains in Eurotiomycetes and Nectriaceae comprised 11.4% of the strains in Sordariomycetes. Moreover, *Penicillium* is the most widespread genus, constituting 43.9% of all strains.

**Figure 2:**
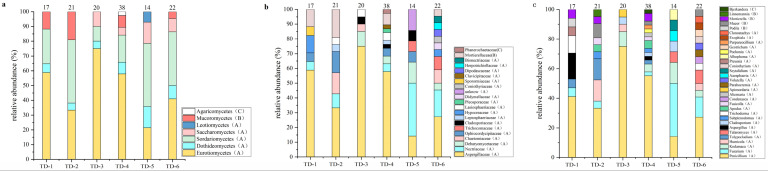
(**a**–**c**) Relative abundance of soil fungi at the level of class (**a**), family (**b**), and genus (**c**) in samples TD-1~TD-6 from Nyingchi, Tibet. (A), (B) and (C) represent Ascomycota, Mucoromycota and Basidiomycot respectively.

The analysis of the isolated soil fungi (Figure 2) revealed that the fungal composition of different soil samples had significant differences. For TD-1, Ascomycota and Mucoromycota accounted for 88.2% and 11.8%, respectively, in which three classes of Eurotiomycetes, Sordariomycetes, and Dothideomycetes were identified in Ascomycota, accounting for 58.8%, 23.5%, and 5.9%, and families of *Mortierella* (5.8%) and *Podila* (5.8%) were discovered in Mucoromycetes derived from Mucoromycota.

For TD-2, Ascomycota (80.9%) and Mucoromycota (11.8%) constituted the main fungal groups, while Eurotiomycetes, Sordariomycetes, and Dothideomycetes were identified in Ascomycota, accounting for 33.3%, 42.9%, and 4.8%, respectively, and only Mucoromycetes were discovered in Mucoromycota, including four genera: *Mucor* (9.5%), *Mortierella* (4.7%), *Linnemannia* (4.7%), and *Podila* (4.7%).

The fungal communities of TD-3 and TD-5 consisted entirely of Ascomycota, while four classes were identified as Eurotiomycetes (75.0%), Sordariomycetes (5.0%), Dothideomycetes (10.0%), and Saccharomycetes (10.0%) from TD-3, and five classes like Eurotiomycetes (21.4%), Sordariomycetes (42.9%), Dothideomycetes (14.3%), Saccharomycetes (14.3%), and Leotiomycetes (7.1%) were found in TD-5.

The fungal community of TD-4 was composed of three phyla as Ascomycota (89.5%), Mucoromycota (7.9%), and Basidiomycota (2.6%), while a total of four classes Eurotiomycetes (57.9%), Sordariomycetes (18.4%), Dothideomycetes (7.9%), and Saccharomycetes (5.3%) were identified in Ascomycota. Meanwhile, the Mucoromycota consisted of *Mortierella* (5.3%) and *Mucor* (2.6%), and only one *Bjerkandera* sp. fungus was found in Basidiomycota (2.6%).

Two phyla containing Ascomycota (95.5%) and Mucoromycota (4.5%) formed the fungal community of TD-6. Four classes of Eurotiomycetes, Sordariomycetes, Dothideomycetes, and Saccharomycetes, were identified in Ascomycota, accounting for 40.9%, 36.4%*,* 9.1%, and 9.1%, respectively, while Mucoromycetes consisted only of *Mucor* (4.5%).

Significant differences in the number and species distribution of fungi among the six samples could be observed in Figure 1. Specifically, the number of fungi isolated from TD-5 was the least, while that from TD-4 was the most. In addition, TD-4 had the most abundant fungal diversity, whereas TD-3 had the least. These data emphasized that the altitude and the associated physical and chemical characteristics of the soil, along with the climate conditions, were comprehensive, crucial environmental factors impacting the fungal community of soil [19]. The soil characteristics in different altitude areas, or the same altitude but with different environmental conditions, vary, which may affect the survival conditions, nutritional availability, and competitive interactions of fungi, forming their own unique fungal community.

### 3.2. Phylogenetic Analysis of the Isolated Culturable Fungi

**Figure 3:**
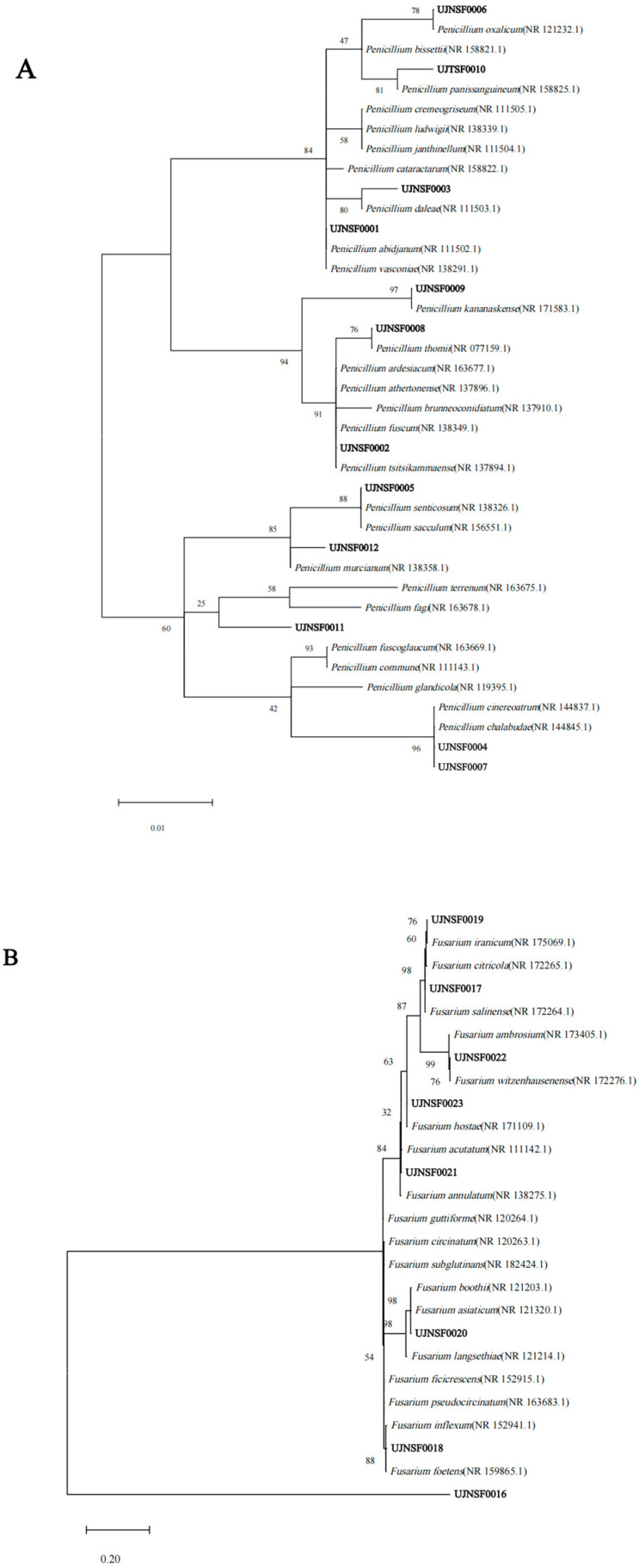
Maximum Likelihood phylogenetic trees of *Penicillium* (**A**) and *Fusarium* (**B**).

Based on the ITS-rDNA sequence homology of the 59 representative fungi from the 132 isolates, phylogenetic analysis independently for each fungal genus was conducted and provided in Supporting Information, while those for the two most abundant genera were shown in Figure 3. The 59 strains of fungi could be classified into seven classes, with Sordariomycetes and Eurotiomycetes being the predominant classes, containing 23 and 18 strains, respectively (Table 5). Else, Dothideomycetes, Mucoromycetes, Saccharomycetes, Leotiomycetes, and Agaricomycetes contained 8, 6, 2, 1, and 1 fungi, respectively. *Penicillium*, *Fusarium,* and *Aspergillus* were the most widespread fungal genera here, while *Penicillium* and *Aspergillus* were the most extensively investigated genera from soil for natural products [7]. It is worth noting that ten strains among these fungi had similarities < 97%, combined with three strains even with similarities < 90%, indicating the possibility of new species or genera, which required further investigation.

### 3.3. Screening of Antibacterial Fungi

Agar diffusion assay was applied to the 59 representative strains for antibacterial fungi against *E. coli* ATCC8739, *V. parahaemolyticus* ATCC17802, *V. alginolyticus* ATCC17749, and *S. aureus* ATCC25923, and 27 isolates exhibited varying effects against the four tested bacteria (Table 6). UJNSF0026 demonstrated the greatest potency against *Vibrio parahaemolyticus* and *Staphylococcus aureus*, with inhibition zone diameters of 14 mm and 27 mm, respectively, and UJNSF0039 showed the best activity against *Vibrio alginolyticus* with an inhibition zone diameter of 22 mm. In addition, only UJNSF0014 exhibited significant inhibitory activity against *Escherichia coli*. These fungi isolated from the Nyingchi region of Tibet possess excellent potential, revealing better effects against Gram-positive bacteria than Gram-negative ones.

### 3.4. The Antibacterial Activities of Crude Extracts of Fungi

A total of six fungal strains, which exhibited inhibition zone diameters greater than 10 mm against two or more pathogenic bacteria, were cultured under four distinct culture conditions to obtain 24 crude extracts. The antibacterial activity test showed that the crude extracts of the same fungi cultured under different conditions had obvious differences in activities (Table 7) and metabolites (Supporting Information), indicating that the culture conditions were crucial for fungal metabolites and their biological activities.

Subsequently, the antibacterial effects of the crude extracts, which had inhibition zone diameters greater than 8 mm in Table 7, were further evaluated by the inhibition rate with the 96-well plate method. As shown in Table 8, *Fusarium salinense* UJNSF0017 cultured in 2# medium had the best effect against *E. coli* with an inhibition rate of 69.75%, and *Talaromyces atricola* UJNSF0035 cultured in 2# medium exhibited the best effect against *V. parahaemolyticus* with an inhibition rate of 71.56%. Else, *Aspergillus terreus* UJNSF0014 incubated in 3# medium was observed to possess the most effective properties against *Vibrio alginolyticus* with an inhibition rate of 96.14%, and *Trichoderma longipile* UJNSF0039 incubated in 2# medium with an inhibition rate of 99.85% against *S. aureus*. These additional results further confirmed the antibacterial capabilities of the fungi under different medium conditions. Moreover, the antibacterial effects of these soil fungal metabolites with the 2# medium seem better, presenting that the necessary nutrients for the antibacterial metabolites by these fungi may be related to the 2# (oat) medium.

## 4. Discussion

Soil fungi play an important role in the operation of ecosystems, promoting the energy flow and material cycle of terrestrial ecosystems together with other microorganisms, and exhibit varying species diversity depending on the spatial environment and climatic conditions [20,21]. Soil fungal communities show various trends with altitude and soil depth. Generally, the species richness and quantity of soil fungi decrease with the increase in altitude. For example, Yao et al. [22] conducted research on the forest soil fungi in the Changbai Mountain and observed that as altitude increased, the diversity and quantity of soil fungi significantly decreased from coniferous-broadleaved forest to alpine tundra. As decomposers, soil fungi play a primary role in the conversion of nutrients and chemical components [23,24]. Additionally, soil fungi in specific habitats, to adapt to their living environment, have evolved a variety of biochemical and physiological functions necessary for survival [25], enabling them to suppress the growth of a variety of microorganisms and hold promise for the discovery of effective antimicrobial substances [26]. For example, Wang and Ning et al. [27,28] obtained a series of terpenoid compounds from the Arctic soil fungus *Eutypella* sp. D-1, some of which showed antibacterial activities against *Escherichia coli*, *Bacillus subtilis,* or *Vibrio vulnificus*. Xu et al. [29] obtained nine compounds from the *Aspergillus terreus* derived from Sichuan soil, including five alkaloids and four polyketides, while all of them displayed effective inhibitory effects on Gram-positive *Bacillus cereus*.

As the “third pole of the earth”, the Qinghai-Tibet Plateau has unique diversity and richness of soil fungi along with their metabolites, making them of high research value [30]. The Nyingchi region is one of the areas in the Qinghai-Tibet Plateau with the best hydrothermal conditions and unique microbial resources. Thus, we performed a study on the fungal diversity and antibacterial activity of soil samples from Nyingchi, Tibet, with TD-1~TD-2 and TD-3~TD-6 corresponding to plateau and climate, respectively. Through morphological and ITS nucleotide sequence analysis, a total of 132 fungal strains were isolated and identified, belonging to Ascomycota, Mucoromycota, and Basidiomycota. In Ascomycota, 5 classes, 18 families, and 27 genera were identified, one of which was not affiliated to the family. In Mucor, 1 class, 1 family, and 4 genera were identified, while 1 class, 1 family, and 1 genus were identified in Eurotium. The genetic and evolutionary connections of the 59 representative fungi isolates were analyzed using a phylogenetic tree. The geographical and climatic differences derived from soil samples were significant, leading to considerable variation in fungal diversity.

In light of the rich fungal resources in Nyingchi, Tibet, an agar diffusion assay, which is relatively fast and convenient, was used to evaluate the antibacterial activities of the isolated 132 strains of fungi. Among them, 6 fungal strains exhibited activities against two or more pathogenic bacteria with zone diameters greater than 10 mm. Then, four distinctive culture media were employed for them, and 24 fermentation crude extracts were obtained. Subsequently, the filter paper method and 96-well plate method were applied to determine the antibacterial activity of the crude extracts against *E. coli* ATCC8739, *V. parahaemolyticus* ATCC17802, *V. alginolyticus* ATCC17749, and *S. aureus* ATCC25923. The crude extracts of UJNSF0017, UJNSF0035, UJNSF0014, and UJNSF0039 demonstrated the best antibacterial effect against these four pathogens, respectively, which not only verified the specific antibacterial ability of these strains but also highlighted their great potential in the development of antibacterial agents.

Notable, according to the HPLC analysis of these crude extracts (Supporting Information), there were marked differences in the abundance and types of the metabolites, combined with the antibacterial activities of metabolites produced by the same strain under varying culture conditions. Furthermore, the influence of a single culture condition on the metabolites and activities of different fungi varies, and no single culture medium is optimal for all fungi. Additionally, although we currently cannot predict the specific types of metabolites produced by these fungi (this could be explored later using GNPS (Global Natural Products Social Molecular Networking) or other tools based on MS/MS [31,32]), we are able to investigate the relationship between the types of metabolites and their activities roughly. For instance, in UJNSF0014 and UJNSF0039, the metabolites detected in the 20–30 min seem to contribute more to the antibacterial effect than those in the 38–43 min. These also highlighted the significance of the OSMAC (One Strain Many Compounds) strategy in the investigation of fungal metabolites and their biological activities [33].

Agar diffusion assay, filter paper method, and 96-well plate method are the most common methods for evaluating antibacterial activity. In our work, the antibacterial activities of the crude extracts by the filter paper method (Table 7) and 96-well plate method (Table 8) were basically consistent, verifying the accuracy and reliability of the methods. However, there were some differences compared with the results from the agar diffusion assay, such as the antibacterial activities of UJNSF0017, which mainly exhibited the effects against *V. alginolyticus* and *S. aureus* in the agar diffusion assay but presented potential against *E. coli* in the filter paper or 96-well plate method. This might be attributed to the uneven inoculation amount and the various growth rates of the strains, especially in the agar diffusion assay, which could affect the accumulation and release of their metabolites, consequently influencing their bioactivities. This finding not only suggests the potential differences among different assessment methods but also emphasizes the importance of optimizing culture conditions for accurately evaluating the potential activity.

The current study not only expanded the understanding of the soil fungal diversity in Nyingchi, Tibet, but also offered meaningful evidence for its use as a repository of natural antibacterial active substances. Next, we will carry out the fermentation of the active fungi, isolation and identification of antibacterial ingredients, and exploration on the antibacterial mechanisms of the active metabolites, providing new ideas for the antibacterial agents.

## 5. Conclusions

In this study, 132 strains of fungi with high diversity and richness were isolated and identified from the soil of Nyingchi, Tibet, showing good potential for the development of antibacterial agents. This fills the gap in the research on fungal diversity and antibacterial activity in Nyingchi, Tibet, providing a basis for the development of compounds with unique activity. Together, it is helpful to better understand the complex ecosystem in the region and provide a scientific basis for environmental protection and sustainable development.

## Figures and Tables

**Table 1:** Details of the soil samples collected from Nyingchi, Tibet.

Samples	Altitude (m)	Features	Sample Environment	Soil Depth (cm)
TD-1	4089.2	Yellow sand granular	meadow	0–10
TD-2	4466.7	Black clumps	meadow	10–20
TD-3	2936.6	Brown sand blocks	bush	10–20
TD-4	2956.2	Black sand block	forest	10–20
TD-5	3138.9	Black mass	forest	20–30
TD-6	2913.4	Black clumps	forest	10–20

**Table 2:** Fungal isolation media.

Name	Formulas (L)
YPD	Yeast extract 5.0 g, peptone 10.0 g, glucose 20.0 g, agar 20.0 g
PDA	Potato 200.0 g, boiled to get filtrate, glucose 20.0 g, agar 20.0 g
MEA	Malt extract 20.0 g, peptone 1.0 g, agar 20.0 g
CSA	Corn syrup 7.0 g, agar 20.0 g
SSA	Soluble starch 10.0 g, peptone 1.0 g, glucose 10.0 g, K_2_HPO_4_ 1.0 g, MgSO_4_·7H_2_O 1.0 g, agar 20.0
RBA	Peptone 5.0 g, glucose 10.0 g, KH_2_PO_4_ 1.0 g, MgSO_4_·7H_2_O 0.5 g, rose bengal red 0.03 g, agar 20.0 g
CDA	NaNO_3_ 3.0 g, K_2_HPO_4_ 1.0 g, MgSO_4_·7H_2_O 0.5 g, KC1 0.5 g, FeSO_4_·7H_2_O 0.01 g, sucrose 30.0 g, agar 20.0 g

**Table 3:** Fungal growth media.

Names	Formulas
1# (solid)	Rice 80.0 g, yeast extract 0.1 g, dextrose 0.1 g, distilled water 120 mL
2# (solid)	Oat 80.0 g, yeast extract 0.1 g, dextrose 0.1 g, distilled water 120 mL
3# (liquid)	Glucose 10.0 g, mannitol 20.0 g, maltose 20.0 g, corn syrup 1.0 g, distilled water 1 L
4# (liquid)	Yeast extract 3.0 g, malt extract 3.0 g, peptone 1.0 g, glucose 2.0 g, distilled water 1 L

**Table 4:** Fungal diversity of six soil samples collected in Nyingchi, Tibet.

Fungal Family	Fungal Genus	Number of Isolates (Isolation Frequency (%))					
		TD-1	TD-2	TD-3	TD-4	TD-5	TD-6
Aspergillaceae	*Penicillium*	7 (41.2)	7 (33.3)	15 (75.0)	21 (55.3)	2 (14.3)	6 (27.3)
	*Aspergillus*	3 (17.6)	0 (0)	0 (0)	1 (2.6)	0 (0)	0 (0)
Nectriaceae	*Fusarium*	1 (5.9)	1 (4.8)	0 (0)	1 (2.6)	5 (35.7)	3 (13.6)
	*Fusicolla*	0 (0)	1 (4.8)	0 (0)	1 (2.6)	0 (0)	0 (0)
	*Volutella*	0 (0)	0 (0)	0 (0)	0 (0)	0 (0)	1 (4.5)
Debaryomycetaceae	*Kodamaea*	0 (0)	0 (0)	2 (10.0)	2 (5.3)	2 (14.3)	1 (4.5)
Chaetomiaceae	*Humicola*	0 (0)	3 (14.3)	1 (5.0)	0 (0)	0 (0)	1 (4.5)
	*Condenascu*	0 (0)	0 (0)	0 (0)	0 (0)	0 (0)	1 (4.5)
Ophiocordycipitaceae	*Tolypocladium*	1 (5.9)	3 (14.3)	0 (0)	1 (2.6)	0 (0)	0 (0)
	*Albophoma*	0 (0)	0 (0)	0 (0)	0 (0)	1 (7.1)	0 (0)
	*Purpureocillium*	0 (0)	0 (0)	0 (0)	1 (0)	0 (0)	0 (0)
Trichocomaceae	*Talaromyces*	0 (0)	0 (0)	0 (0)	0 (0)	1 (7.1)	2 (9.1)
Cladosporiaceae	*Cladosporium*	0 (0)	0 (0)	1 (5.0)	1 (2.6)	1 (7.1)	0 (0)
Leptosphaeriaceae	*Subplenodomus*	0 (0)	1 (4.8)	0 (0)	1 (2.6)	0 (0)	0 (0)
Hypocreaceae	*Trichoderma*	2 (11.8)	0 (0)	0 (0)	0 (0)	0 (0)	1 (4.5)
Lasiosphaeriaceae	*Apodus*	0 (0)	1 (4.8)	0 (0)	2 (5.2)	0 (0)	0 (0)
	*Apiosordaria*	0 (0)	0 (0)	1 (5.0)	0 (0)	0 (0)	0 (0)
Pleosporaceae	*Alternaria*	0 (0)	0 (0)	0 (0)	1 (2.6)	0 (0)	0 (0)
Didymellaceae	*Paraboeremia*	0 (0)	0 (0)	0 (0)	0 (0)	0 (0)	1 (4.5)
Coniothyriaceae	*Coniothyrium*	0 (0)	0 (0)	0 (0)	0 (0)	0 (0)	1 (4.5)
Sporormiaceae	*Preussia*	1 (5.9)	0 (0)	0 (0)	0 (0)	0 (0)	0 (0)
Clavicipitaceae	*Pochonia*	0 (0)	0 (0)	0 (0)	1 (2.6)	0 (0)	0 (0)
Dipodascaceae	*Geotrichum*	0 (0)	0 (0)	0 (0)	0 (0)	0 (0)	1 (4.5)
Herpotrichiellaceae	*Exophiala*	0 (0)	0 (0)	0 (0)	0 (0)	0 (0)	1 (4.5)
Bionectriaceae	*Clonostachys*	0 (0)	0 (0)	0 (0)	0 (0)	0 (0)	1 (4.5)
Mortierellaceae	*Linnemannia*	0 (0)	1 (4.8)	0 (0)	0 (0)	0 (0)	0 (0)
	*Podila*	1 (5.9)	0 (0)	0 (0)	0 (0)	0 (0)	0 (0)
	*Mucor*	0 (0)	2 (9.2)	0 (0)	1 (2.6)	0 (0)	1 (4.5)
	*Mortierella*	1 (5.9)	1 (4.8)	0 (0)	2 (5.2)	0 (0)	0 (0)
Phanerochaetaceae	*Bjerkandera*	0 (0)	0 (0)	0 (0)	1 (2.6)	0 (0)	0 (0)
Unknow	*Aaosphaeria*	0 (0)	0 (0)	0 (0)	0 (0)	1 (7.1)	0 (0)
	*Scytalidium*	0 (0)	0 (0)	0 (0)	0 (0)	1 (7.1)	0 (0)
Total		17	21	20	38	14	22

**Table 5:** Distribution of 59 representative fungal species in six samples from Nyingchi, Tibet.

Fungal Class	Fungal Genus	Fungal Taxa	Representative Isolate (Accession Number in GenBank)	Sequence in Genbank	Identities	Number of Fungal Isolates					
						TD-1	TD-2	TD-3	TD-4	TD-5	TD-6
*Eurotiomycetes*	*Penicillium*	*P. vasconiae*	UJNSF0001 (PQ152240)	NR 138291.1	100.00%	1	0	4	0	0	1
		*P. fuscum*	UJNSF0002 (PQ152241)	NR 138349.1	100.00%	2	0	1	2	0	0
		*P. daleae*	UJNSF0003 (PQ152242)	NR 111503.1	98.42%	1	2	0	2	1	1
		*P. chalabudae*	UJNSF0004 (PQ152243)	NR 144845.1	99.47%	1	0	10	14	0	0
		*P.* *sacculum*	UJNSF0005 (PQ152244)	NR 156551.1	99.80%	1	0	0	0	0	0
		*P.* *oxalicum*	UJNSF0006 (PQ152245)	NR 121232.1	100.00%	1	0	0	0	0	0
		*P. cinereoatrum*	UJNSF0007 (PQ152246)	NR 144837.1	99.60%	0	2	0	2	0	0
		*P.* *thomii*	UJNSF0008 (PQ152247)	NR 077159.1	99.47%	0	1	0	0	0	0
		*P kananaskense*	UJNSF0009 (PQ152248)	NR 171583.1	100.00%	0	2	0	0	0	0
		*P. panissanguineum*	UJTSF0010 (PQ152249)	NR 158825.1	98.14%	0	0	0	1	0	0
		*P. glandicola*	UJNSF0011 (PQ152250)	NR 119395.1	99.82%	0	0	0	0	1	0
		*P. murcianum*	UJNSF0012 (PQ152251)	NR 138358.1	99.15%	0	0	0	0	0	4
*Eurotiomycetes*	*Aspergillus*	*A. versicolor*	UJNSF0013 (PQ152252)	NR 131277.1	98.07%	2	0	0	0	0	0
		*A. terreus*	UJNSF0014 (PQ152253)	NR 131276.1	99.83%	1	0	0	0	0	0
		*A. flavus*	UJNSF0015 (PQ152254)	NR 111041.1	99.50%	0	0	0	1	0	0
*Sordariomycetes*	*Fusarium*	*F. pseudoanthophilum*	UJNSF0016 (PQ152255)	NR 163682.1	99.08%	1	0	0	0	0	0
		*F.salinense*	UJNSF0017 (PQ152256)	NR 172264.1	99.46%	0	1	0	0	0	0
		*F. foetens*	UJNSF0018 (PQ152257)	NR 159865.1	99.22%	0	0	0	1	2	0
		*F. iranicum*	UJNSF0019 (PQ152258)	NR 175069.1	99.46%	0	0	0	0	1	1
		*F. asiaticum*	UJNSF0020 (PQ152259)	NR 121320.1	99.27%	0	0	0	0	1	0
		*F. annulatum*	UJNSF0021 (PQ152260)	NR 138275.1	99.64%	0	0	0	0	1	0
		*F. witzenhausenense*	UJNSF0022 (PQ152261)	NR 172276.1	99.63%	0	0	0	0	0	1
		*F. hostae*	UJNSF0023 (PQ152262)	NR 171109.1	99.81%	0	0	0	0	0	1
*Sordariomycetess*	*Fusicolla*	*F. aquaeductuum*	UJNSF0024 (PQ152263)	NR 173406.1	99.11%	0	1	0	1	0	0
*Sordariomycetess*	*Volutella*	*V. salvadorae*	UJNSF0025 (PQ152264)	NR 173060.1	94.58%	0	0	0	0	0	1
*Saccharomycetes*	*Kodamaea*	*K. ohmeri*	UJNSF0026 (PQ152265)	NR 121464.1	94.88%	0	0	2	2	2	1
*Sordariomycetes*	*Humicola*	*H. nigrescens*	UJNSF0027 (PQ152266)	NR 077125.1	99.46%	0	3	1	0	0	1
*Sordariomycetes*	*Condenascu*	*C. tortuosus*	UJNSF0028 (PQ152267)	NR 160164.1	99.19%	0	0	0	0	0	1
*Sordariomycetes*	*Tolypocladium*	*T. album*	UJNSF0029 (PQ152268)	NR 155018.1	97.38%	1	0	0	0	0	0
		*T. inflatum*	UJNSF0030 (PQ152269)	NR 171737.1	100.0%	0	3	0	0	0	0
		*T. cylindrosporum*	UJNSF0031 (PQ152270)	NR 167967.1	99.82%	0	0	0	1	0	0
*Sordariomycetes*	*Albophoma*	*A. yamanashiensis*	UJNSF0032 (PQ152271)	NR 171222.1	99.65%	0	0	0	0	1	0
*Sordariomycetes*	*Purpureocillium*	*P. lilacinum*	UJNSF0033 (PQ152272)	NR 165946.1	100.00%	0	0	0	1	0	0
*Eurotiomycetes*	*Talaromyces*	*T. adpressus*	UJNSF0034 (PQ152273)	NR 171595.1	98.63%	0	0	0	0	1	0
		*T. atricola*	UJNSF0035 (PQ152274)	NR 147429.1	99.64%	0	0	0	0	0	2
*Dothideomycetes*	*Cladosporium*	*C.velox*	UJNSF0036 (PQ152275)	NR 119604.1	100.00%	0	0	1	0	0	0
		*C. oxysporum*	UJNSF0037 (PQ152276)	NR 119855.1	99.62%	0	0	0	1	1	0
*Dothideomycetes*	*Subplenodomus*	*S. galicola*	UJNSF0038 (PQ152277)	NR 154454.1	95.06%	0	1	0	1	0	0
*Sordariomycetes*	*Trichoderma*	*T. longipile*	UJNSF0039 (PQ152278)	NR 134354.1	99.83%	2	0	0	0	0	0
		*T. vinosum*	UJNSF0040 (PQ152279)	NR 144870.1	99.81%	0	0	0	0	0	1
*Sordariomycetes*	*Apodus*	*A. deciduus*	UJNSF0041 (PQ152280)	NR 145141.1	87.55%	0	1	0	2	0	0
*Sordariomycetes*	*Apiosordaria*	*A. microcarpa*	UJNSF0042 (PQ152281)	NR 175133.1	95.32%	0	0	1	0	0	0
*Dothideomycetes*	*Alternaria*	*A. doliconidium*	UJNSF0043 (PQ152282)	NR 137143.1	99.62%	0	0	0	1	0	0
*Dothideomycetes*	*Paraboeremia*	*P. camelliae*	UJNSF0044 (PQ152283)	NR 158251.1	99.38%	0	0	0	0	0	1
*Dothideomycetes*	*Coniothyrium*	*C. hakeae*	UJNSF0045 (PQ152284)	NR 154839.1	93.70%	0	0	0	0	0	1
*Dothideomycetes*	*Preussia*	*P. flanaganii*	UJNSF0046 (PQ152285)	NR 077168.1	97.74%	1	0	0	0	0	0
*Sordariomycetes*	*Pochonia*	*P. chlamydosporia var. chlamydosporia*	UJNSF0047 (PQ152286)	NR 171784.1	84.15%	0	0	0	1	0	0
*Saccharomycetes*	*Geotrichum*	*G. silvicola*	UJNSF0048 (PQ152287)	NR 077071.1	97.01%	0	0	0	0	0	1
*Eurotiomycetes*	*Exophiala*	*E. radicis*	UJNSF0049 (PQ152288)	NR 158397.1	100.00%	0	0	0	0	0	1
*Sordariomycetes*	*Clonostachys*	*C. aranearum*	UJNSF0050 (PQ152289)	NR 164542.1	99.28%	0	0	0	0	0	1
*Mucoromycetes*	*Linnemannia*	*L. zychae*	UJNSF0051 (PQ152290)	NR 111576.1	97.47%	0	1	0	0	0	0
*Mucoromycetes*	*Podila*	*P. horticola*	UJNSF0052 (PQ152291)	NR 111572.1	99.83%	1	0	0	0	0	0
*Mucoromycetes*	*Mucor*	*M. hiemalis f. hiemalis*	UJNSF0053 (PQ152292)	NR 152948.1	99.20%	0	2	0	1	0	0
		*M. moelleri*	UJNSF0054 (PQ152293)	NR 111659.1	100.00%	0	0	0	0	0	1
*Mucoromycetes*	*Mortierella*	*M. rishikesha*	UJNSF0055 (PQ152294)	NR 111564.1	98.16%	1	1	0	1	0	0
		*M. globalpina*	UJNSF0056 (PQ152295)	NR 160121.1	94.99%	0	0	0	1	0	0
*Agaricomycetes*	*Bjerkandera*	*B. ecuadorensis*	UJNSF0057 (PQ152296)	NR 174062.1	85.63%	0	0	0	1	0	0
*Dothideomycetes*	*Aaosphaeria*	*A. arxii*	UJNSF0058 (PQ152297)	NR 169685.1	99.65%	0	0	0	0	1	0
*Leotiomycetes*	*Scytalidium*	*S. circinatum*	UJNSF0059 (PQ152298)	NR 160180.1	94.07%	0	0	0	0	1	0
		Total				17	21	20	38	14	22

**Table 6:** The 27 fungi with antibacterial effects.

Strain Number	Pathogenic Bacteria (Inhibition Zone Diameter/mm)			
	*E*. *coli* (G^−^)	*V*. *parahaemolyticus* (G^−^)	*V*. *alginolyticus* (G^−^)	*S*. *aureus* (G^+^)
UJNSF0001	-	-	15.0 ± 0.1	
UJNSF0002	-	-	14.5 ± 0.5	
UJNSF0003	-	9.0 ± 0.2	15.0 ± 0.2	11.0 ± 0.4
UJNSF0004	-	-	13.0 ± 0.2	
UJNSF0006	-	-	-	11.0 ± 0.1
UJNSF0008	-	-	10.0 ± 0.4	
UJTSF0010	-	-	-	25.0 ± 0.5
UJNSF0012	-	-	-	6.5 ± 0.5
UJNSF0013	-	-	-	10.0 ± 0.5
UJNSF0014	11.0 ± 0.5	12.0 ± 0.5	20.0 ± 0.4	16.0 ± 0.4
UJNSF0017	-	-	16.0 ± 0.1	14.0 ± 0.5
UJNSF0018	-	-	-	11.0 ± 0.5
UJNSF0020	-	-	-	7.5 ± 0.5
UJNSF0021	-	-	-	25.0 ± 0.2
UJNSF0022	-	-	-	19.0 ± 0.4
UJNSF0024	-	-	-	8.0 ± 0.2
UJNSF0026	-	14.0 ± 0.3	-	27.0 ± 0.5
UJNSF0034	-	-	-	22.5 ± 0.4
UJNSF0035	-	-	12.0 ± 0.4	10.0 ± 0.4
UJNSF0038	-	-	-	19.0 ± 0.5
UJNSF0039	-	10.0 ± 0.2	22.0 ± 0.5	13.0 ± 0.5
UJNSF0040	-	-	-	21.0 ± 0.4
UJNSF0047	-	-	-	6.5 ± 0.1
UJNSF0050	-	-	-	9.0 ± 0.5
UJNSF0056	-	-	-	9.0 ± 0.2
UJNSF0057	-	-	-	19.0 ± 0.5
UJNSF0059	-	-	-	12.0 ± 0.2

Note: G^−^ indicates Gram-negative bacteria, G^+^ indicates Gram-positive bacteria, and “-” means the inhibition zone diameter < 6.5 mm.

**Table 7:** Antibacterial effects of the crude extracts from six fungi with different culture conditions.

Strain Number	Medium	Pathogenic Bacteria (Inhibition Zone Diameter/mm)			
		*E. coli* (G^−^)	*V. parahaemolyticus* (G^−^)	*V. alginolyticus* (G^−^)	*S. aureus* (G^+^)
UJNSF0003	1#	-	-	-	-
	2#	-	-	-	-
	3#	-	-	-	10.50 ± 0.22
	4#	-	7.83 ± 0.28	-	6.67 ± 0.17
UJNSF0014	1#	7.67 ± 0.12	7.67 ± 0.33	10.33 ± 0.27	15.33 ± 0.25
	2#	9.33 ± 0.10	8.67 ± 0.23	9.67 ± 0.10	13.33 ± 0.24
	3#	10.33 ± 0.35	9.33 ± 0.34	11.33 ± 0.15	7.33 ± 0.21
	4#	-	7.33 ± 0.37	-	7.33 ± 0.26
UJNSF0017	1#	7.33 ± 0.14	-	7.67 ± 0.37	12.67 ± 0.33
	2#	10.50 ± 0.33	-	-	15.33 ± 0.37
	3#	9.33 ± 0.23	-	9.33 ± 0.33	13.33 ± 0.23
	4#	7.67 ± 0.24	-	-	7.33 ± 0.32
UJNSF0026	1#	-	7.33 ± 0.32	-	7.67 ± 0.17
	2#		-	-	-
	3#		7.83 ± 0.28	-	9.33 ±0.42
	4#	-	8.33 ± 0.27	-	8.67 ± 0.23
UJNSF0035	1#	-	8.33 ± 0.33	-	9.67 ± 0.17
	2#	-	9.67 ± 0.25	7.33 ± 0.24	11.67 ± 0.27
	3#	-	8.67 ± 0.17	8.00 ± 0.10	8.33 ± 0.12
	4#	-	7.67 ± 0.22	7.67 ± 0.12	9.67 ± 0.13
UJNSF0039	1#	-	9.33 ± 0.27	11.33 ± 0.27	7.67 ± 0.14
	2#	7.50 ± 0.23	7.33 ± 0.11	-	15.00 ± 0.22
	3#	7.33 ± 0.21	7.67 ± 0.23	9.33 ± 0.23	10.67 ± 0.24
	4#	-	9.33 ± 0.13	-	7.67 ± 0.16

Note: G^−^ indicates Gram-negative bacteria, G^+^ indicates Gram-positive bacteria, and “-” indicates the inhibition zone diameter < 6.5 mm.

**Table 8:** Antibacterial effects of crude extracts by 96 well plate method (Inhibition rate).

Strain Number	Medium	*E*. *coli* (G^−^)	*V*. *parahaemolyticus* (G^−^)	*V*. *alginolyticus* (G^−^)	*S*. *aureus* (G^+^)
UJNSF0003	3#	-	-	-	90.35%
UJNSF0014	1#	-	-	80.30%	97.18%
	2#	-	-	83.18%	89.57%
	3#	60.18%	35.43%	96.14%	-
UJNSF0017	1#	-	-	84.31%	-
	2#	69.75%	-	-	97.49%
	3#	-	-	58.87%	96.76%
	4#	-	-	-	-
UJNSF0026	3#	-	-	-	96.76%
	4#	-	38.30%	-	87.23%
UJNSF0035	1#	-	-	-	96.84%
	2#	-	71.56%	88.11%	95.87%
	3#	-	-	-	-
	4#	-	-	53.38%	-
UJNSF0039	1#	-	35.29%	92.98%	-
	2#	-	-	-	99.85%
	3#	-	-	73.79%	93.32%
	4#	-	37.73%	-	-
enrofloxacin		100%	74.03%	100%	97.83%
norfloxacin		89.12%	100%	98.29%	99.86%

Note: - indicates the inhibition rate < 30%.

## Data Availability

The original contributions presented in this study are included in the article/Supplementary Materials. Further inquiries can be directed to the corresponding authors.

## Supplementary Materials

The following supporting information can be downloaded at: https://www.mdpi.com/article/10.3390/jof11040276/s1, the morphological characteristics of the 59 representative isolates; the phylogenetic analysis independently for 32 fungal genera; the HPLC analysis of the 24 crude extracts derived from the six strains with well antibacterial activities.
