# Supplementary material for: Exploring the Biodiversity and Antibacterial Potential of the Culturable Soil Fungi in Nyingchi, Tibet
Source: J Fungi (Basel). 2025 Apr 1;11(4):276. doi: 10.3390/jof11040276 (PMC12028717; doi:10.3390/jof11040276)

***Supporting information* for**

Exploring the Biodiversity and Antibacterial Potential of the Culturable Soil Fungi in Nyingchi, Tibet

Shan-Shan Huang [1], Haishan Liu [1], Xia-Fei Li [1], Chun-Ying Wang [1], Xiujun Zhang [1], Juan-Juan Wang [1], Fuhang Song [2], Jie Bao[1,]* and Hua Zhang[1,]*

[1] School of Biological Science and Technology, University of Jinan, 336 West Road of Nan Xinzhuang, Jinan 250022, China

[2] Key Laboratory of Geriatric Nutrition and Health, Ministry of Education of China, School of Light Industry, Beijing Technology and Business University, Beijing 100048, China

* Correspondences: bio_baoj@ujn.edu.cn (J.B.); bio_zhangh@ujn.edu.cn (H.Z.); Tel.: +86-0531-89736199 (H.Z.)

Table of contents

1.The morphological characteristics of the 59 representative isolates	P1
2. Phylogenetic analysis independently for 32 fungal genera	P6
3. The HPLC analysis of the 24 crude extracts derived from the six strains with well antibacterial activities	P39

1. The morphological characteristics of the 59 representative isolates

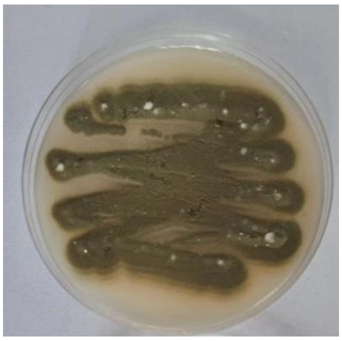
UJNSF0001

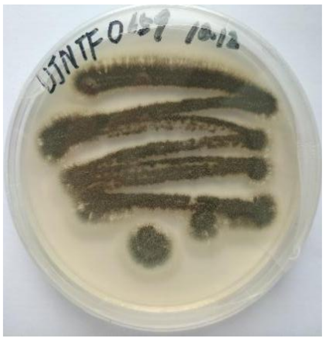
UJNSF0002

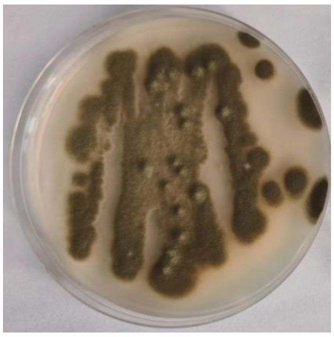
UJNSF0003

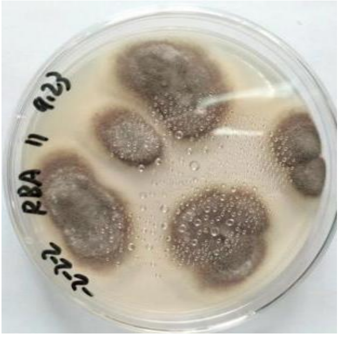

UJNSF0004

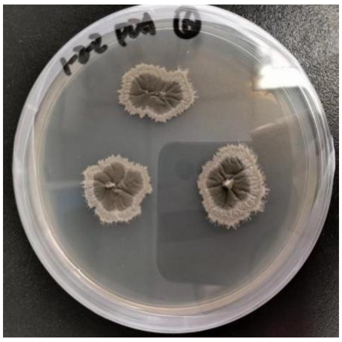
UJNSF0005

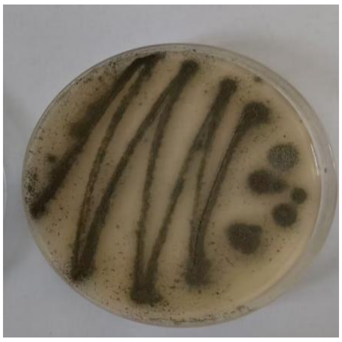
UJNSF0006

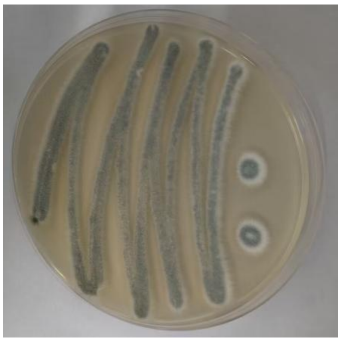
UJNSF0007

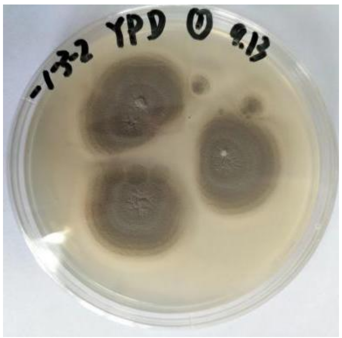

UJNSF0008

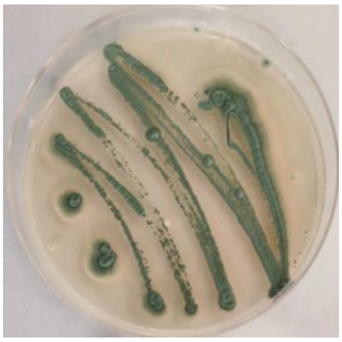
UJNSF0009

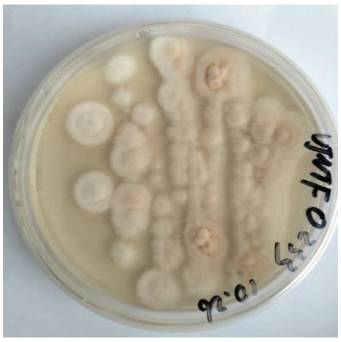
UJNSF0010

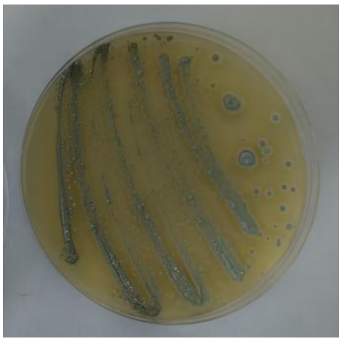
UJNSF0011

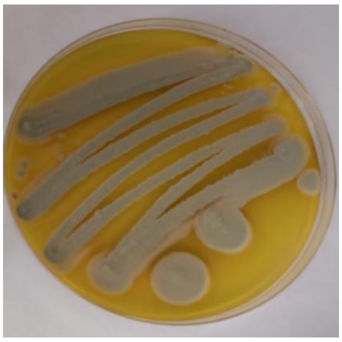
UJNSF0012

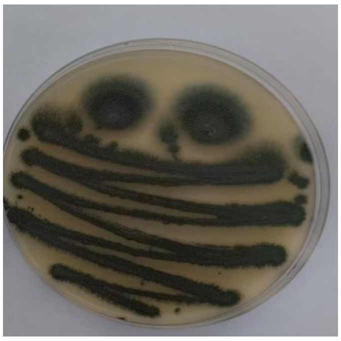
UJNSF0013

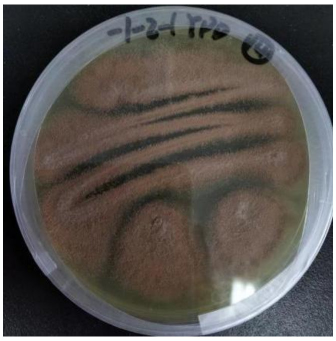
UJNSF0014

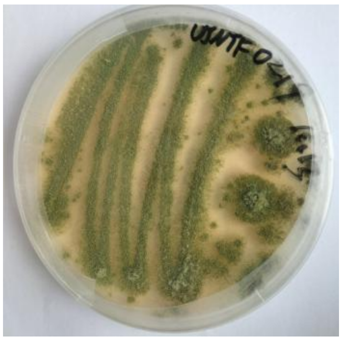
UJNSF0015

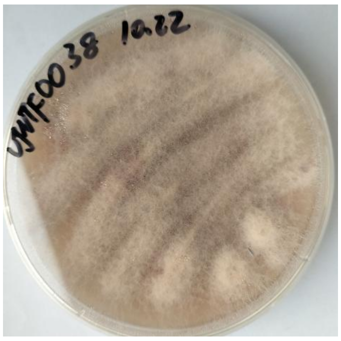

UJNSF0016

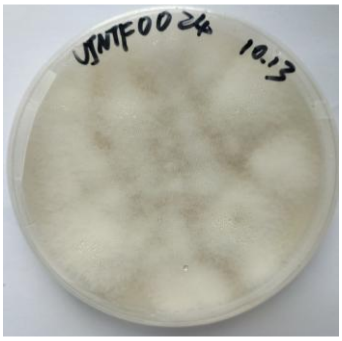

UJNSF0017

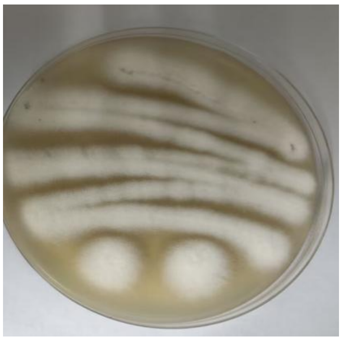
UJNSF0018

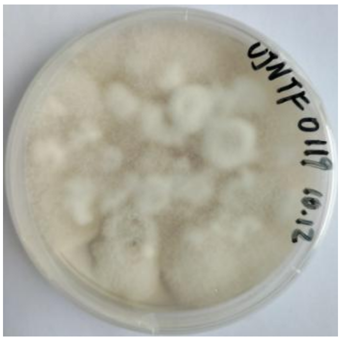
UJNSF0019

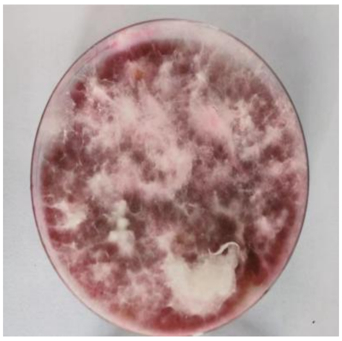
UJNSF0020

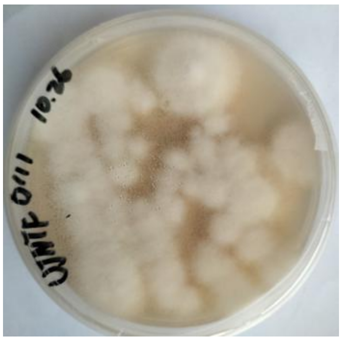

UJNSF0021

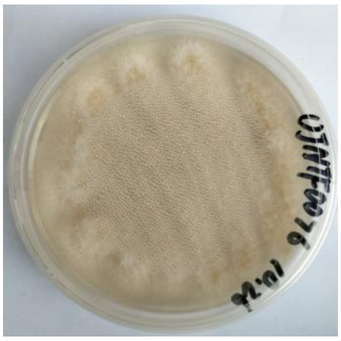
UJNSF0022

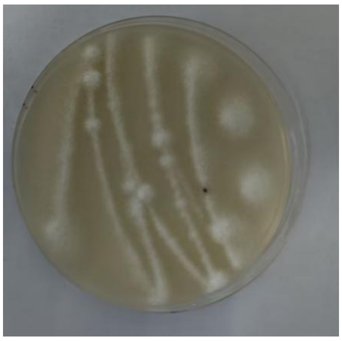
UJNSF0023

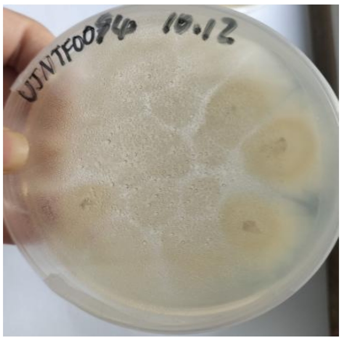
UJNSF0024

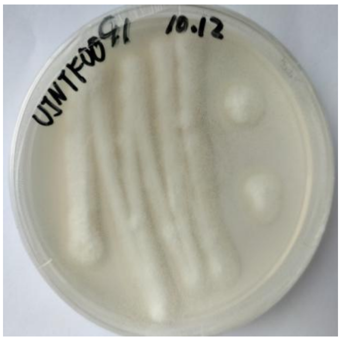

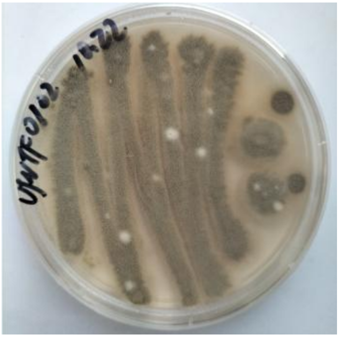

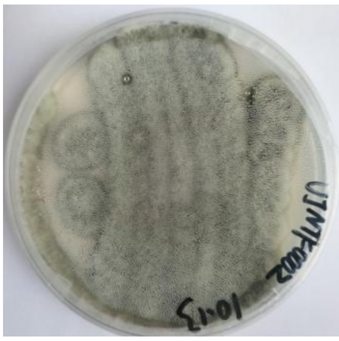

UJNSF0025 UJNSF0026 UJNSF0027

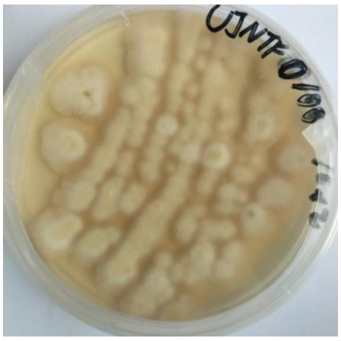 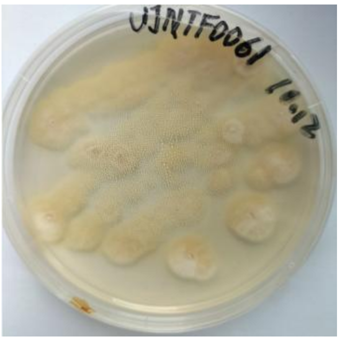 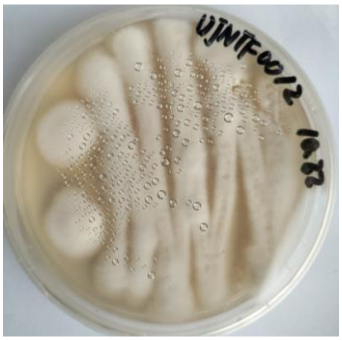

UJNSF0028 UJNSF0029 UJNSF0030

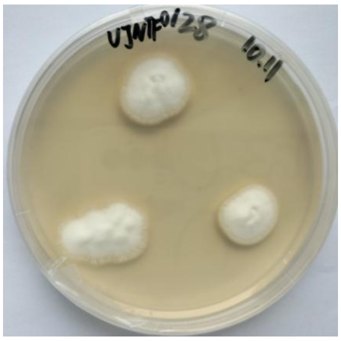 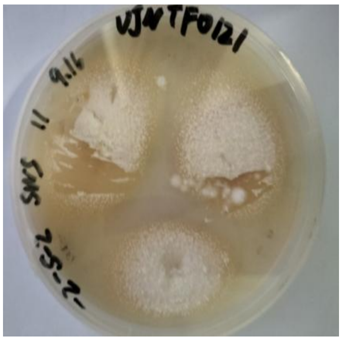 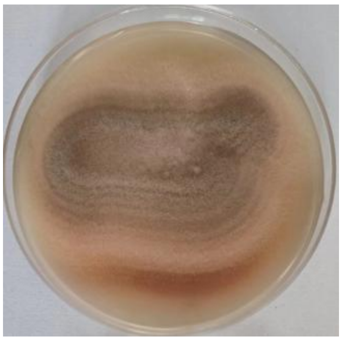

UJNSF0031 UJNSF0032 UJNSF0033

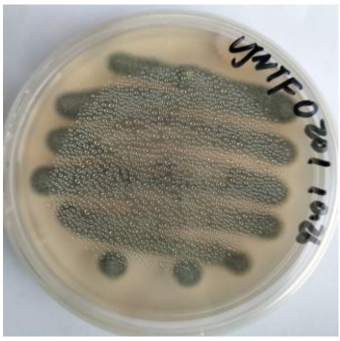 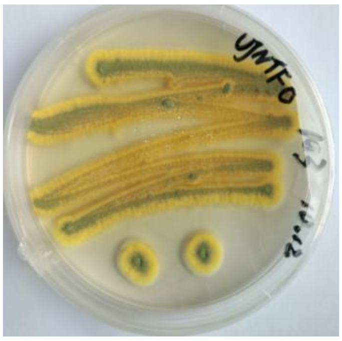 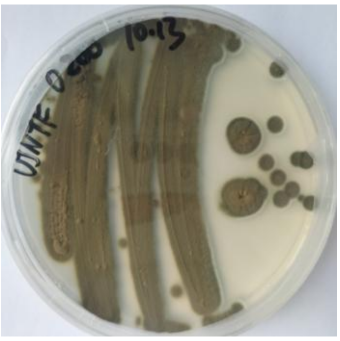

UJNSF0034 UJNSF0035 UJNSF0036

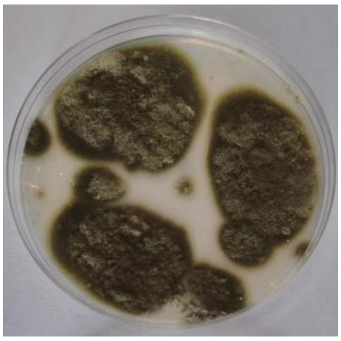 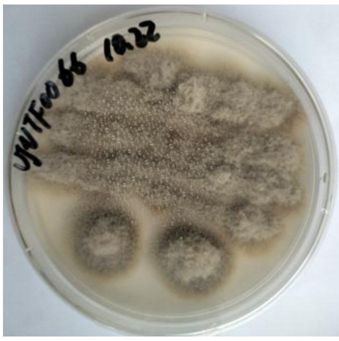 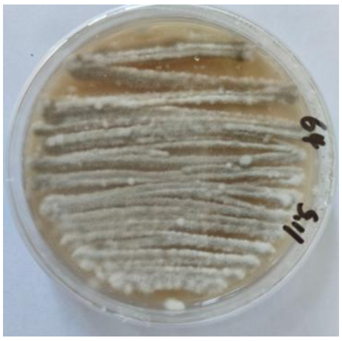

UJNSF0037 UJNSF0038 UJNSF0039

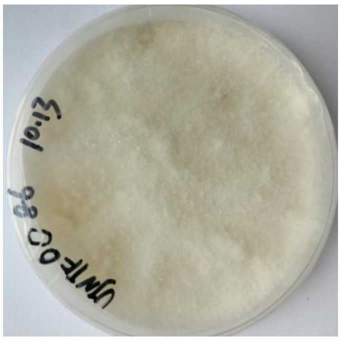 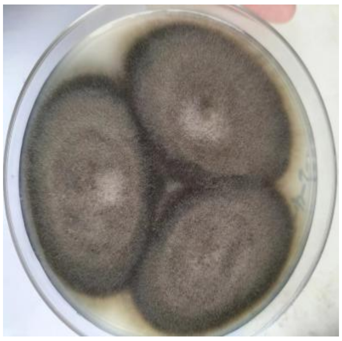 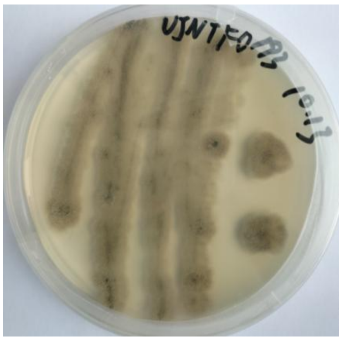

UJNSF0040 UJNSF0041 UJNSF0042

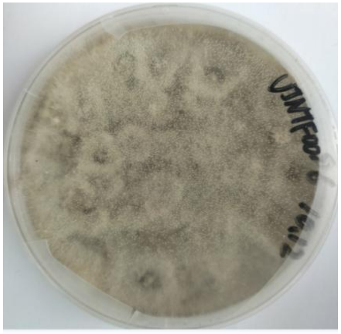
UJNSF0043

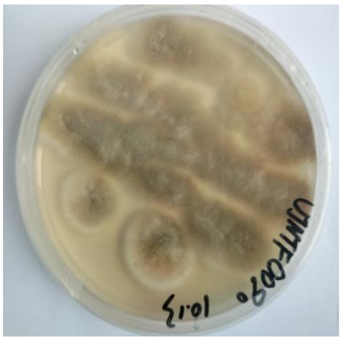
UJNSF0044

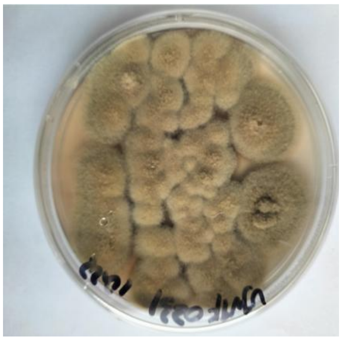
UJNSF0045

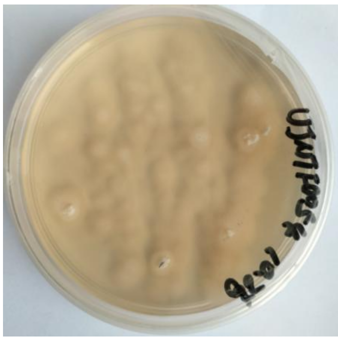
UJNSF0046

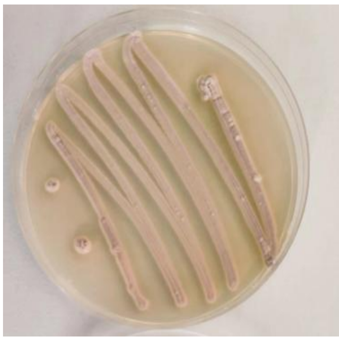
UJNSF0047

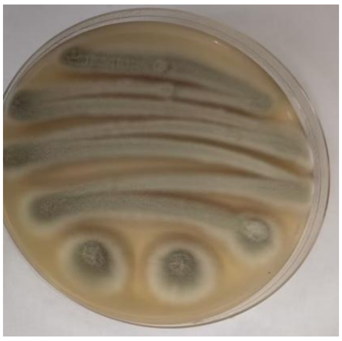
UJNSF0048

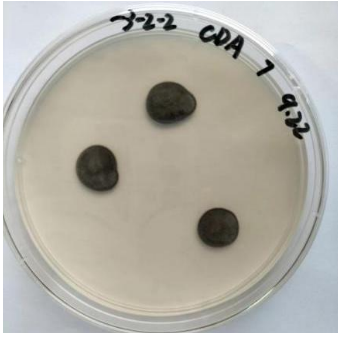

UJNSF0049

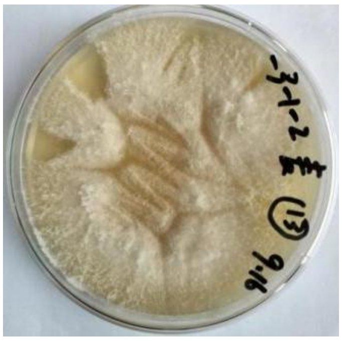
UJNSF0050

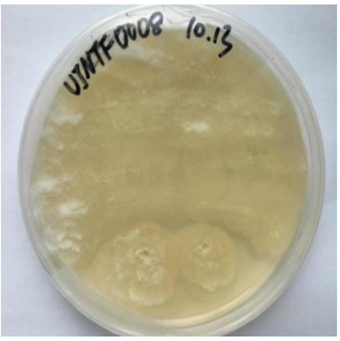

UJNSF0051

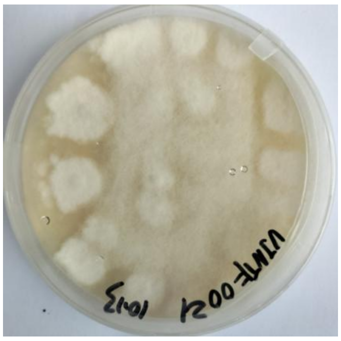
UJNSF0052

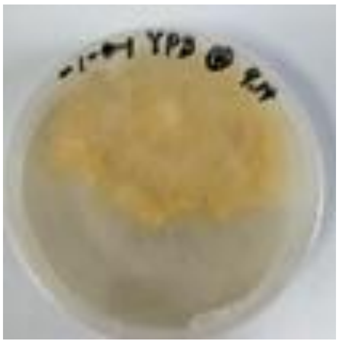
UJNSF0053

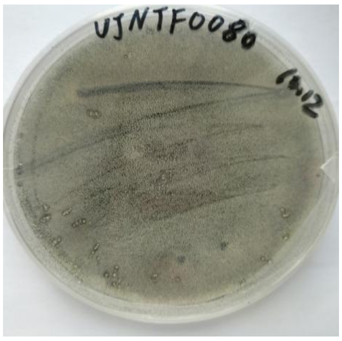

UJNSF0054

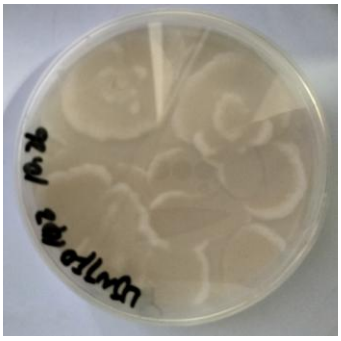
UJNSF0055

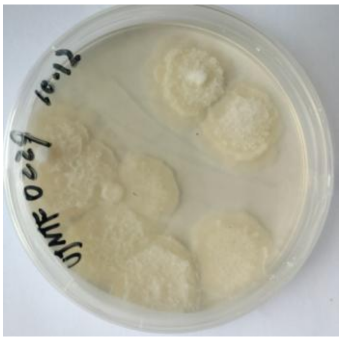
UJNSF0056

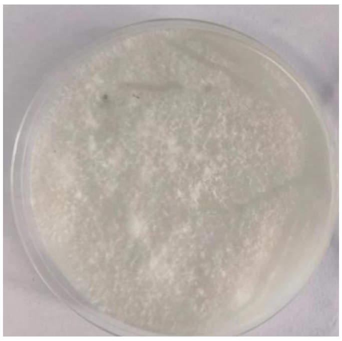
UJNSF0057

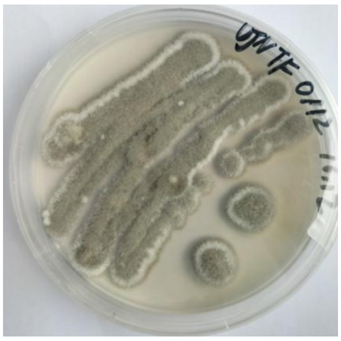

UJNSF0058

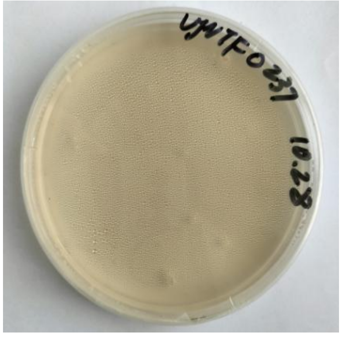

UJNSF0059

2. Phylogenetic analysis independently for 32 fungal genera

Table S1. Evolutionary models for the all genera in the phylogenetic construction

No.	Fungal Genus	Evolutionary model
1	*Penicillium*	T92+I
2	*Aspergillus*	T92+G
3	*Fusarium*	JC+G
4	*Fusicolla*	K2+I
5	*Volutella*	K2+G
6	*Kodamaea*	T92+G+I
7	*Humicola*	JC+I
8	*Condenascus*	JC+G
9	*Tolypocladium*	T92+I
10	*Albophoma*	T92+G
11	*Purpureocillium*	T92+G
12	*Talaromyces*	T92+G
13	*Cladosporium*	K2+I
14	*Subplenodomus*	K2+G
15	*Trichoderma*	K2+I
16	*Apodus*	K2+G
17	*Apiosordaria*	K2+G
18	*Alternaria*	JC+I
19	*Paraboeremia*	K2+I
20	*Coniothyrium*	K2+G
21	*Preussia*	K2+G
22	*Pochonia*	K2+G
23	*Geotrichum*	T92+G
24	*Exophiala*	K2+G
25	*Clonostachys*	K2+I
26	*Linnemannia*	T92+G
27	*Podila*	T92+G+I
28	*Mucor*	T92+G
29	*Mortierella*	T92+G
30	*Bjerkandera*	K2+G
31	*Aaosphaeria*	K2+G
32	*Scytalidium*	K2+G

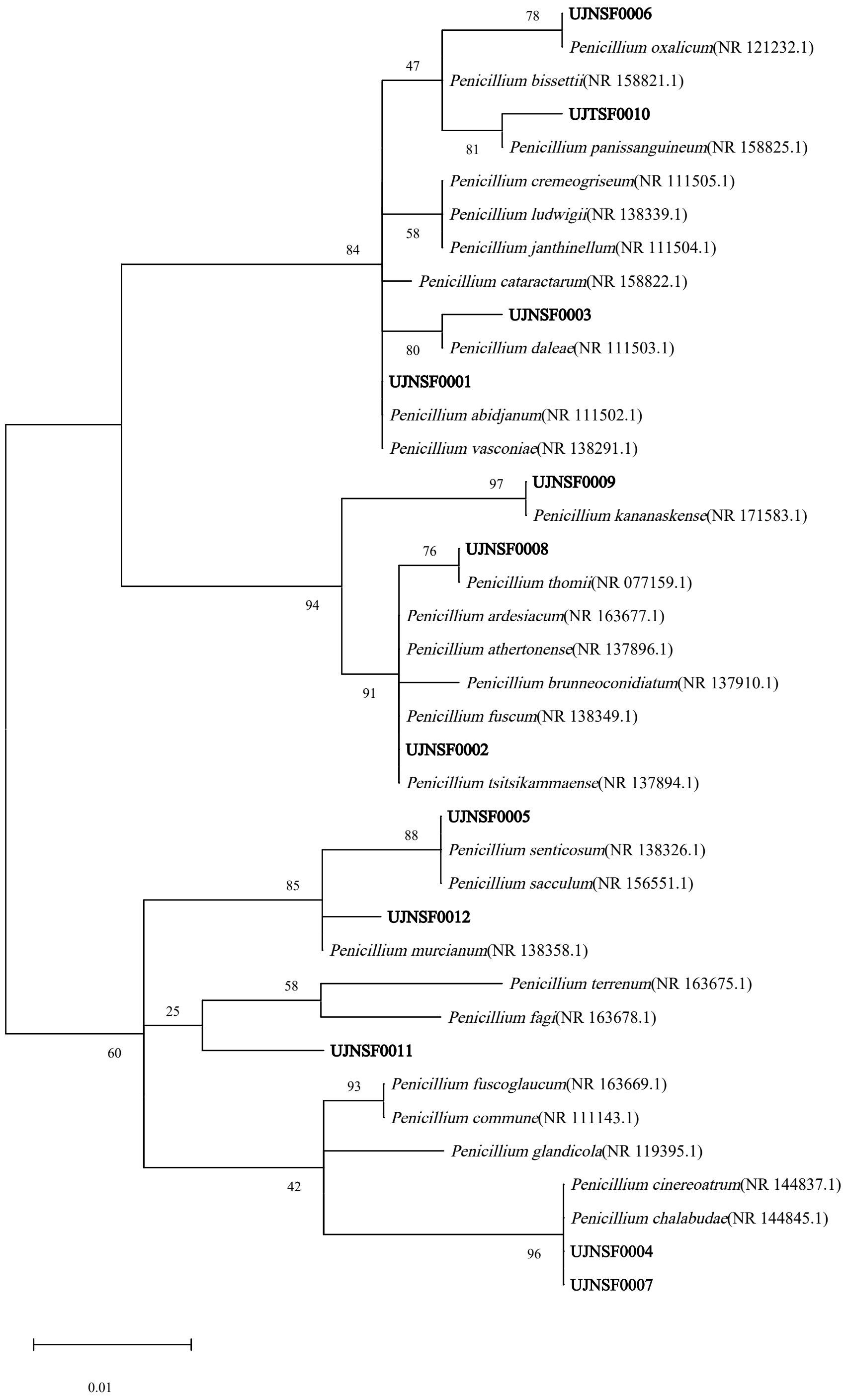
UJNSF0006
Penicillium oxalicum(NR 121232.1)
Penicillium bissettii(NR 158821.1)
UJTSF0010
Penicillium panissanguineum(NR 158825.1)
Penicillium cremeogriseum(NR 111505.1)
Penicillium ludwigii(NR 138339.1)
Penicillium janthinellum(NR 111504.1)
Penicillium cataractarum(NR 158822.1)
UJNSF0003
Penicillium daleae(NR 111503.1)
UJNSF0001
Penicillium abidjanum(NR 111502.1)
Penicillium vasconiae(NR 138291.1)
UJNSF0009
Penicillium kananaskense(NR 171583.1)
UJNSF0008
Penicillium thomii(NR 077159.1)
Penicillium ardesiacum(NR 163677.1)
Penicillium athertonense(NR 137896.1)
Penicillium brunneoconidiatum(NR 137910.1)
Penicillium fuscum(NR 138349.1)
UJNSF0002
Penicillium tsitsikammaense(NR 137894.1)
UJNSF0005
Penicillium senticosum(NR 138326.1)
Penicillium sacculum(NR 156551.1)
UJNSF0012
Penicillium murcianum(NR 138358.1)
Penicillium terrenum(NR 163675.1)
Penicillium fagi(NR 163678.1)
UJNSF0011
Penicillium fuscoglaucum(NR 163669.1)
Penicillium commune(NR 111143.1)
Penicillium glandicola(NR 119395.1)
Penicillium cinereoatrum(NR 144837.1)
Penicillium chalabudae(NR 144845.1)
UJNSF0004
UJNSF0007
78
47
81
58
84
80
97
76
94
91
88
85
58
25
60
93
42
96
0.01

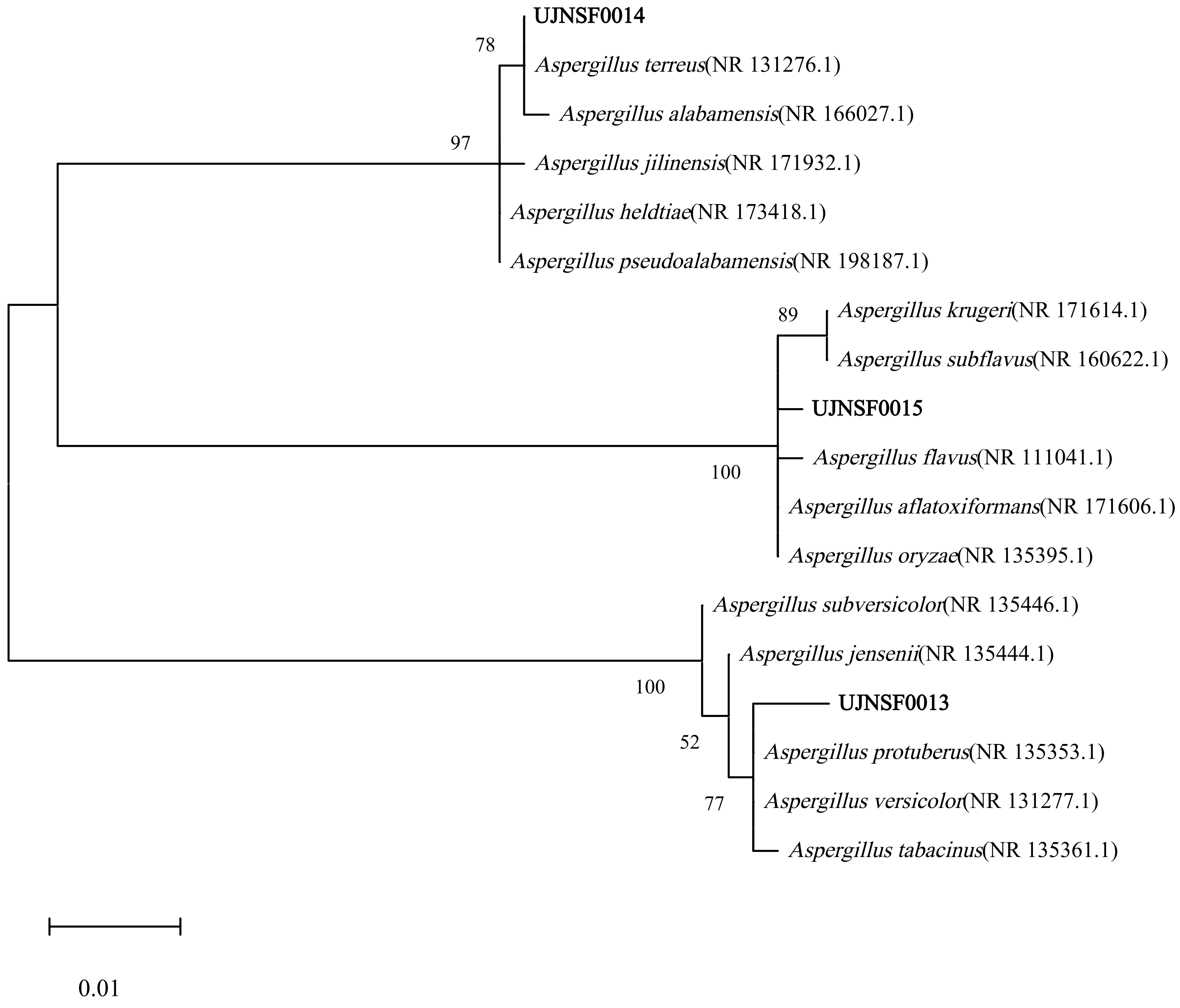
UJNSF0014
Aspergillus terreus(NR 131276.1)
Aspergillus alabamensis(NR 166027.1)
Aspergillus jilinensis(NR 171932.1)
Aspergillus heldtiae(NR 173418.1)
Aspergillus pseudoalabamensis(NR 198187.1)
Aspergillus krugeri(NR 171614.1)
Aspergillus subflavus(NR 160622.1)
UJNSF0015
Aspergillus flavus(NR 111041.1)
Aspergillus aflatoxiformans(NR 171606.1)
Aspergillus oryzae(NR 135395.1)
Aspergillus subversicolor(NR 135446.1)
Aspergillus jensenii(NR 135444.1)
UJNSF0013
Aspergillus protuberus(NR 135353.1)
Aspergillus versicolor(NR 131277.1)
Aspergillus tabacinus(NR 135361.1)
78
97
89
100
100
52
77
0.01

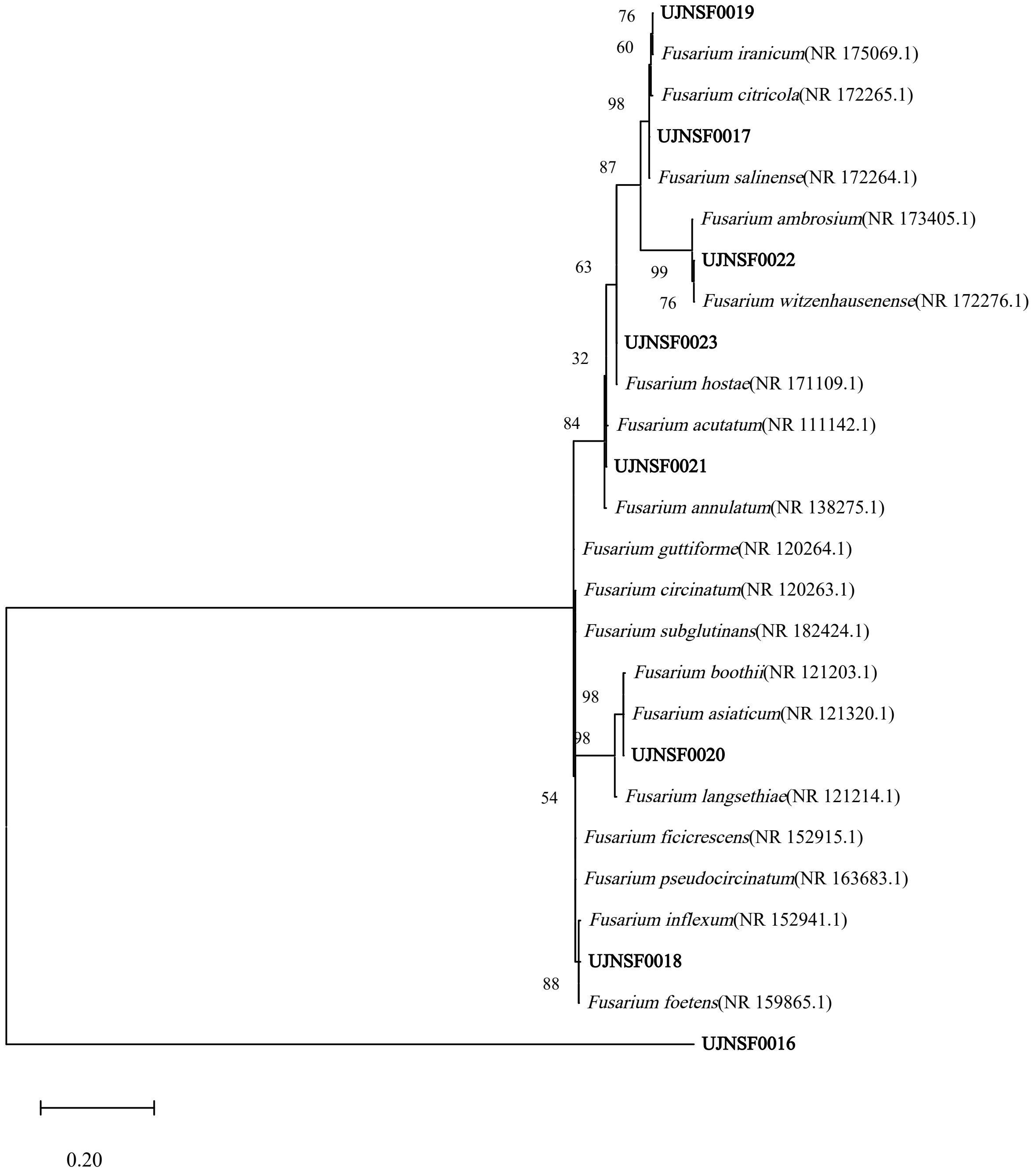

76
UJNSF0019
60
Fusarium iranicum(NR 175069.1)
Fusarium citricola(NR 172265.1)
98
UJNSF0017
87
Fusarium salinense(NR 172264.1)
Fusarium ambrosium(NR 173405.1)
UJNSF0022
63
99
76
Fusarium witzenhausenense(NR 172276.1)
UJNSF0023
32
Fusarium hostae(NR 171109.1)
84
Fusarium acutatum(NR 111142.1)
UJNSF0021
Fusarium annulatum(NR 138275.1)
Fusarium guttiforme(NR 120264.1)
Fusarium circinatum(NR 120263.1)
Fusarium subglutinans(NR 182424.1)
Fusarium boothii(NR 121203.1)
98
Fusarium asiaticum(NR 121320.1)
98
UJNSF0020
54
Fusarium langsethiae(NR 121214.1)
Fusarium ficicrescens(NR 152915.1)
Fusarium pseudocircinatum(NR 163683.1)
Fusarium inflexum(NR 152941.1)
UJNSF0018
88
Fusarium foetens(NR 159865.1)
UJNSF0016
0.20

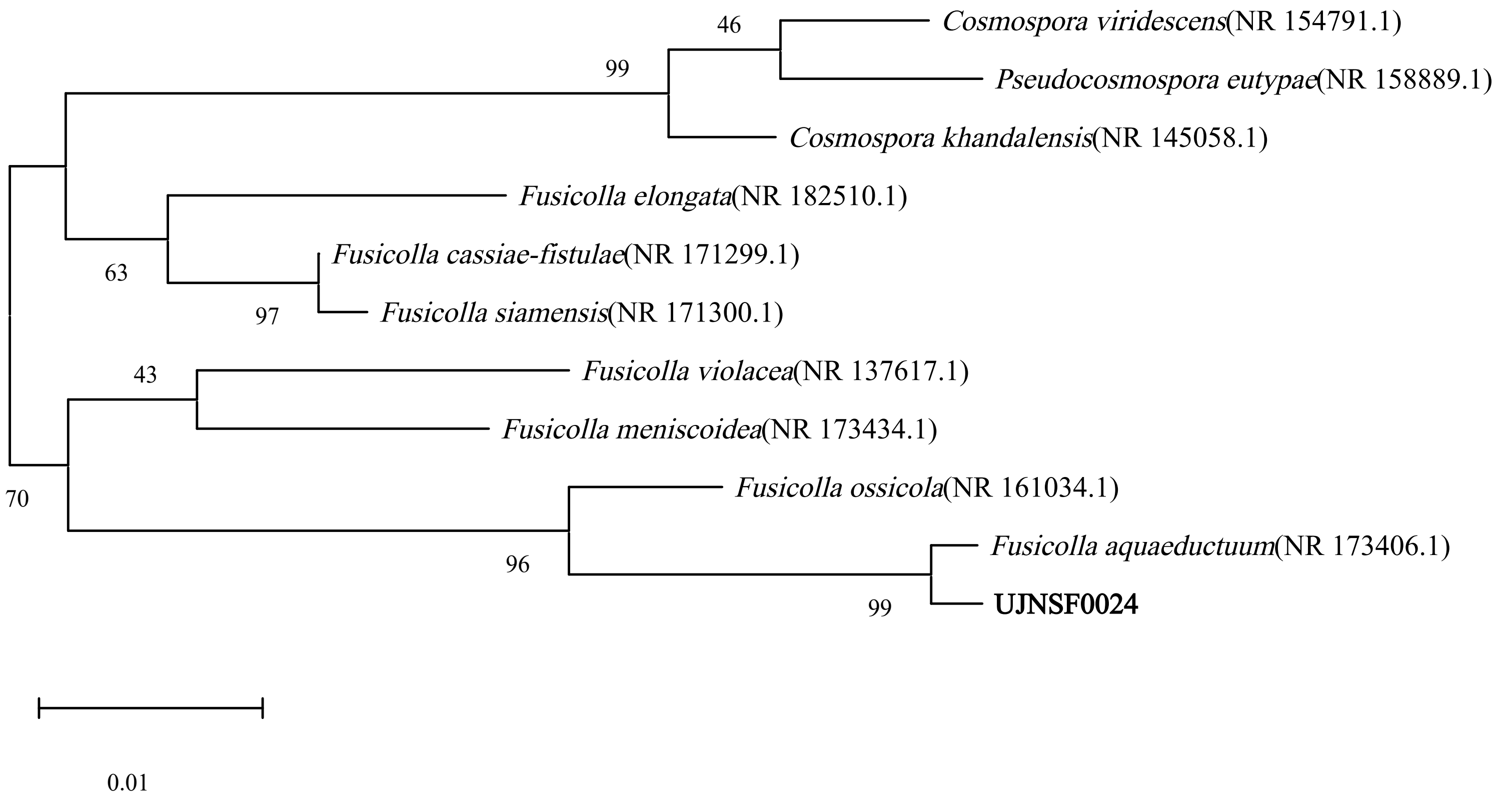
Cosmospora viridescens(NR 154791.1)
Pseudocosmospora eutypae(NR 158889.1)
Cosmospora khandalensis(NR 145058.1)
Fusicolla elongata(NR 182510.1)
Fusicolla cassiae-fistulae(NR 171299.1)
Fusicolla siamensis(NR 171300.1)
Fusicolla violacea(NR 137617.1)
Fusicolla meniscoidea(NR 173434.1)
Fusicolla ossicola(NR 161034.1)
Fusicolla aquaeductuum(NR 173406.1)
UJNSF0024
46
99
63
97
43
70
96
99
0.01

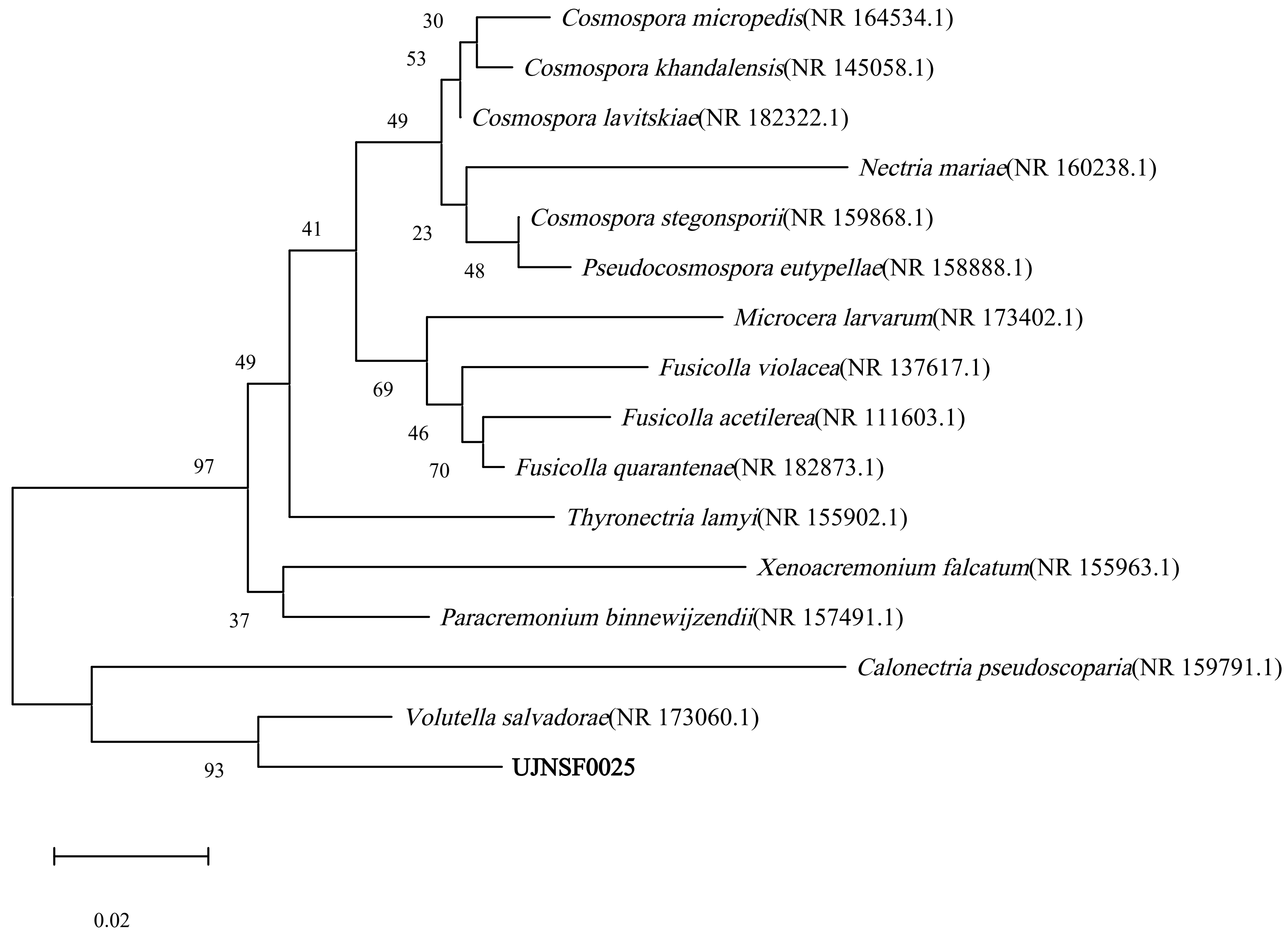
30
Cosmospora micropedis(NR 164534.1)
53
Cosmospora khandalensis(NR 145058.1)
49
Cosmospora lavitskiae(NR 182322.1)
Nectria mariae(NR 160238.1)
41
23
Cosmospora stegonsporii(NR 159868.1)
48
Pseudocosmospora eutypellae(NR 158888.1)
Microcera larvarum(NR 173402.1)
49
Fusicolla violacea(NR 137617.1)
69
Fusicolla acetilerea(NR 111603.1)
46
97
70
Fusicolla quarantenae(NR 182873.1)
Thyronectria lamyi(NR 155902.1)
Xenoacremonium falcatum(NR 155963.1)
37
Paracremonium binnewijzendii(NR 157491.1)
Calonectria pseudoscoparia(NR 159791.1)
Volutella salvadorae(NR 173060.1)
93
UJNSF0025
0.02

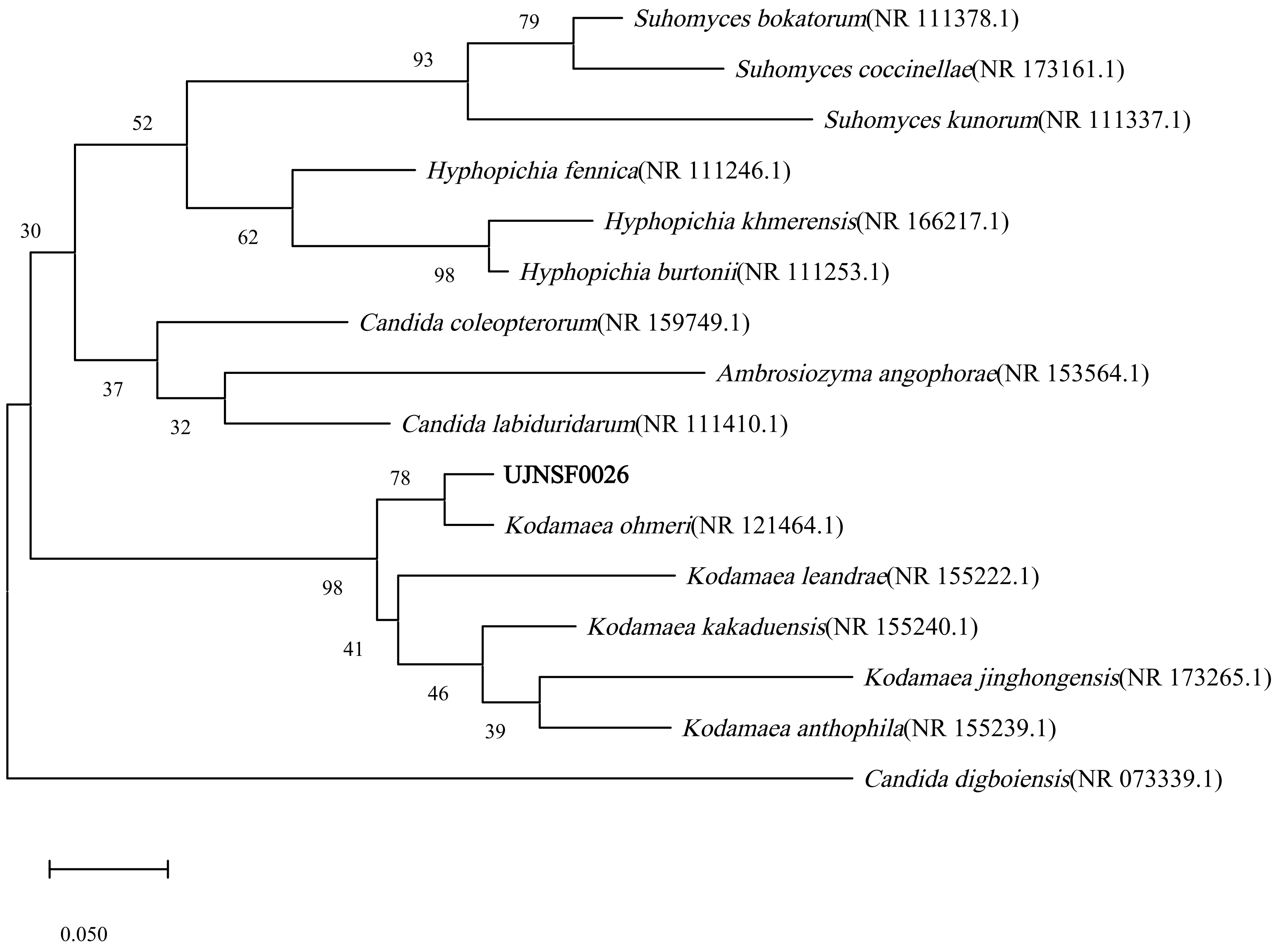
79
93
52
62
98
30
37
32
78
98
41
46
39
Suhomyces bokatorum(NR 111378.1)
Suhomyces coccinellae(NR 173161.1)
Suhomyces kunorum(NR 111337.1)
Hyphopichia fennica(NR 111246.1)
Hyphopichia khmerensis(NR 166217.1)
Hyphopichia burtonii(NR 111253.1)
Candida coleopterorum(NR 159749.1)
Ambrosiozyma angophorae(NR 153564.1)
Candida labiduridarum(NR 111410.1)
UJNSF0026
Kodamaea ohmeri(NR 121464.1)
Kodamaea leandrae(NR 155222.1)
Kodamaea kakaduensis(NR 155240.1)
Kodamaea jinghongensis(NR 173265.1)
Kodamaea anthophila(NR 155239.1)
Candida digboiensis(NR 073339.1)
0.050

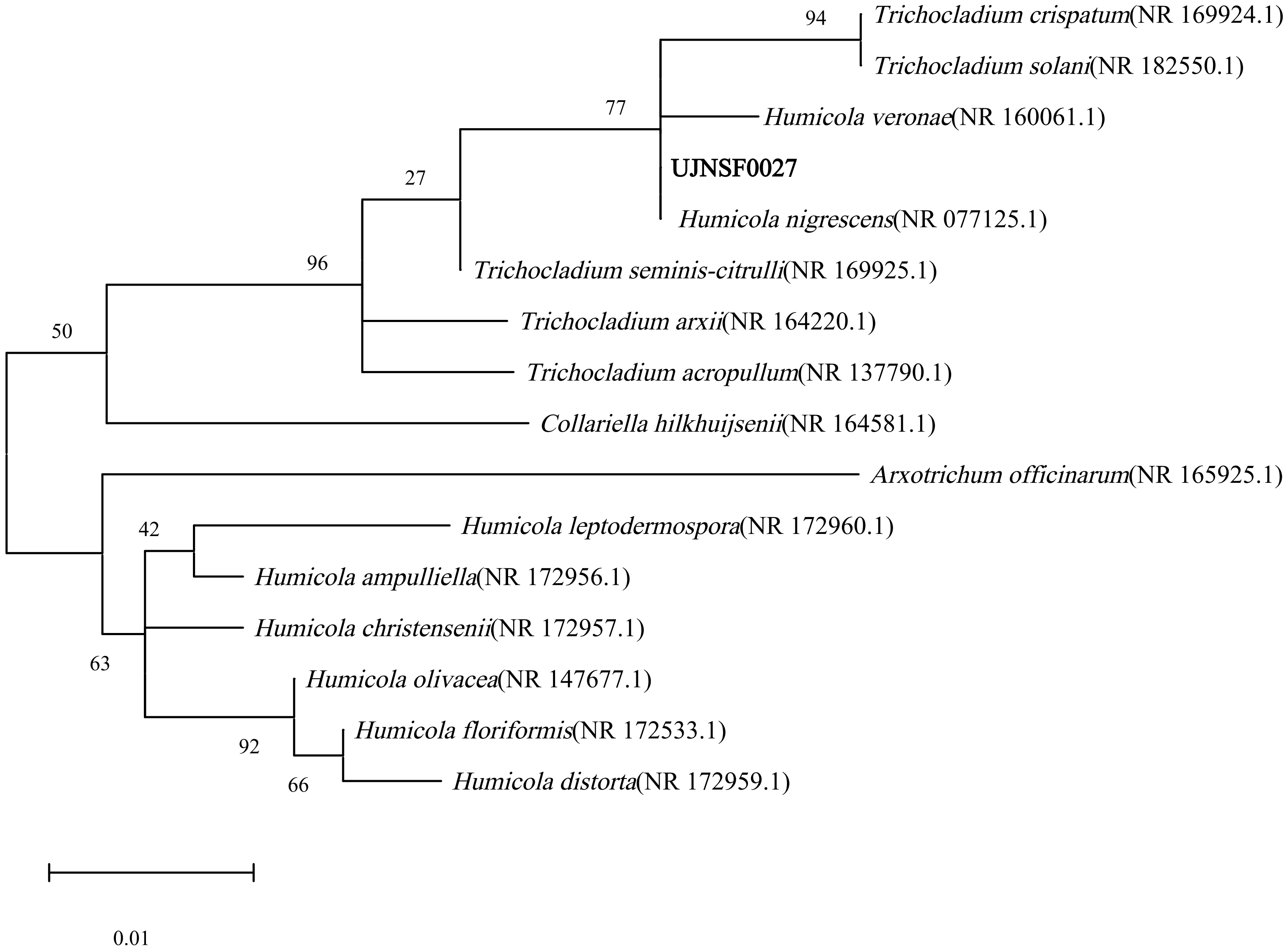
94
Trichocladium crispatum(NR 169924.1)
Trichocladium solani(NR 182550.1)
77
Humicola veronae(NR 160061.1)
27
UJNSF0027
Humicola nigrescens(NR 077125.1)
96
Trichocladium seminis-citrulli(NR 169925.1)
50
Trichocladium arxii(NR 164220.1)
Trichocladium acropullum(NR 137790.1)
Collariella hilkhuijsenii(NR 164581.1)
Arxotrichum officinarum(NR 165925.1)
42
Humicola leptodermospora(NR 172960.1)
Humicola ampulliella(NR 172956.1)
Humicola christensenii(NR 172957.1)
63
Humicola olivacea(NR 147677.1)
Humicola floriformis(NR 172533.1)
92
66
Humicola distorta(NR 172959.1)
0.01

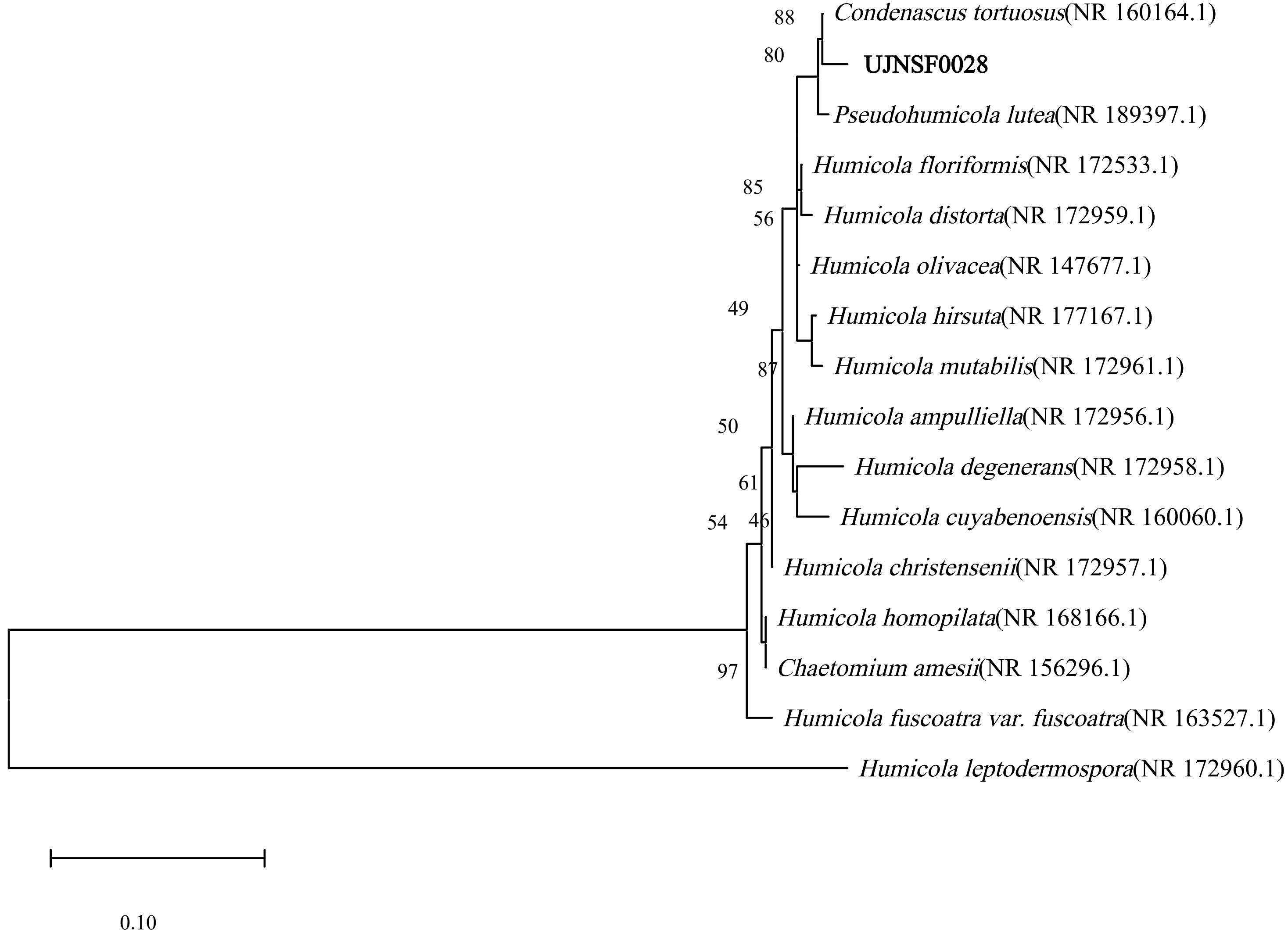
Condenascus tortuosus(NR 160164.1)
UJNSF0028
Pseudohumicola lutea(NR 189397.1)
Humicola floriformis(NR 172533.1)
Humicola distorta(NR 172959.1)
Humicola olivacea(NR 147677.1)
Humicola hirsuta(NR 177167.1)
Humicola mutabilis(NR 172961.1)
Humicola ampulliella(NR 172956.1)
Humicola degenerans(NR 172958.1)
Humicola cuyabenoensis(NR 160060.1)
Humicola christensenii(NR 172957.1)
Humicola homopilata(NR 168166.1)
Chaetomium amesii(NR 156296.1)
Humicola fuscoatra var. fuscoatra(NR 163527.1)
Humicola leptodermospora(NR 172960.1)
88
80
85
56
49
87
50
61
54
46
97
0.10

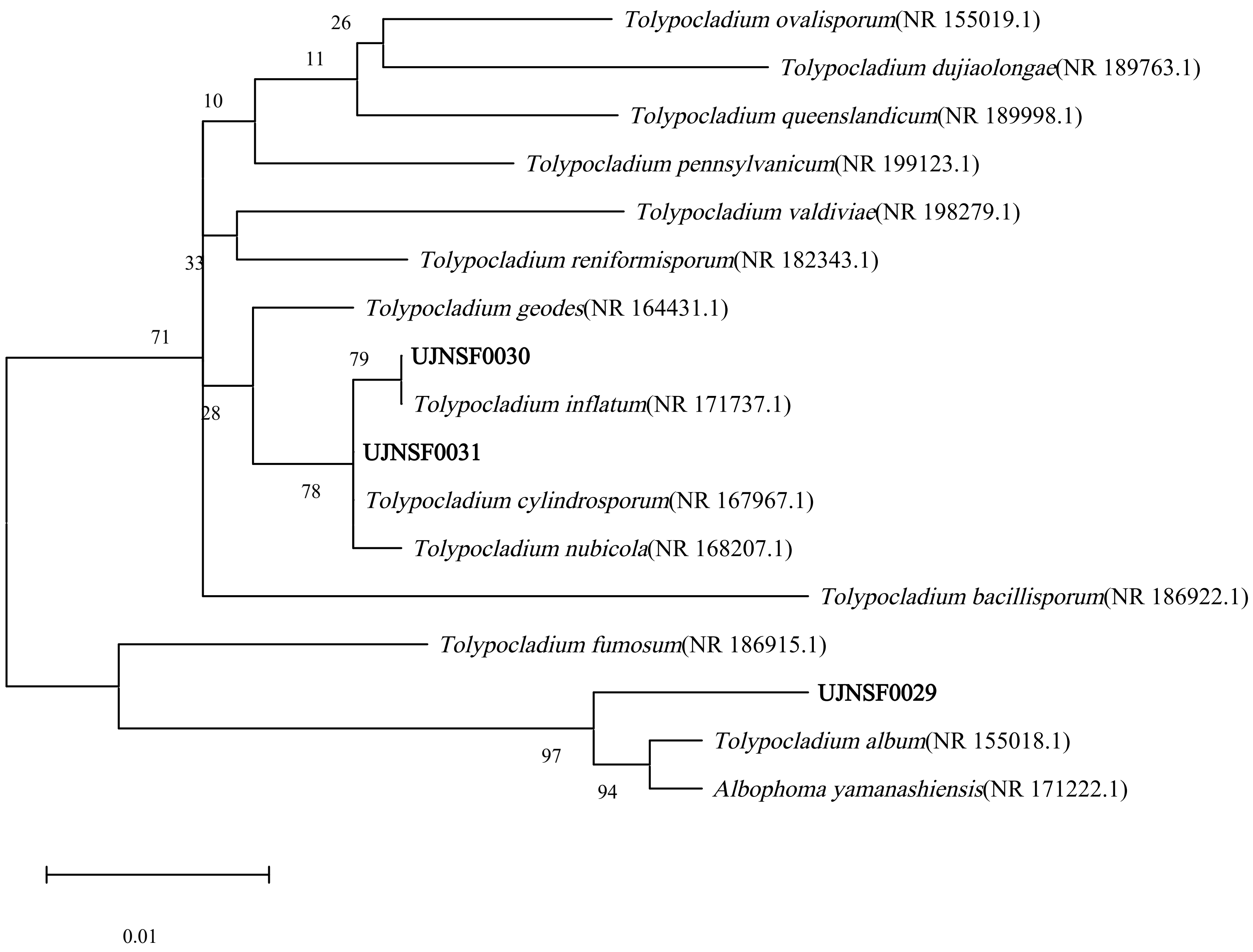

26
11
10
33
71
28
79
78
97
94
Tolypocladium ovalisporum(NR 155019.1)
Tolypocladium dujiaolongae(NR 189763.1)
Tolypocladium queenslandicum(NR 189998.1)
Tolypocladium pennsylvanicum(NR 199123.1)
Tolypocladium valdiviae(NR 198279.1)
Tolypocladium reniformisporum(NR 182343.1)
Tolypocladium geodes(NR 164431.1)
UJNSF0030
Tolypocladium inflatum(NR 171737.1)
UJNSF0031
Tolypocladium cylindrosporum(NR 167967.1)
Tolypocladium nubicola(NR 168207.1)
Tolypocladium bacillisporum(NR 186922.1)
Tolypocladium fumosum(NR 186915.1)
UJNSF0029
Tolypocladium album(NR 155018.1)
Albophoma yamanashiensis(NR 171222.1)
0.01

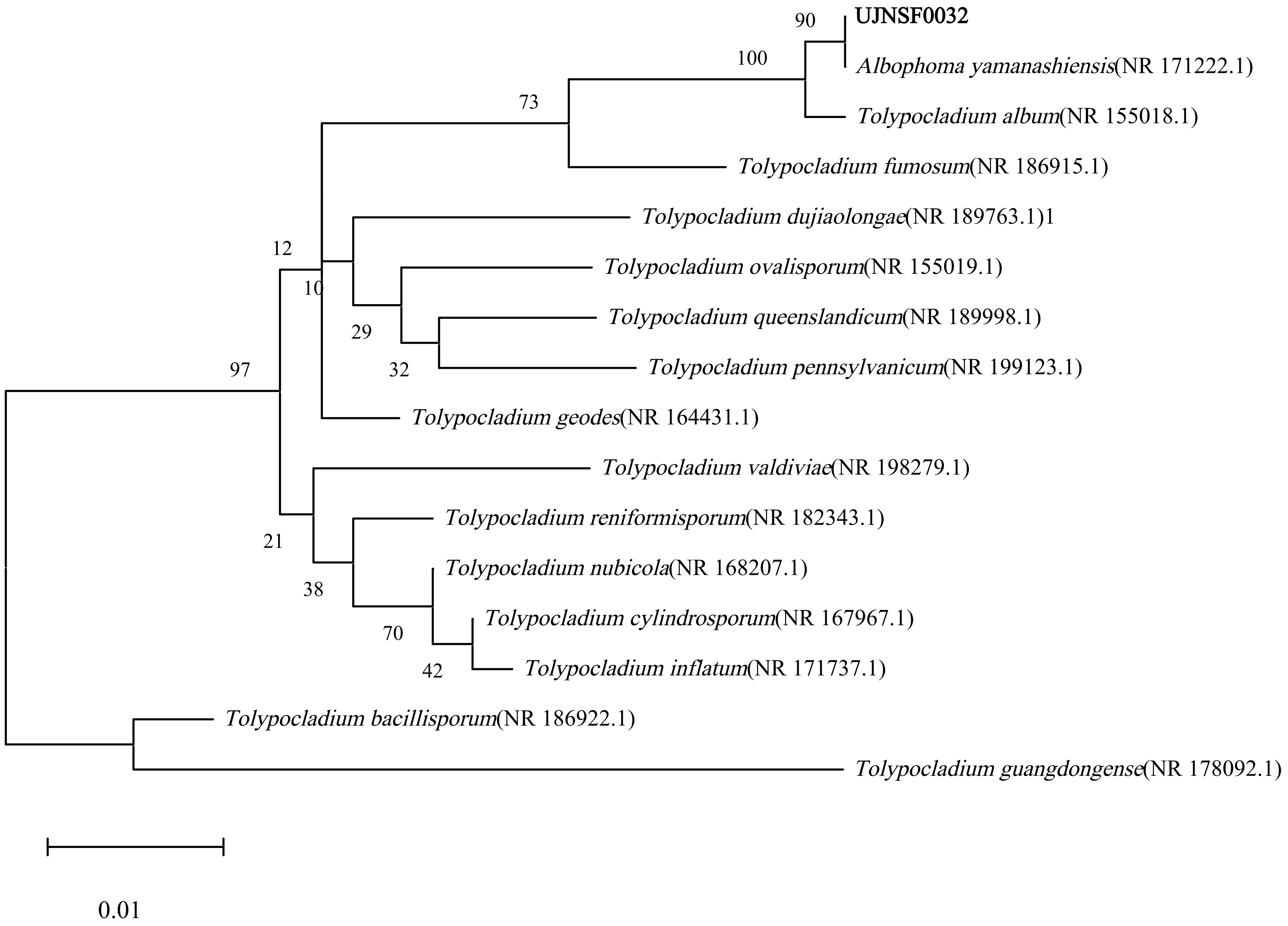
UJNSF0032
Albophoma yamanashiensis(NR 171222.1)
Tolypocladium album(NR 155018.1)
Tolypocladium fumosum(NR 186915.1)
Tolypocladium dujiaolongae(NR 189763.1)1
Tolypocladium ovalisporum(NR 155019.1)
Tolypocladium queenslandicum(NR 189998.1)
Tolypocladium pennsylvanicum(NR 199123.1)
Tolypocladium geodes(NR 164431.1)
Tolypocladium valdiviae(NR 198279.1)
Tolypocladium reniformisporum(NR 182343.1)
Tolypocladium nubicola(NR 168207.1)
Tolypocladium cylindrosporum(NR 167967.1)
Tolypocladium inflatum(NR 171737.1)
Tolypocladium bacillisporum(NR 186922.1)
Tolypocladium guangdongense(NR 178092.1)
90
100
73
12
10
29
32
97
21
38
70
42
0.01

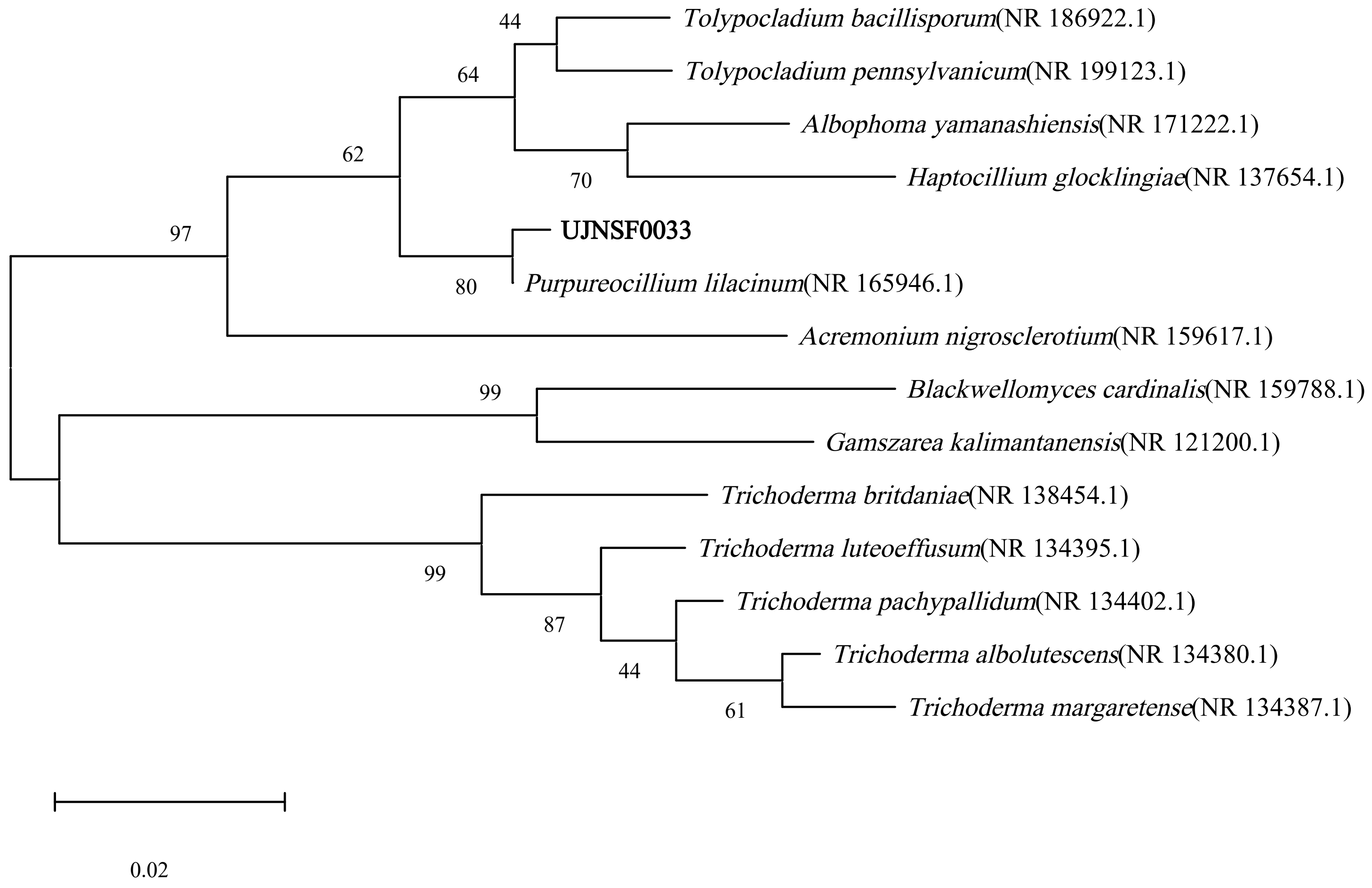
Tolypocladium bacillisporum(NR 186922.1)
Tolypocladium pennsylvanicum(NR 199123.1)
Albophoma yamanashiensis(NR 171222.1)
Haptocillium glocklingiae(NR 137654.1)
UJNSF0033
Purpureocillium lilacinum(NR 165946.1)
Acremonium nigrosclerotium(NR 159617.1)
Blackwellomyces cardinalis(NR 159788.1)
Gamszarea kalimantanensis(NR 121200.1)
Trichoderma britdaniae(NR 138454.1)
Trichoderma luteoeffusum(NR 134395.1)
Trichoderma pachypallidum(NR 134402.1)
Trichoderma albolutescens(NR 134380.1)
Trichoderma margaretense(NR 134387.1)
44
64
62
70
97
80
99
99
87
44
61
0.02

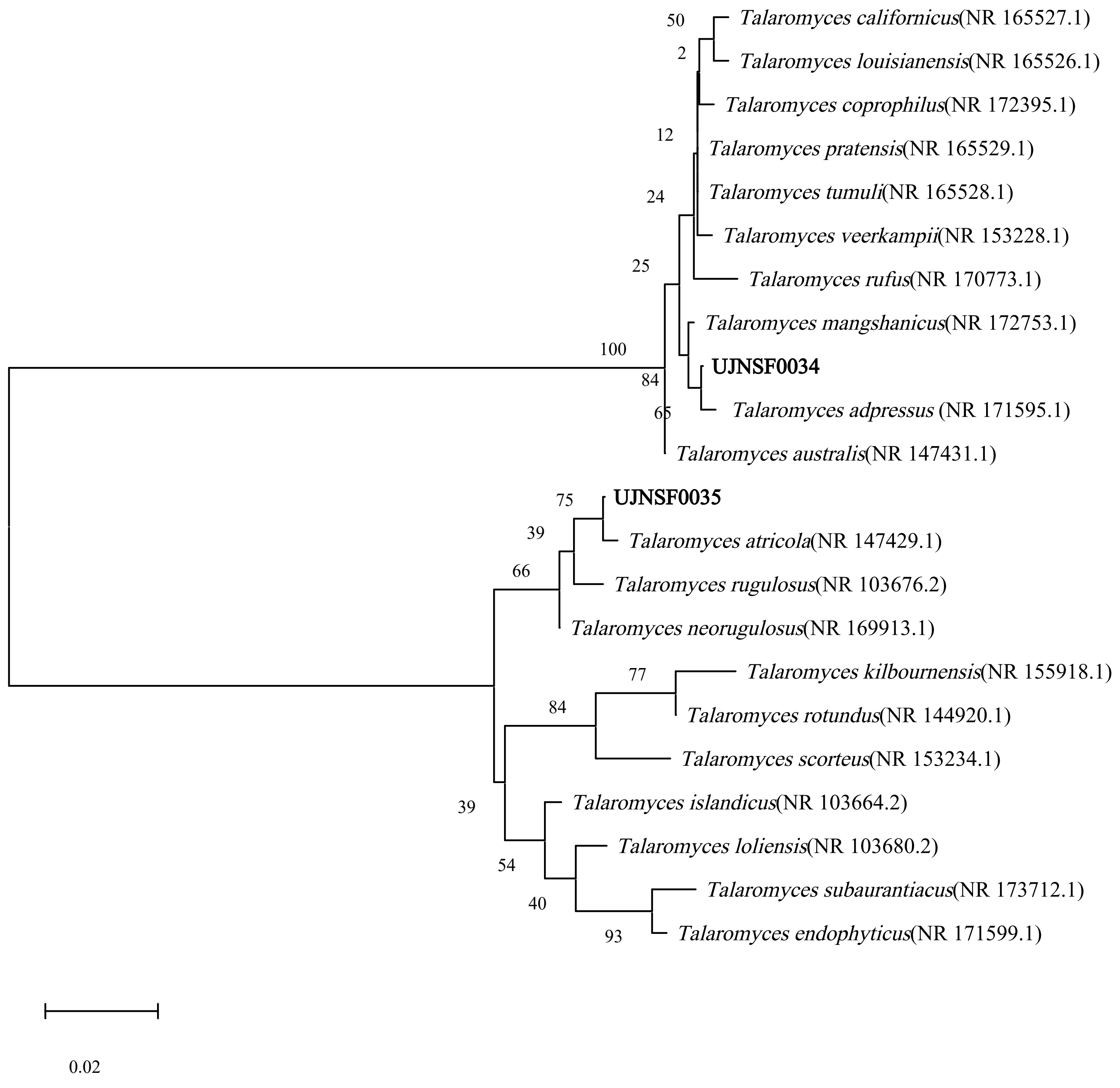
50
2
12
24
25
100
84
65
Talaromyces californicus(NR 165527.1)
Talaromyces louisianensis(NR 165526.1)
Talaromyces coprophilus(NR 172395.1)
Talaromyces pratensis(NR 165529.1)
Talaromyces tumuli(NR 165528.1)
Talaromyces veerkampii(NR 153228.1)
Talaromyces rufus(NR 170773.1)
Talaromyces mangshanicus(NR 172753.1)
UJNSF0034
Talaromyces adpressus (NR 171595.1)
Talaromyces australis(NR 147431.1)
75
39
66
UJNSF0035
Talaromyces atricola(NR 147429.1)
Talaromyces rugulosus(NR 103676.2)
Talaromyces neorugulosus(NR 169913.1)
77
84
Talaromyces kilbournensis(NR 155918.1)
Talaromyces rotundus(NR 144920.1)
Talaromyces scorteus(NR 153234.1)
39
54
40
93
Talaromyces islandicus(NR 103664.2)
Talaromyces loliensis(NR 103680.2)
Talaromyces subaurantiacus(NR 173712.1)
Talaromyces endophyticus(NR 171599.1)
0.02

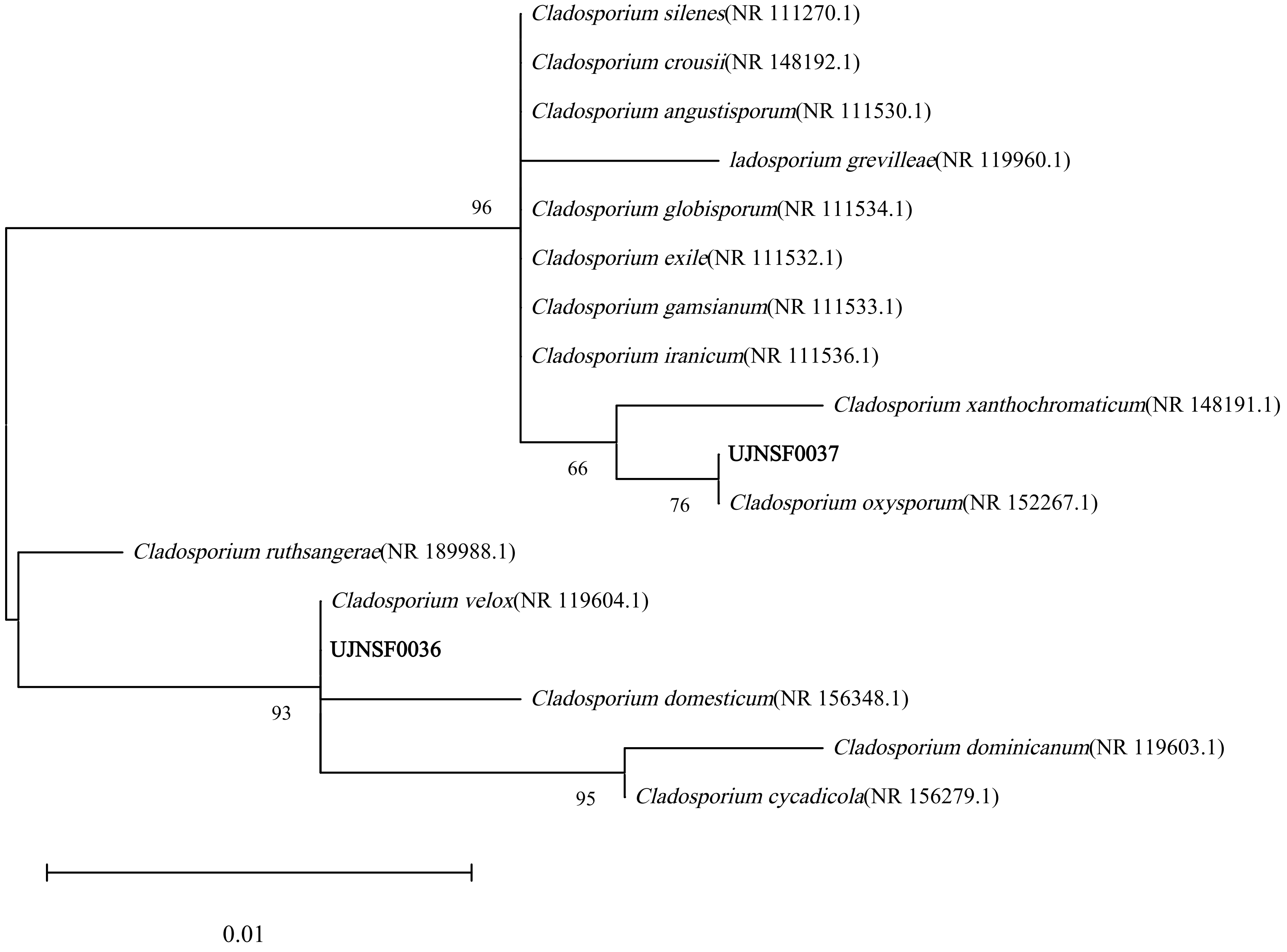
Cladosporium silenes(NR 111270.1)
Cladosporium crousii(NR 148192.1)
Cladosporium angustisporum(NR 111530.1)
ladosporium grevilleae(NR 119960.1)
96
Cladosporium globisporum(NR 111534.1)
Cladosporium exile(NR 111532.1)
Cladosporium gamsianum(NR 111533.1)
Cladosporium iranicum(NR 111536.1)
Cladosporium xanthochromaticum(NR 148191.1)
UJNSF0037
66
76
Cladosporium oxysporum(NR 152267.1)
Cladosporium ruthsangerae(NR 189988.1)
Cladosporium velox(NR 119604.1)
UJNSF0036
Cladosporium domesticum(NR 156348.1)
93
Cladosporium dominicanum(NR 119603.1)
95
Cladosporium cycadicola(NR 156279.1)
0.01

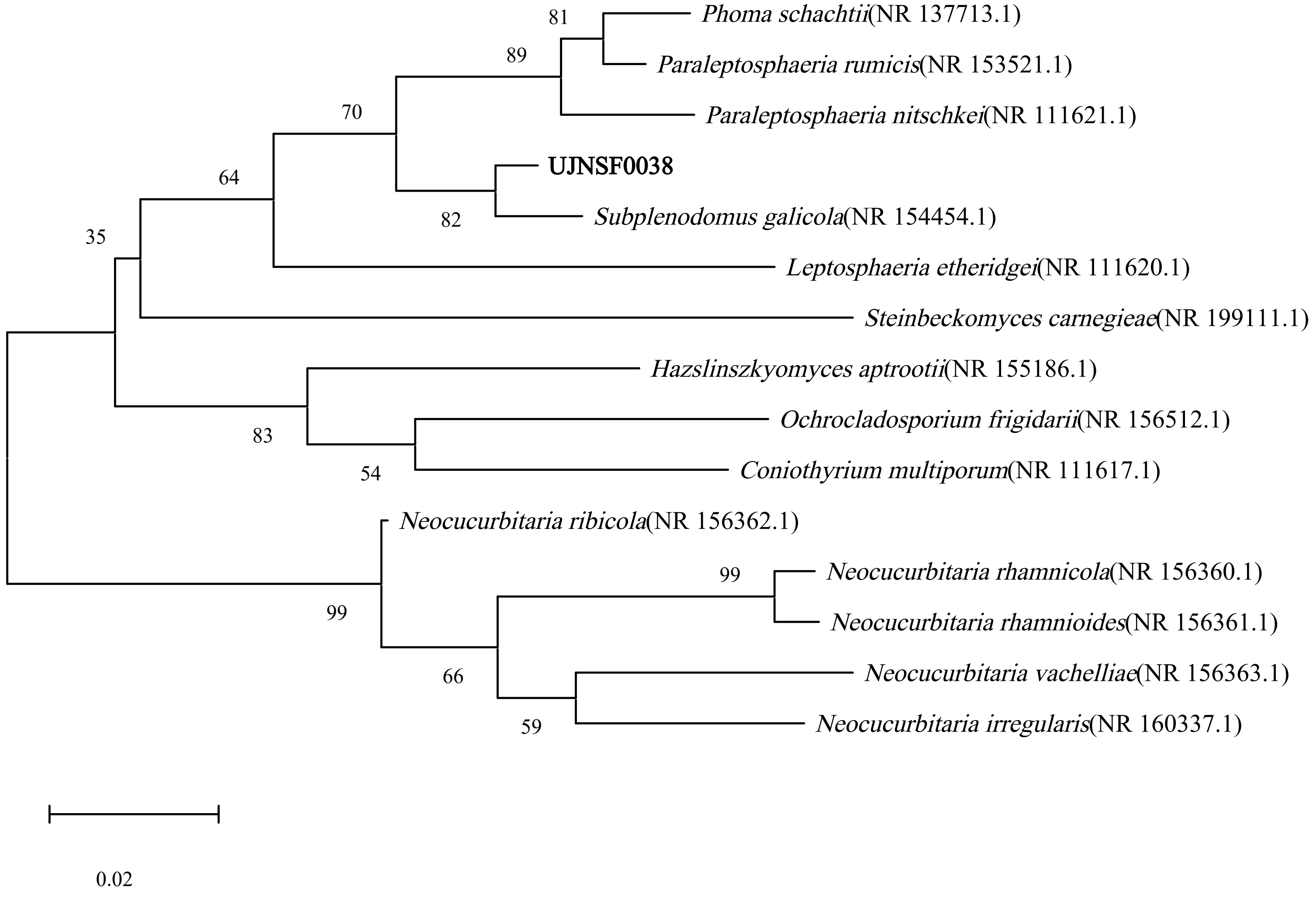
81
89
70
64
35
82
83
54
99
99
66
59
Phoma schachtii(NR 137713.1)
Paraleptosphaeria rumicis(NR 153521.1)
Paraleptosphaeria nitschkei(NR 111621.1)
UJNSF0038
Subplenodomus galicola(NR 154454.1)
Leptosphaeria etheridgei(NR 111620.1)
Steinbeckomyces carnegieae(NR 199111.1)
Hazslinszkyomyces aptrootii(NR 155186.1)
Ochrocladosporium frigidarii(NR 156512.1)
Coniothyrium multiporum(NR 111617.1)
Neocucurbitaria ribicola(NR 156362.1)
Neocucurbitaria rhamnicola(NR 156360.1)
Neocucurbitaria rhamnioides(NR 156361.1)
Neocucurbitaria vachelliae(NR 156363.1)
Neocucurbitaria irregularis(NR 160337.1)
0.02

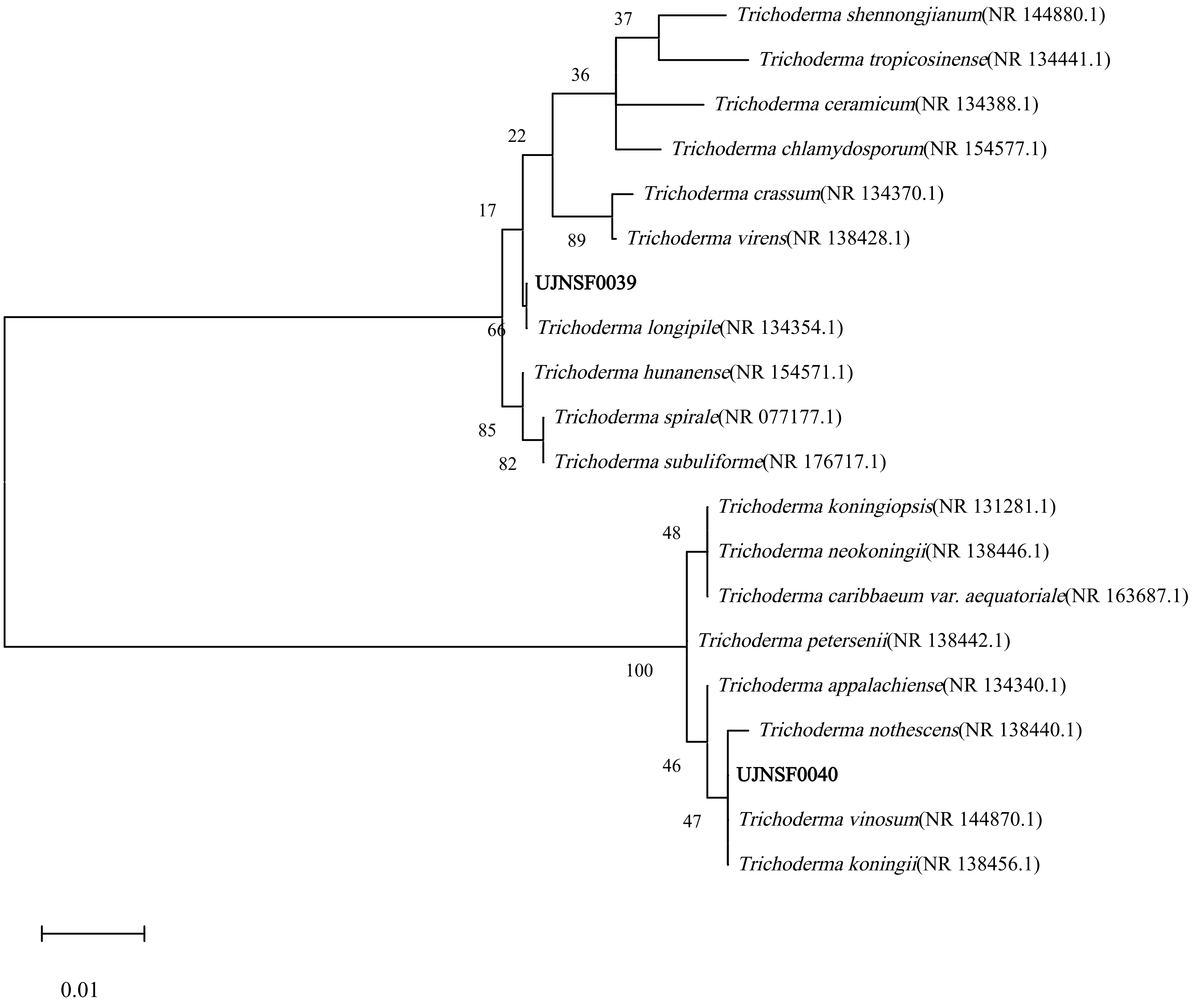
Trichoderma shennongjianum(NR 144880.1)
Trichoderma tropicosinense(NR 134441.1)
Trichoderma ceramicum(NR 134388.1)
Trichoderma chlamydosporum(NR 154577.1)
Trichoderma crassum(NR 134370.1)
Trichoderma virens(NR 138428.1)
UJNSF0039
Trichoderma longipile(NR 134354.1)
Trichoderma hunanense(NR 154571.1)
Trichoderma spirale(NR 077177.1)
Trichoderma subuliforme(NR 176717.1)
Trichoderma koningiopsis(NR 131281.1)
Trichoderma neokoningii(NR 138446.1)
Trichoderma caribbaeum var. aequatoriale(NR 163687.1)
Trichoderma petersenii(NR 138442.1)
Trichoderma appalachiense(NR 134340.1)
Trichoderma nothescens(NR 138440.1)
UJNSF0040
Trichoderma vinosum(NR 144870.1)
Trichoderma koningii(NR 138456.1)
37
36
22
17
89
66
85
82
48
100
46
47
0.01

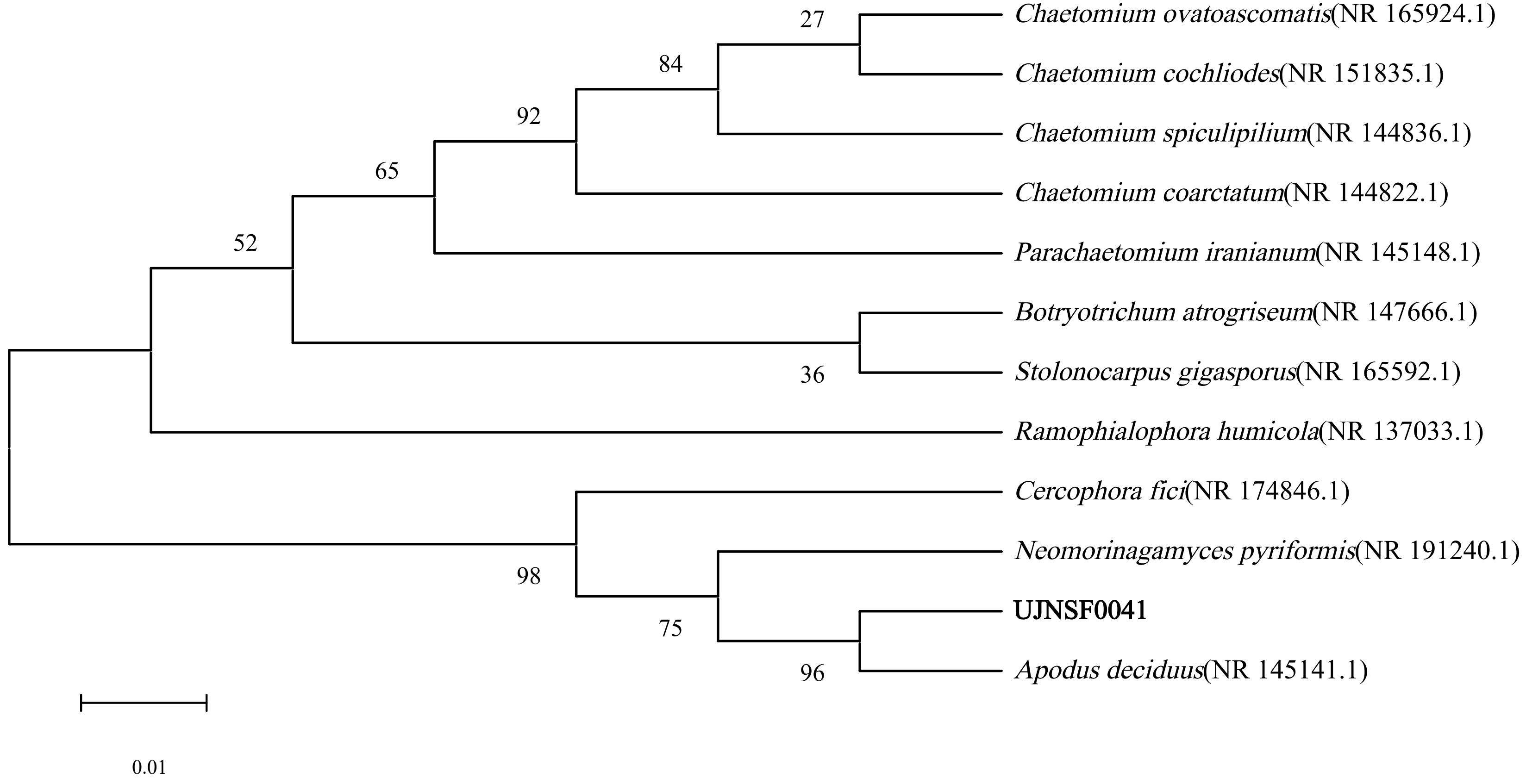
Chaetomium ovatoascomatis(NR 165924.1)
Chaetomium cochliodes(NR 151835.1)
Chaetomium spiculipilium(NR 144836.1)
Chaetomium coarctatum(NR 144822.1)
Parachaetomium iranianum(NR 145148.1)
Botryotrichum atrogriseum(NR 147666.1)
Stolonocarpus gigasporus(NR 165592.1)
Ramophialophora humicola(NR 137033.1)
Cercophora fici(NR 174846.1)
Neomorinagamyces pyriformis(NR 191240.1)
UJNSF0041
Apodus deciduus(NR 145141.1)
27
84
92
65
52
36
98
75
96
0.01

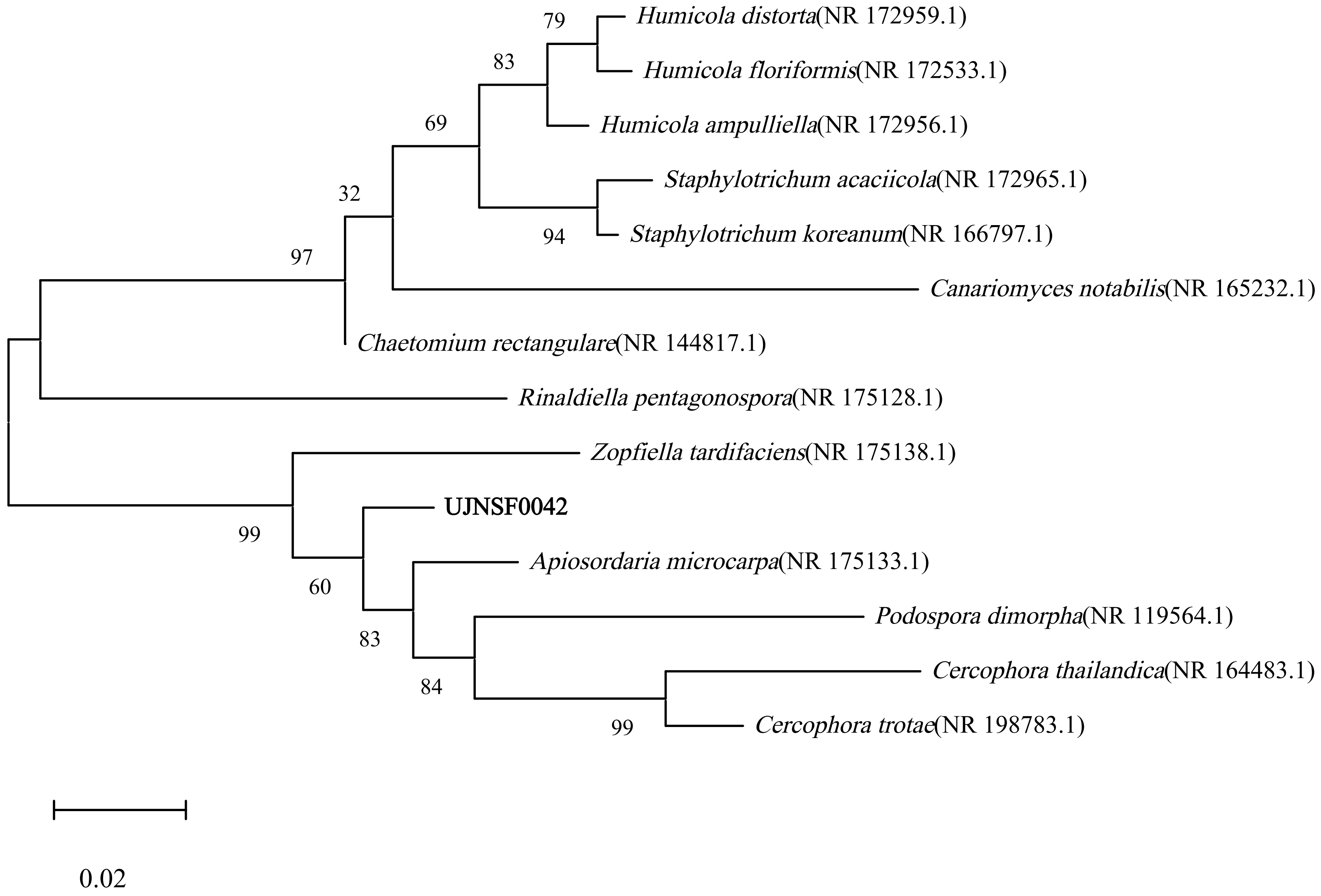

79
83
69
32
97
94
99
60
83
84
99
Humicola distorta(NR 172959.1)
Humicola floriformis(NR 172533.1)
Humicola ampulliella(NR 172956.1)
Staphylotrichum acaciicola(NR 172965.1)
Staphylotrichum koreanum(NR 166797.1)
Canariomyces notabilis(NR 165232.1)
Chaetomium rectangulare(NR 144817.1)
Rinaldiella pentagonospora(NR 175128.1)
Zopfiella tardifaciens(NR 175138.1)
UJNSF0042
Apiosordaria microcarpa(NR 175133.1)
Podospora dimorpha(NR 119564.1)
Cercophora thailandica(NR 164483.1)
Cercophora trotae(NR 198783.1)
0.02

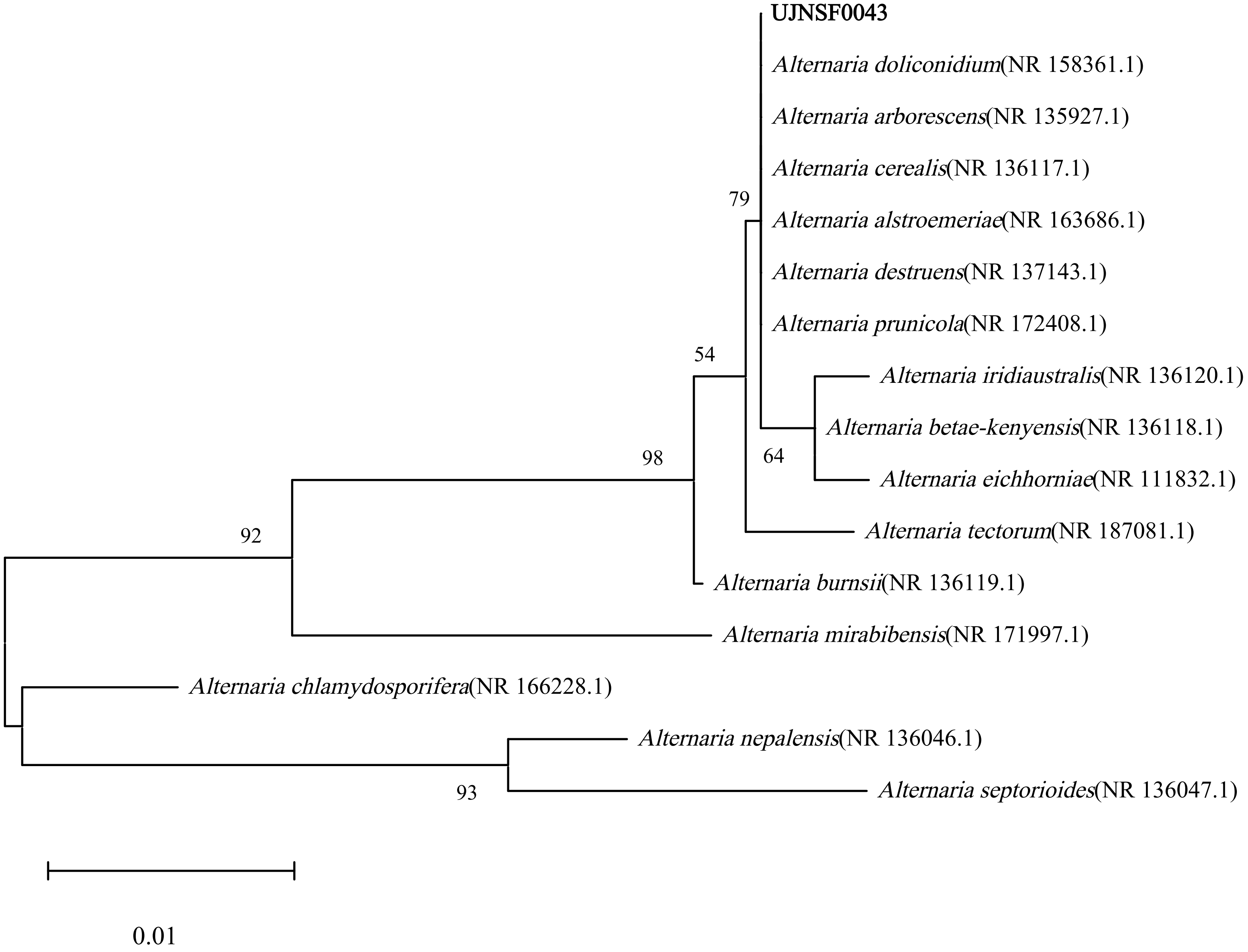
UJNSF0043
Alternaria doliconidium(NR 158361.1)
Alternaria arborescens(NR 135927.1)
Alternaria cerealis(NR 136117.1)
79
Alternaria alstroemeriae(NR 163686.1)
Alternaria destruens(NR 137143.1)
Alternaria prunicola(NR 172408.1)
54
Alternaria iridiaustralis(NR 136120.1)
Alternaria betae-kenyensis(NR 136118.1)
98
64
Alternaria eichhorniae(NR 111832.1)
Alternaria tectorum(NR 187081.1)
92
Alternaria burnsii(NR 136119.1)
Alternaria mirabibensis(NR 171997.1)
Alternaria chlamydosporifera(NR 166228.1)
Alternaria nepalensis(NR 136046.1)
93
Alternaria septorioides(NR 136047.1)
0.01

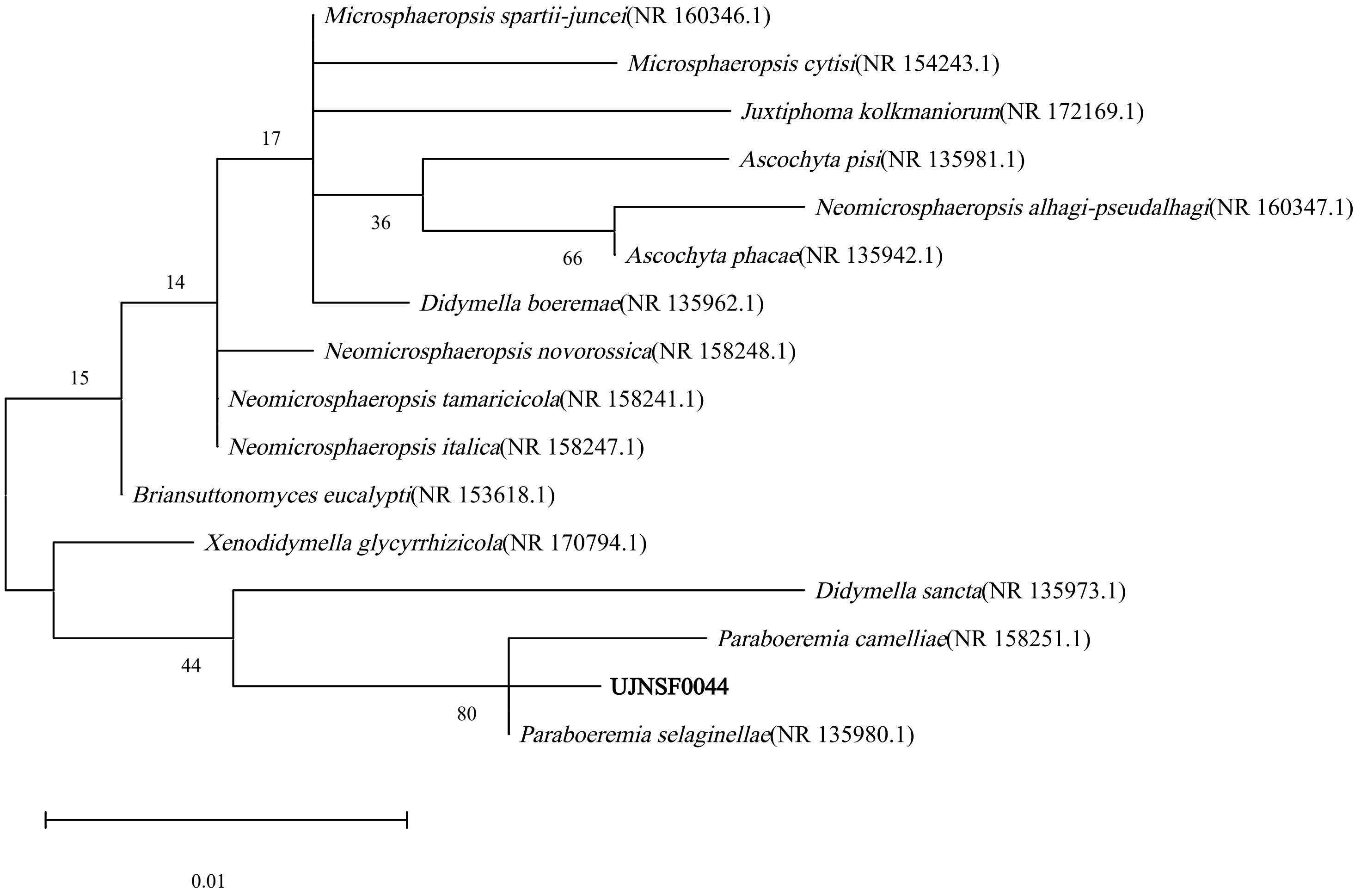
Microsphaeropsis spartii-juncei(NR 160346.1)
Microsphaeropsis cytisi(NR 154243.1)
Juxtiphoma kolkmaniorum(NR 172169.1)
17
Ascochyta pisi(NR 135981.1)
Neomicrosphaeropsis alhagi-pseudalhagi(NR 160347.1)
36
66
Ascochyta phacae(NR 135942.1)
14
Didymella boeremae(NR 135962.1)
Neomicrosphaeropsis novorossica(NR 158248.1)
15
Neomicrosphaeropsis tamaricicola(NR 158241.1)
Neomicrosphaeropsis italica(NR 158247.1)
Briansuttonomyces eucalypti(NR 153618.1)
Xenodidymella glycyrrhizicola(NR 170794.1)
Didymella sancta(NR 135973.1)
Paraboeremia camelliae(NR 158251.1)
44
UJNSF0044
80
Paraboeremia selaginellae(NR 135980.1)
0.01

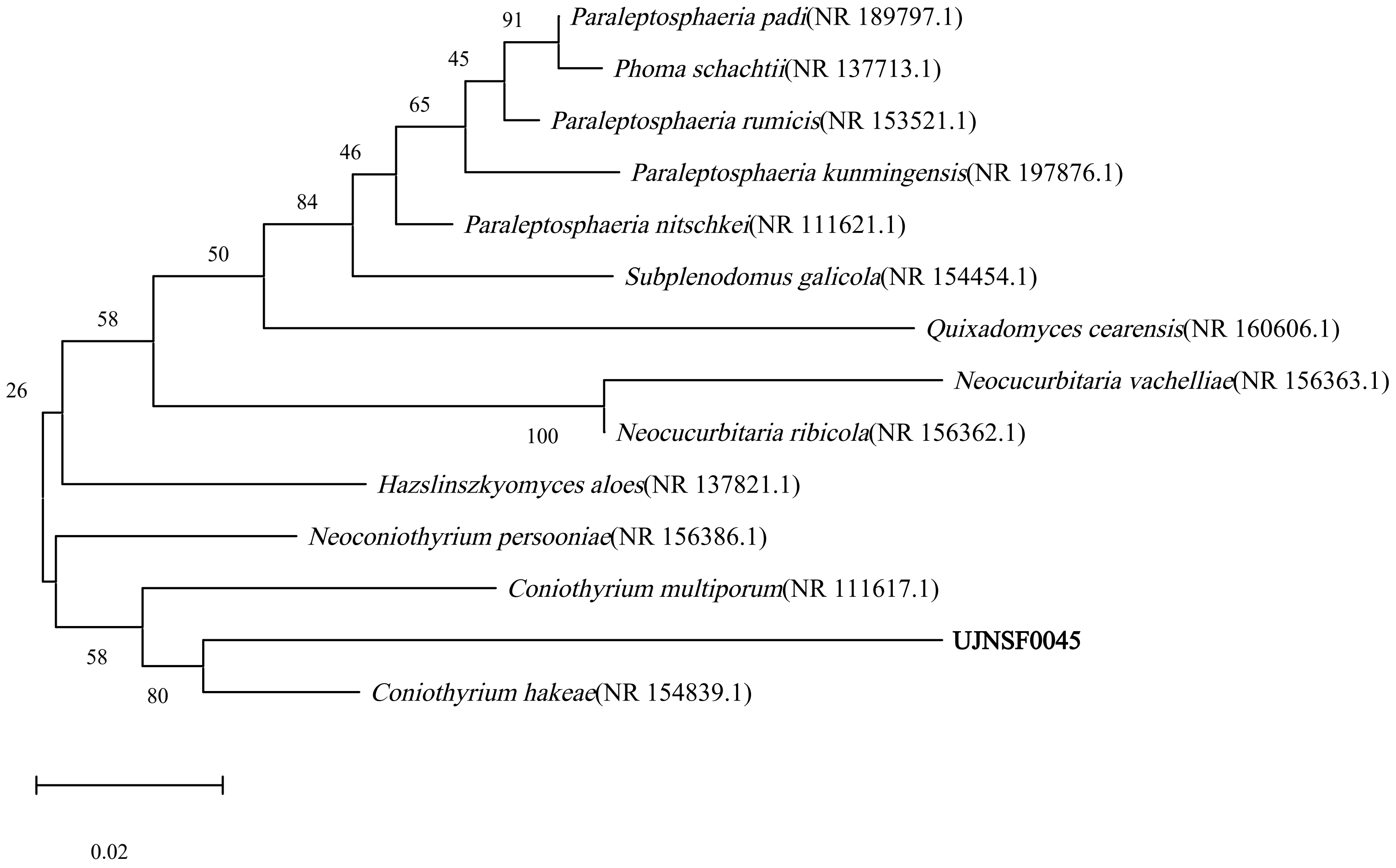
Paraleptosphaeria padi(NR 189797.1)
Phoma schachtii(NR 137713.1)
Paraleptosphaeria rumicis(NR 153521.1)
Paraleptosphaeria kunmingensis(NR 197876.1)
Paraleptosphaeria nitschkei(NR 111621.1)
Subplenodomus galicola(NR 154454.1)
Quixadomyces cearensis(NR 160606.1)
Neocucurbitaria vachelliae(NR 156363.1)
Neocucurbitaria ribicola(NR 156362.1)
Hazslinszkyomyces aloes(NR 137821.1)
Neoconiothyrium persooniae(NR 156386.1)
Coniothyrium multiporum(NR 111617.1)
UJNSF0045
Coniothyrium hakeae(NR 154839.1)
91
45
65
46
84
50
58
26
100
58
80
0.02

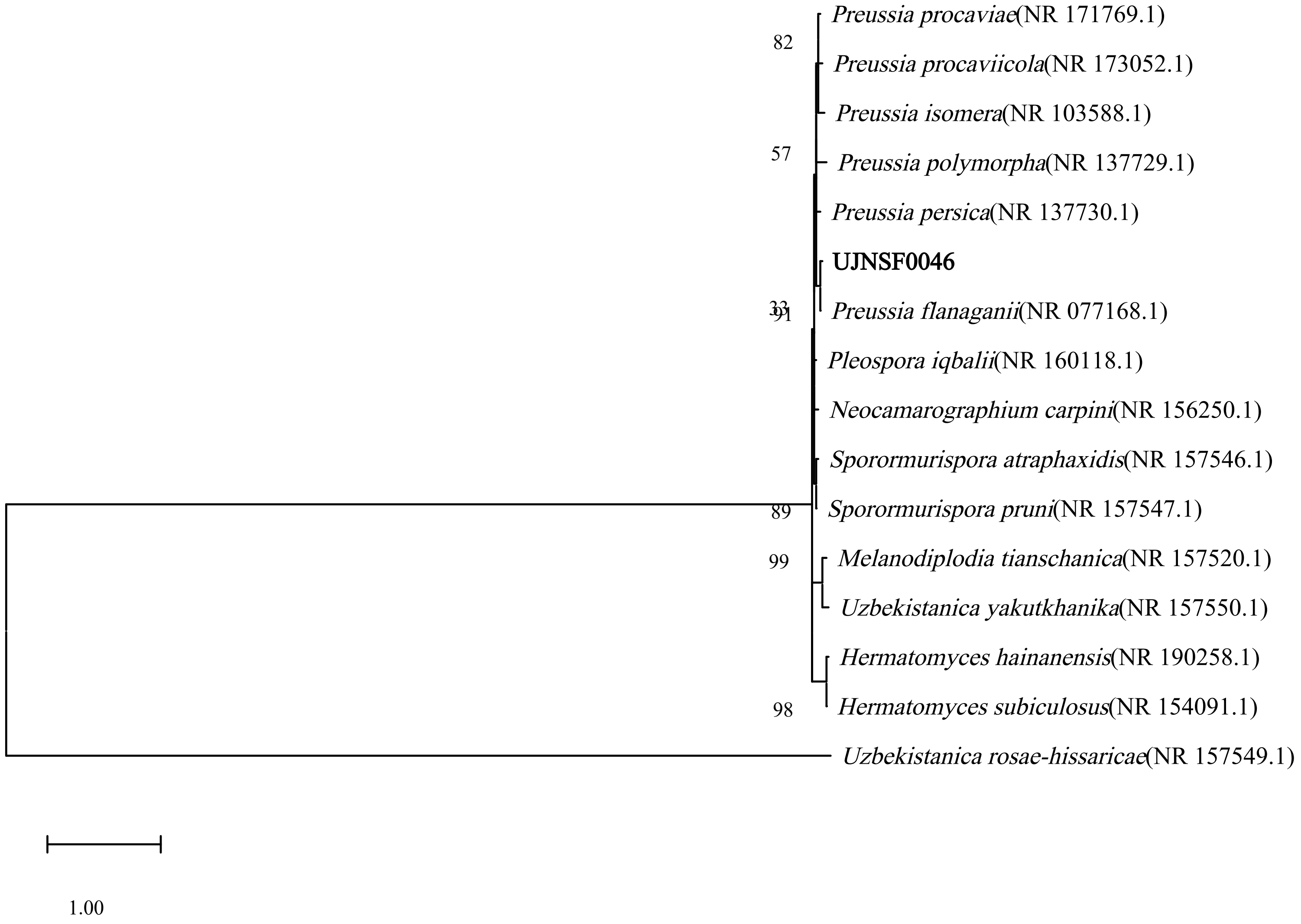
Preussia procaviae(NR 171769.1)
Preussia procaviicola(NR 173052.1)
Preussia isomera(NR 103588.1)
Preussia polymorpha(NR 137729.1)
Preussia persica(NR 137730.1)
UJNSF0046
Preussia flanaganii(NR 077168.1)
Pleospora iqbalii(NR 160118.1)
Neocamarographium carpini(NR 156250.1)
Sporormurispora atraphaxidis(NR 157546.1)
Sporormurispora pruni(NR 157547.1)
Melanodiplodia tianschanica(NR 157520.1)
Uzbekistanica yakutkhanika(NR 157550.1)
Hermatomyces hainanensis(NR 190258.1)
Hermatomyces subiculosus(NR 154091.1)
Uzbekistanica rosae-hissaricae(NR 157549.1)
82
57
33
91
89
99
98
1.00

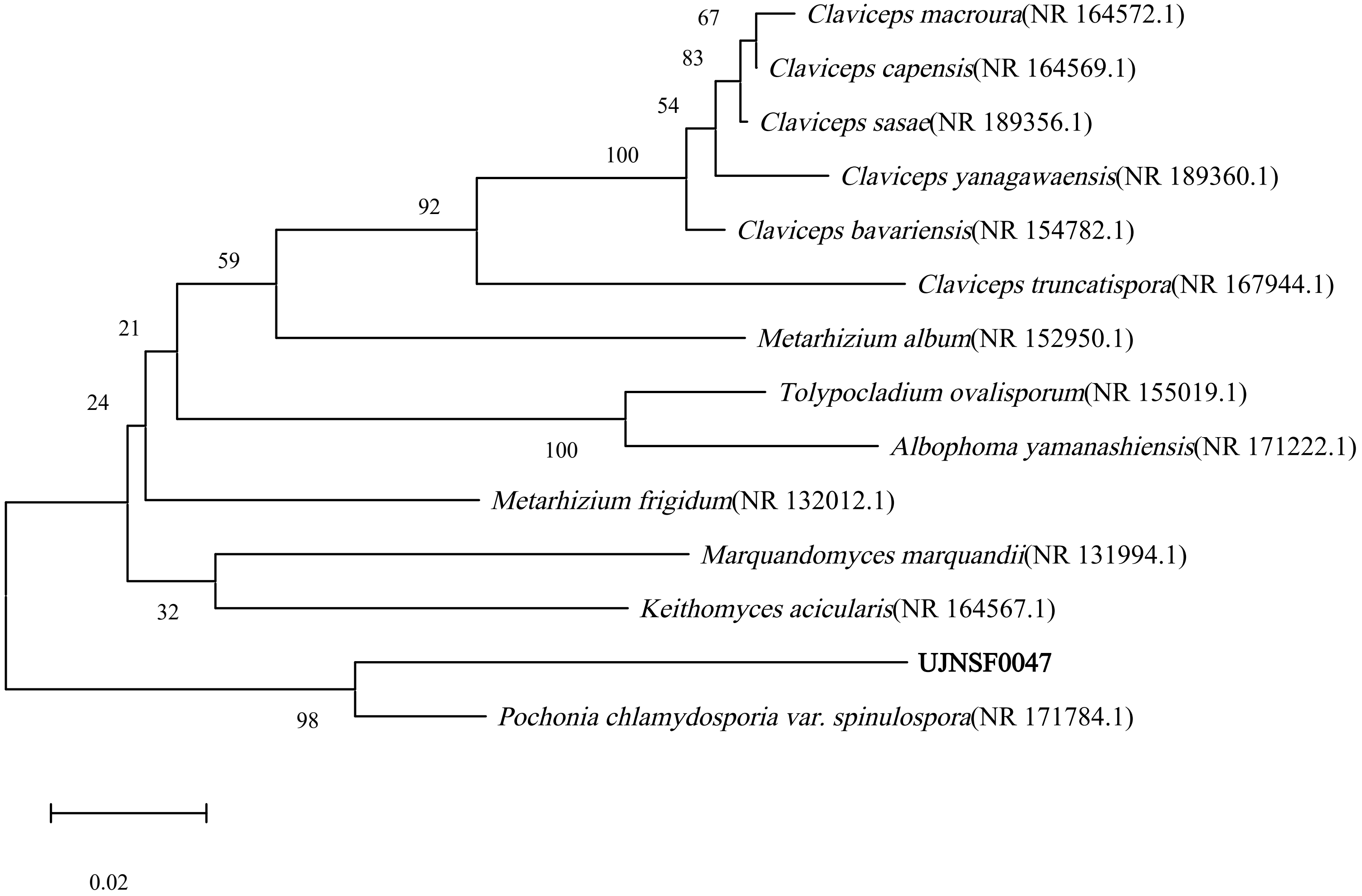

67
Claviceps macroura(NR 164572.1)
83
Claviceps capensis(NR 164569.1)
54
Claviceps sasae(NR 189356.1)
100
Claviceps yanagawaensis(NR 189360.1)
92
Claviceps bavariensis(NR 154782.1)
59
Claviceps truncatispora(NR 167944.1)
21
Metarhizium album(NR 152950.1)
24
Tolypocladium ovalisporum(NR 155019.1)
100
Albophoma yamanashiensis(NR 171222.1)
Metarhizium frigidum(NR 132012.1)
Marquandomyces marquandii(NR 131994.1)
32
Keithomyces acicularis(NR 164567.1)
UJNSF0047
98
Pochonia chlamydosporia var. spinulospora(NR 171784.1)
0.02

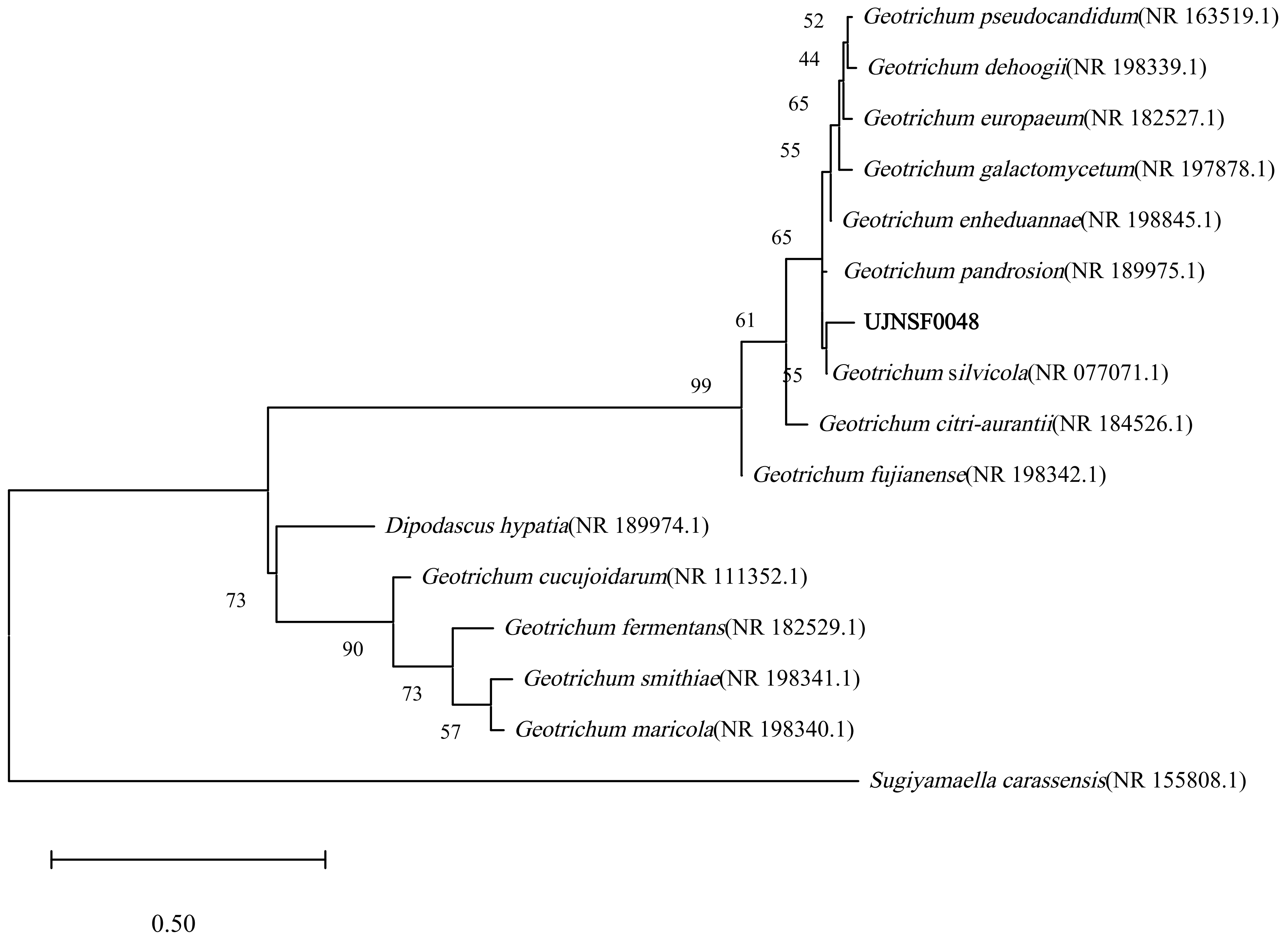

52
44
65
55
65
61
99
55
73
90
73
57
Geotrichum pseudocandidum(NR 163519.1)
Geotrichum dehoogii(NR 198339.1)
Geotrichum europaeum(NR 182527.1)
Geotrichum galactomycetum(NR 197878.1)
Geotrichum enheduannae(NR 198845.1)
Geotrichum pandrosion(NR 189975.1)
UJNSF0048
Geotrichum silvicola(NR 077071.1)
Geotrichum citri-aurantii(NR 184526.1)
Geotrichum fujianense(NR 198342.1)
Dipodascus hypatia(NR 189974.1)
Geotrichum cucujoidarum(NR 111352.1)
Geotrichum fermentans(NR 182529.1)
Geotrichum smithiae(NR 198341.1)
Geotrichum maricola(NR 198340.1)
Sugiyamaella carassensis(NR 155808.1)
0.50

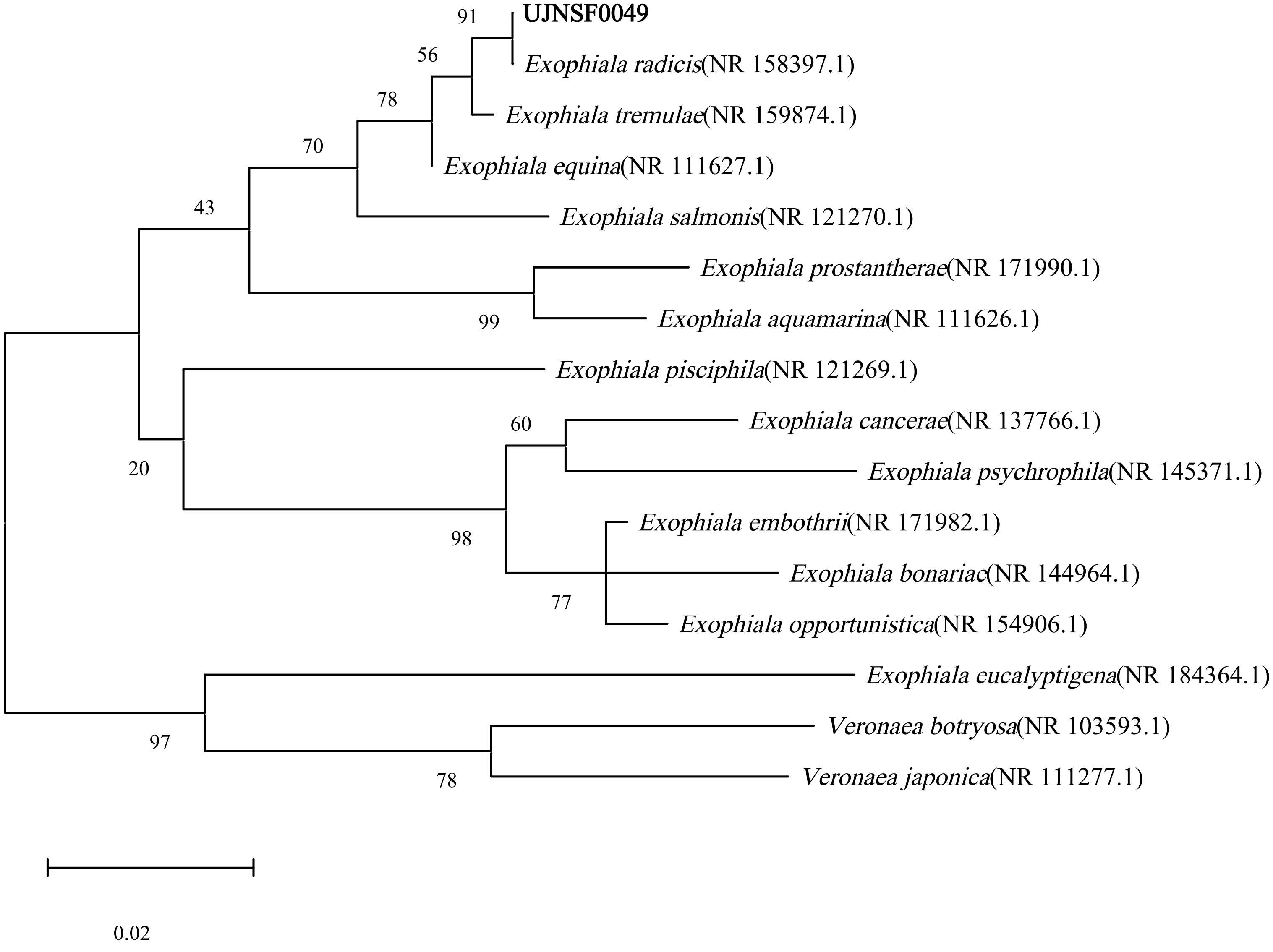
UJNSF0049
Exophiala radicis(NR 158397.1)
Exophiala tremulae(NR 159874.1)
Exophiala equina(NR 111627.1)
Exophiala salmonis(NR 121270.1)
Exophiala prostantherae(NR 171990.1)
Exophiala aquamarina(NR 111626.1)
Exophiala pisciphila(NR 121269.1)
Exophiala cancerae(NR 137766.1)
Exophiala psychrophila(NR 145371.1)
Exophiala embothrii(NR 171982.1)
Exophiala bonariae(NR 144964.1)
Exophiala opportunistica(NR 154906.1)
Exophiala eucalyptigena(NR 184364.1)
Veronaea botryosa(NR 103593.1)
Veronaea japonica(NR 111277.1)
91
56
78
70
43
99
20
60
98
77
97
78
0.02

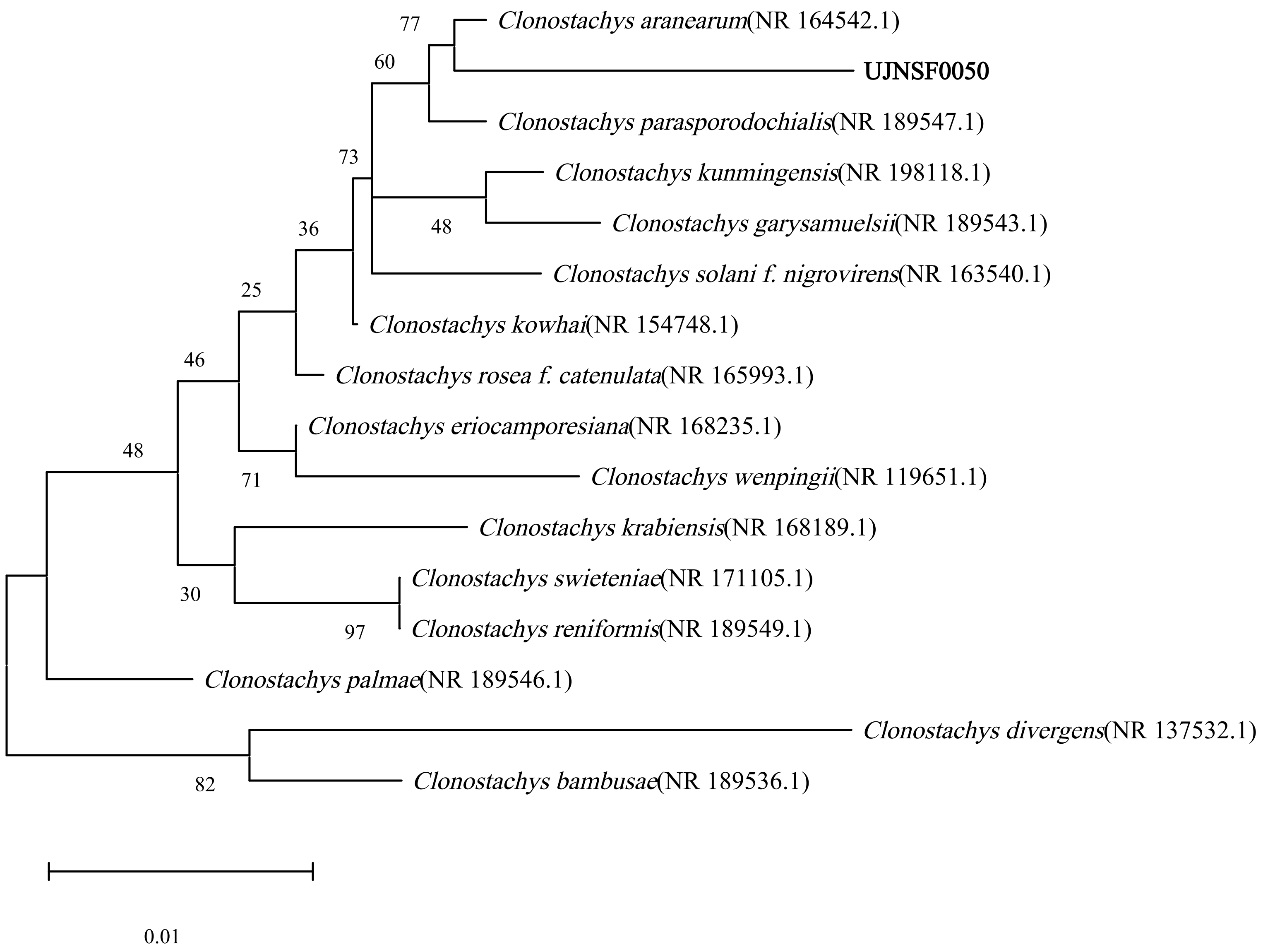
77
60
73
36
25
46
48
48
71
30
97
82
Clonostachys aranearum(NR 164542.1)
UJNSF0050
Clonostachys parasporodochialis(NR 189547.1)
Clonostachys kunmingensis(NR 198118.1)
Clonostachys garysamuelsii(NR 189543.1)
Clonostachys solani f. nigrovirens(NR 163540.1)
Clonostachys kowhai(NR 154748.1)
Clonostachys rosea f. catenulata(NR 165993.1)
Clonostachys eriocamporesiana(NR 168235.1)
Clonostachys wenpingii(NR 119651.1)
Clonostachys krabiensis(NR 168189.1)
Clonostachys swieteniae(NR 171105.1)
Clonostachys reniformis(NR 189549.1)
Clonostachys palmae(NR 189546.1)
Clonostachys divergens(NR 137532.1)
Clonostachys bambusae(NR 189536.1)
0.01

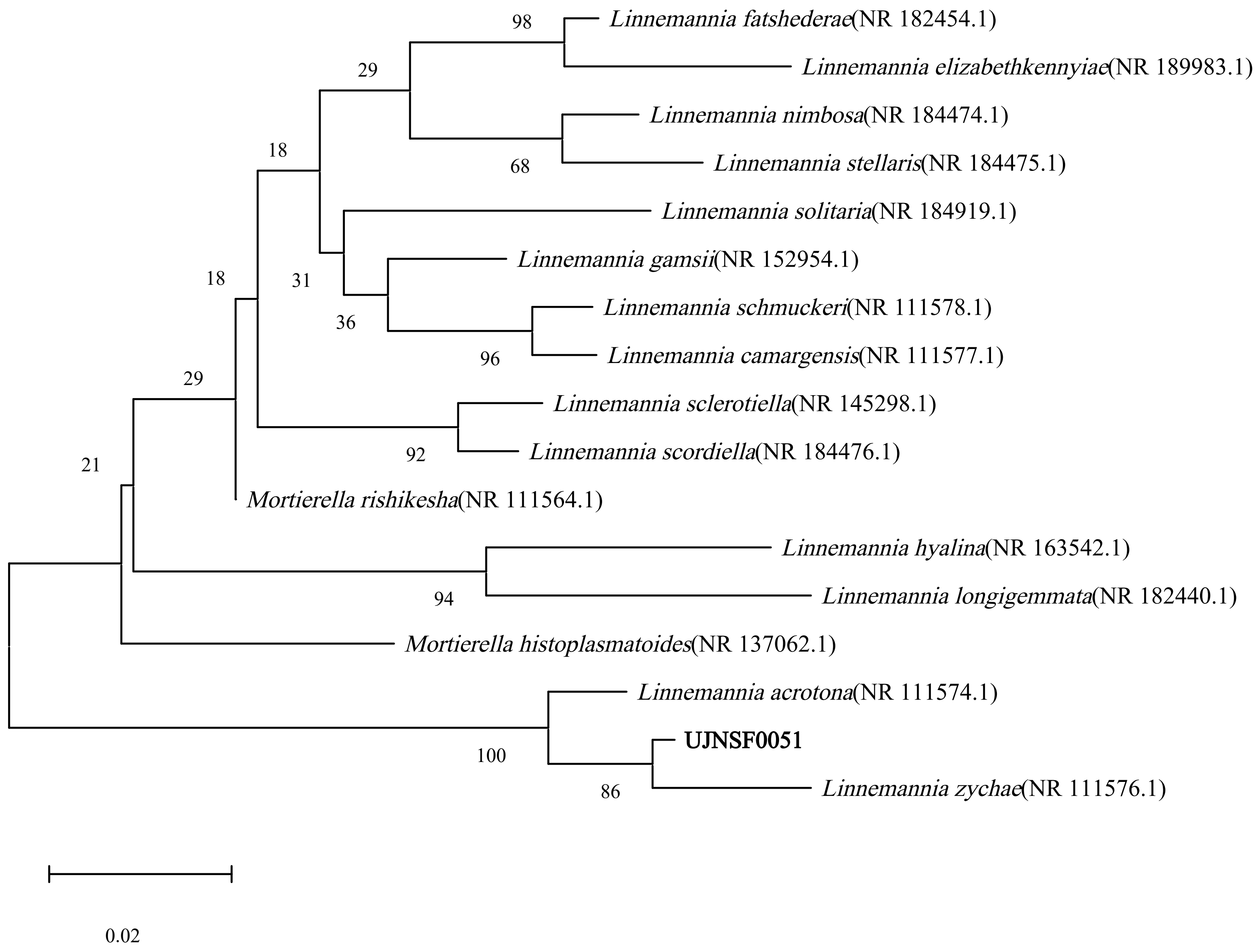
Linnemannia fatshederae(NR 182454.1)
Linnemannia elizabethkennyiae(NR 189983.1)
Linnemannia nimbosa(NR 184474.1)
Linnemannia stellaris(NR 184475.1)
Linnemannia solitaria(NR 184919.1)
Linnemannia gamsii(NR 152954.1)
Linnemannia schmuckeri(NR 111578.1)
Linnemannia camargensis(NR 111577.1)
Linnemannia sclerotiella(NR 145298.1)
Linnemannia scordiella(NR 184476.1)
Mortierella rishikesha(NR 111564.1)
Linnemannia hyalina(NR 163542.1)
Linnemannia longigemmata(NR 182440.1)
Mortierella histoplasmatoides(NR 137062.1)
Linnemannia acrotona(NR 111574.1)
UJNSF0051
Linnemannia zychae(NR 111576.1)
98
29
68
18
31
18
36
96
29
92
21
94
100
86
0.02

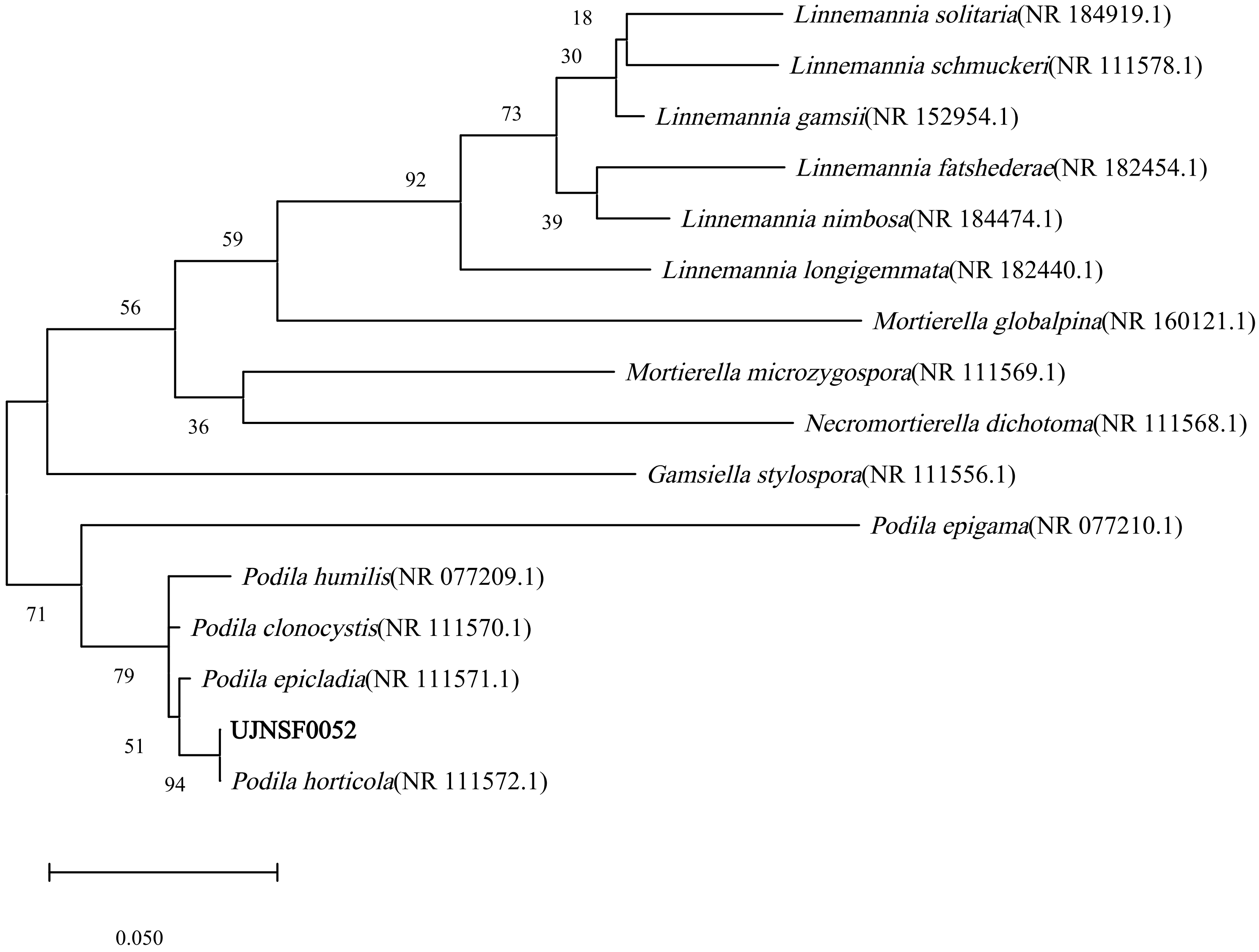
18
Linnemannia solitaria(NR 184919.1)
30
Linnemannia schmuckeri(NR 111578.1)
73
Linnemannia gamsii(NR 152954.1)
92
Linnemannia fatshederae(NR 182454.1)
39
Linnemannia nimbosa(NR 184474.1)
59
Linnemannia longigemmata(NR 182440.1)
56
Mortierella globalpina(NR 160121.1)
Mortierella microzygospora(NR 111569.1)
36
Necromortierella dichotoma(NR 111568.1)
Gamsiella stylospora(NR 111556.1)
Podila epigama(NR 077210.1)
Podila humilis(NR 077209.1)
71
Podila clonocystis(NR 111570.1)
79
Podila epicladia(NR 111571.1)
51
UJNSF0052
94
Podila horticola(NR 111572.1)
0.050

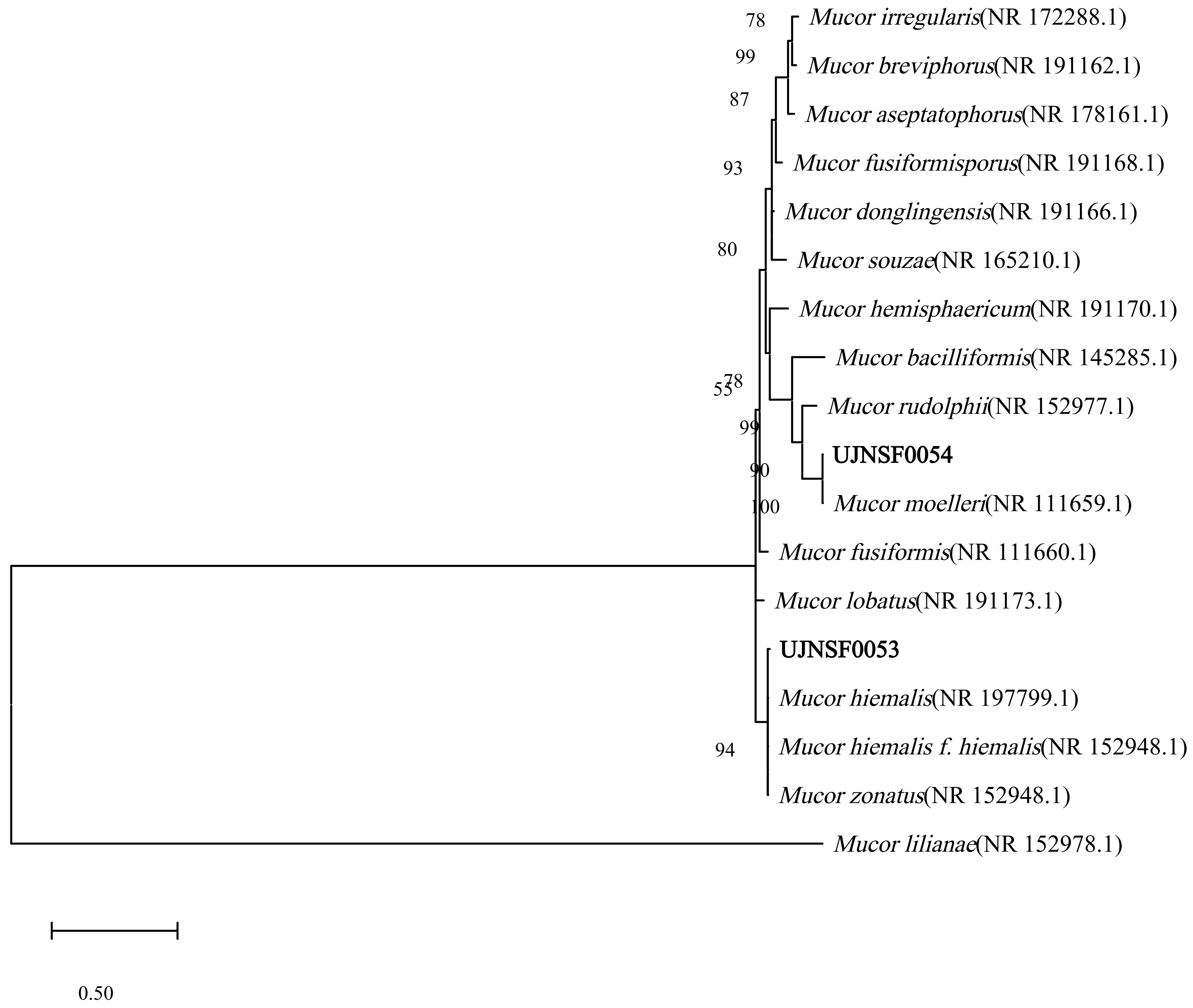
78
Mucor irregularis(NR 172288.1)
99
Mucor breviphorus(NR 191162.1)
87
Mucor aseptatophorus(NR 178161.1)
93
Mucor fusiformisporus(NR 191168.1)
Mucor donglingensis(NR 191166.1)
80
Mucor souzae(NR 165210.1)
Mucor hemisphaericum(NR 191170.1)
Mucor bacilliformis(NR 145285.1)
55
78
Mucor rudolphii(NR 152977.1)
99
UJNSF0054
90
Mucor moelleri(NR 111659.1)
100
Mucor fusiformis(NR 111660.1)
Mucor lobatus(NR 191173.1)
UJNSF0053
Mucor hiemalis(NR 197799.1)
94
Mucor hiemalis f. hiemalis(NR 152948.1)
Mucor zonatus(NR 152948.1)
Mucor lilianae(NR 152978.1)
0.50

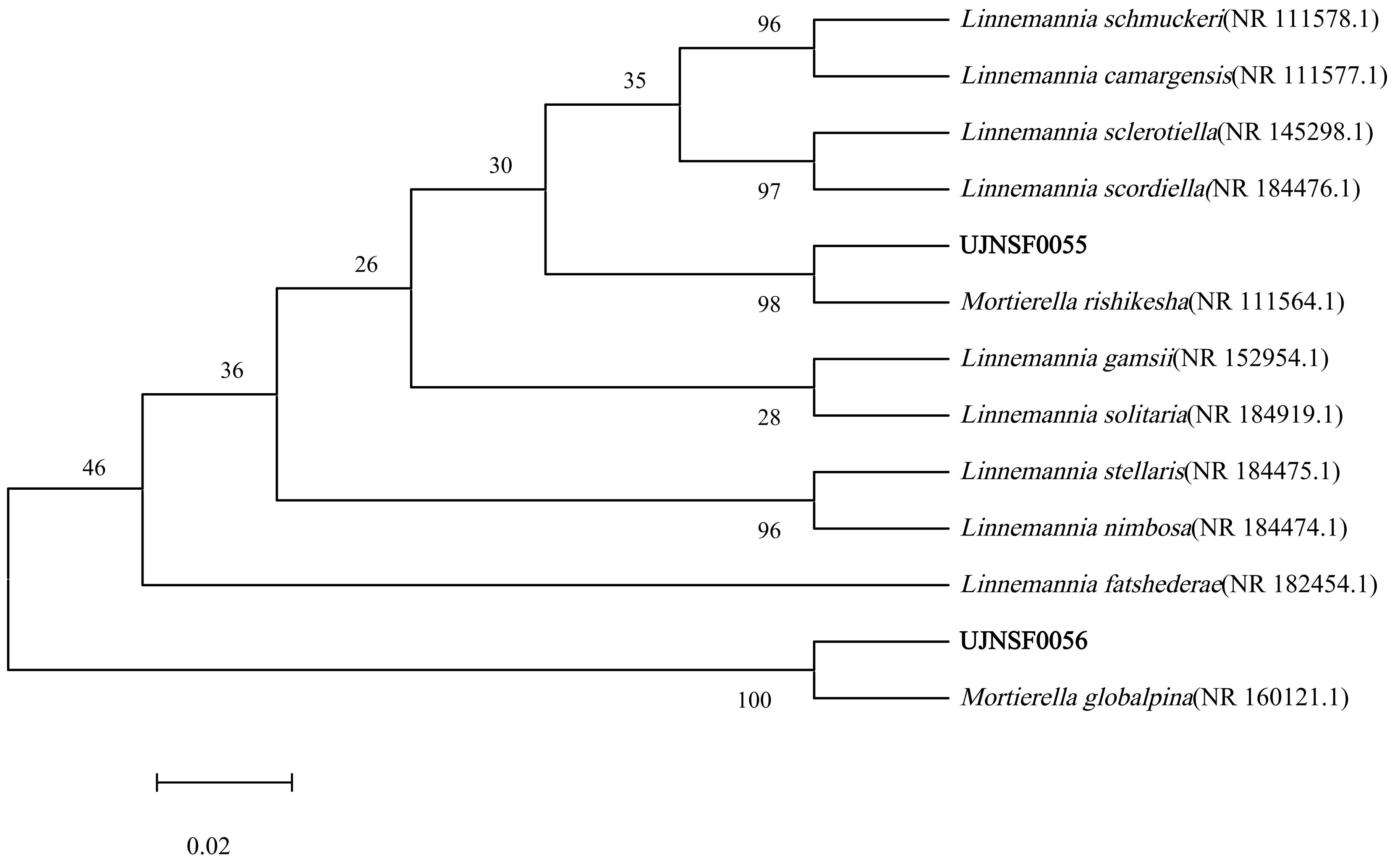
96
35
30
26
36
46
97
98
28
96
100
Linnemannia schmuckeri(NR 111578.1)
Linnemannia camargensis(NR 111577.1)
Linnemannia sclerotiella(NR 145298.1)
*Linnemannia scordiella(*NR 184476.1)
UJNSF0055
Mortierella rishikesha(NR 111564.1)
Linnemannia gamsii(NR 152954.1)
Linnemannia solitaria(NR 184919.1)
Linnemannia stellaris(NR 184475.1)
Linnemannia nimbosa(NR 184474.1)
Linnemannia fatshederae(NR 182454.1)
UJNSF0056
Mortierella globalpina(NR 160121.1)
0.02

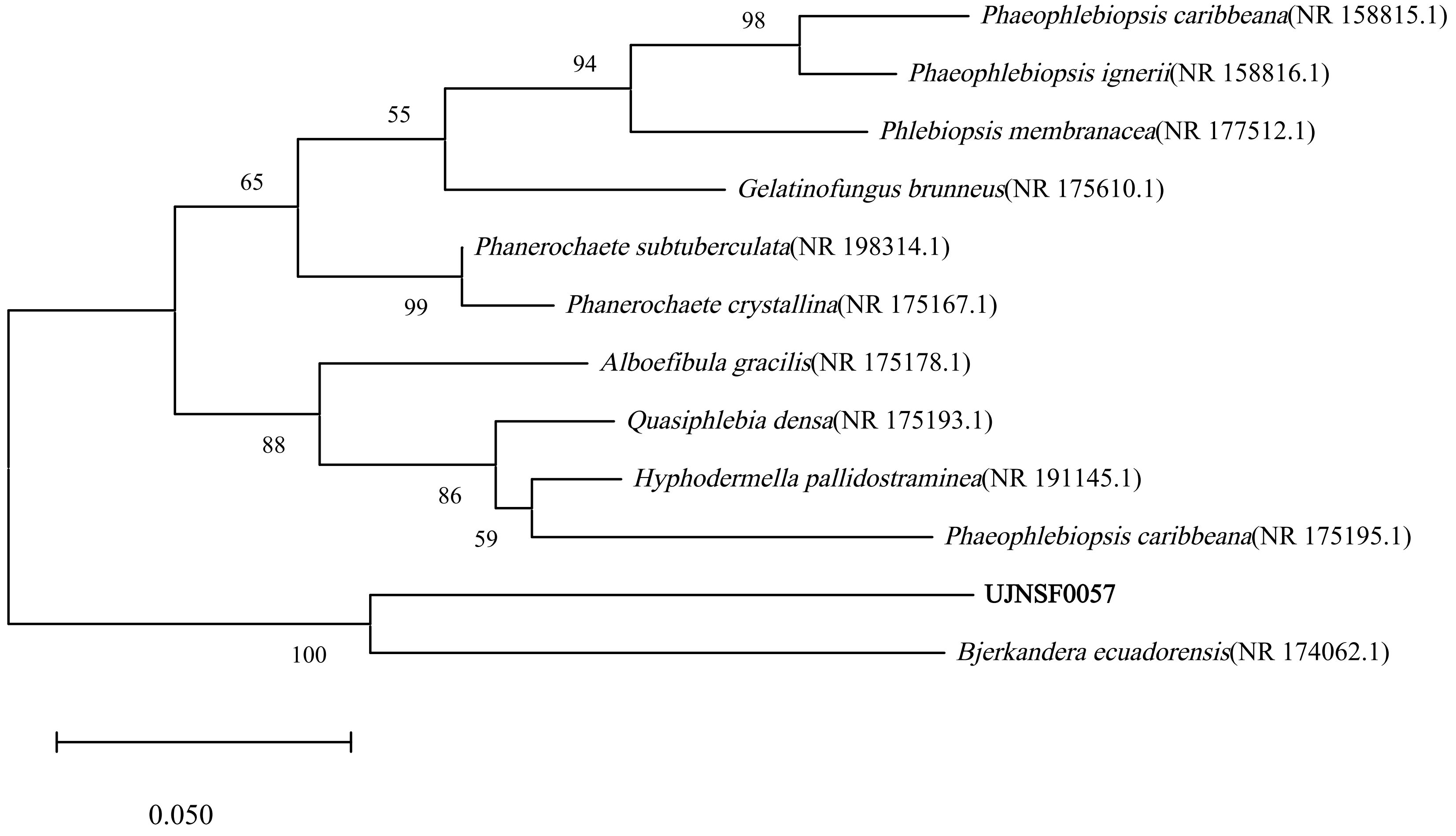
Phaeophlebiopsis caribbeana(NR 158815.1)
Phaeophlebiopsis ignerii(NR 158816.1)
Phlebiopsis membranacea(NR 177512.1)
Gelatinofungus brunneus(NR 175610.1)
Phanerochaete subtuberculata(NR 198314.1)
Phanerochaete crystallina(NR 175167.1)
Alboefibula gracilis(NR 175178.1)
Quasiphlebia densa(NR 175193.1)
Hyphodermella pallidostraminea(NR 191145.1)
Phaeophlebiopsis caribbeana(NR 175195.1)
UJNSF0057
Bjerkandera ecuadorensis(NR 174062.1)
98
94
55
65
99
88
86
59
100
0.050

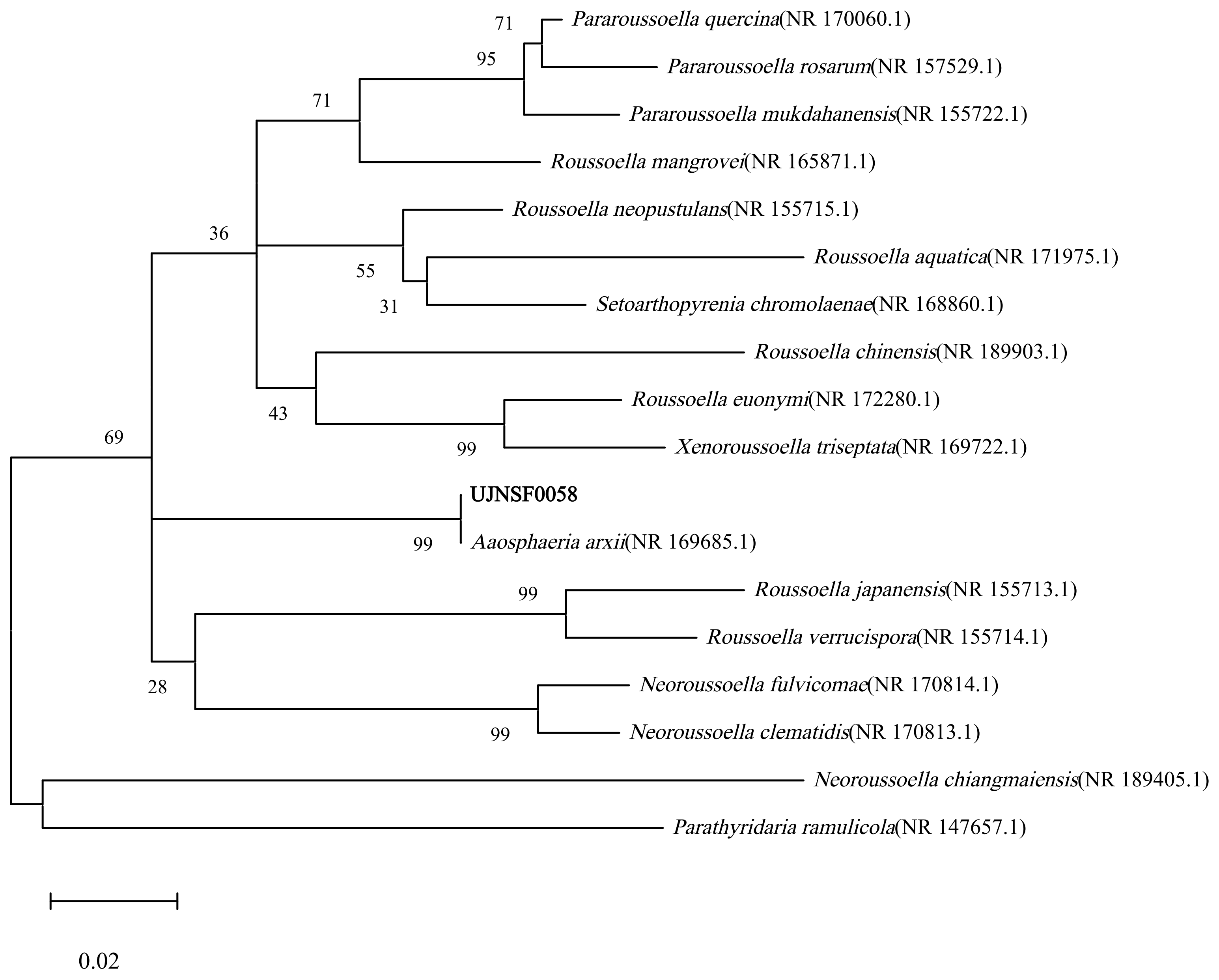
Pararoussoella quercina(NR 170060.1)
Pararoussoella rosarum(NR 157529.1)
Pararoussoella mukdahanensis(NR 155722.1)
Roussoella mangrovei(NR 165871.1)
Roussoella neopustulans(NR 155715.1)
Roussoella aquatica(NR 171975.1)
Setoarthopyrenia chromolaenae(NR 168860.1)
Roussoella chinensis(NR 189903.1)
Roussoella euonymi(NR 172280.1)
Xenoroussoella triseptata(NR 169722.1)
UJNSF0058
Aaosphaeria arxii(NR 169685.1)
Roussoella japanensis(NR 155713.1)
Roussoella verrucispora(NR 155714.1)
Neoroussoella fulvicomae(NR 170814.1)
Neoroussoella clematidis(NR 170813.1)
Neoroussoella chiangmaiensis(NR 189405.1)
Parathyridaria ramulicola(NR 147657.1)
71
95
71
36
55
31
43
99
69
99
99
28
99
0.02

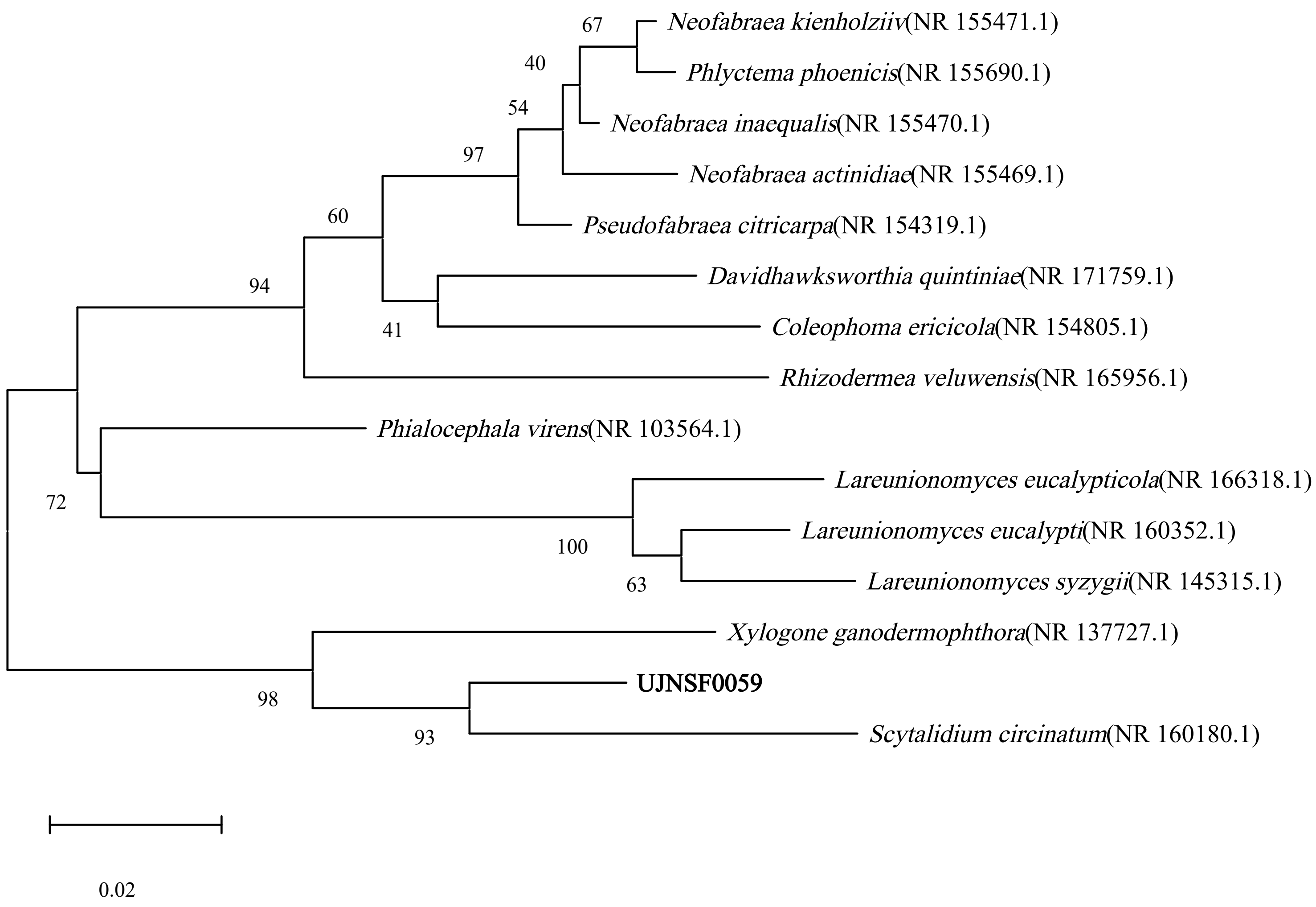
Neofabraea kienholziiv(NR 155471.1)
Phlyctema phoenicis(NR 155690.1)
Neofabraea inaequalis(NR 155470.1)
Neofabraea actinidiae(NR 155469.1)
Pseudofabraea citricarpa(NR 154319.1)
Davidhawksworthia quintiniae(NR 171759.1)
Coleophoma ericicola(NR 154805.1)
Rhizodermea veluwensis(NR 165956.1)
Phialocephala virens(NR 103564.1)
Lareunionomyces eucalypticola(NR 166318.1)
Lareunionomyces eucalypti(NR 160352.1)
Lareunionomyces syzygii(NR 145315.1)
Xylogone ganodermophthora(NR 137727.1)
UJNSF0059
Scytalidium circinatum(NR 160180.1)
67
40
54
97
60
94
41
72
100
63
98
93
0.02

3. The HPLC analysis of the 24 crude extracts derived from the six strains with well antibacterial activities. (0-35 min: 5% - 100% CH_3OH-H_2O; 35-45 min 100% CH_3OH; flow rate at 1.0 mL/min and detection at 210, 230, 254 and 320 nm)

UJNSF0003-1# medium

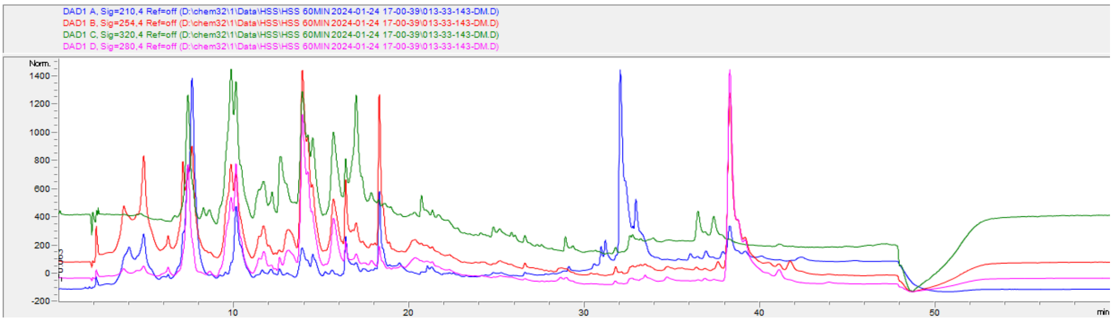

UJNSF0003-2# medium

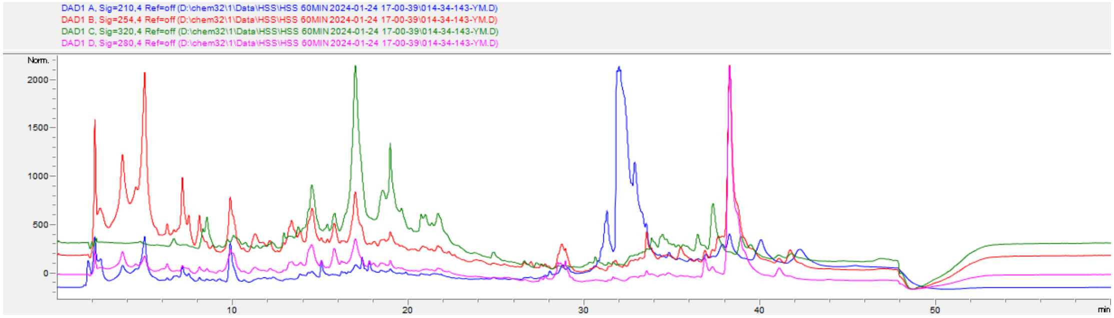

UJNSF0003-3# medium

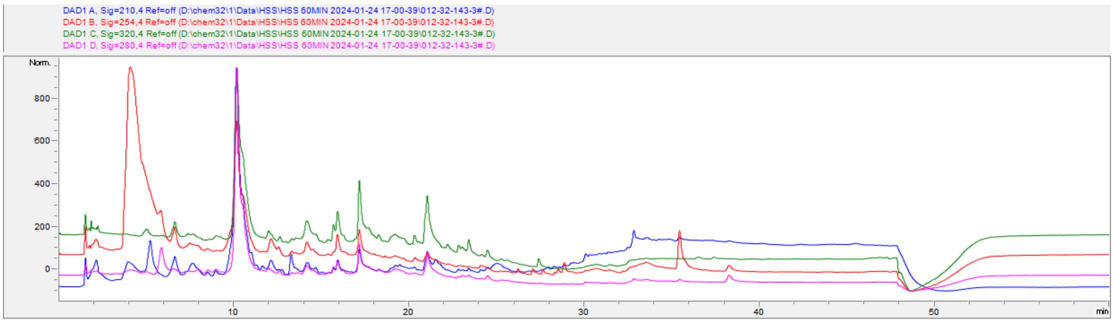

UJNSF0003-4# medium

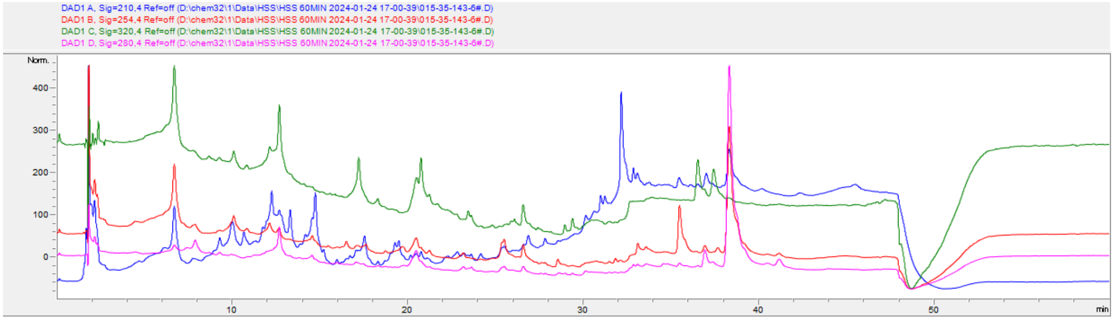

UJNSF0014-1# medium

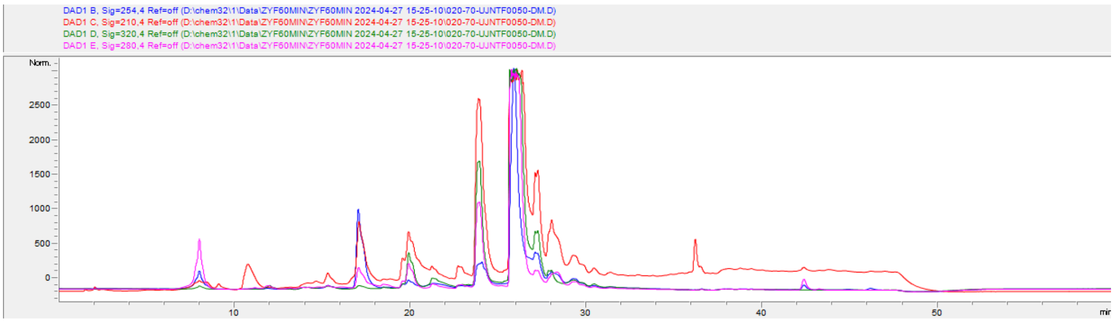

UJNSF0014-2# medium

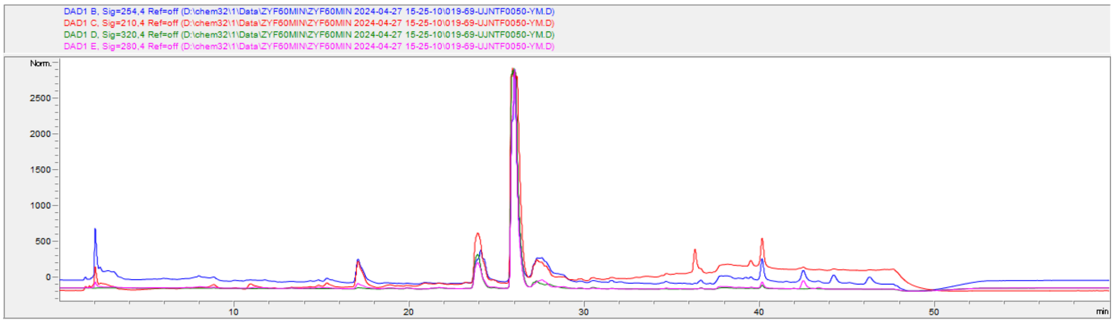

UJNSF0014-3# medium

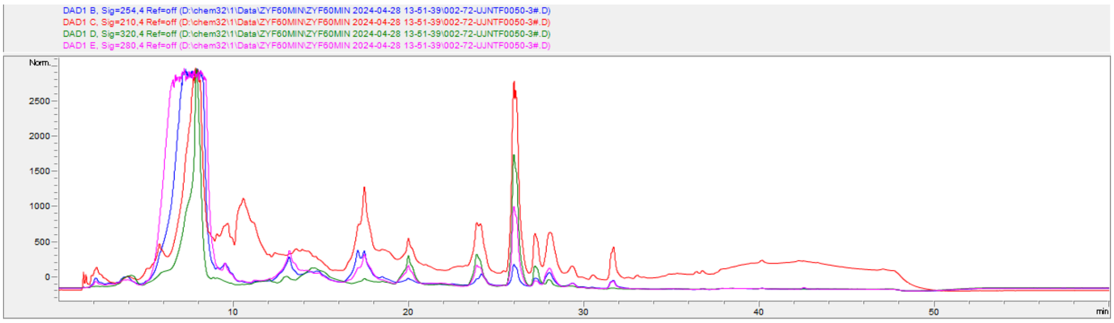

UJNSF0014-4# medium

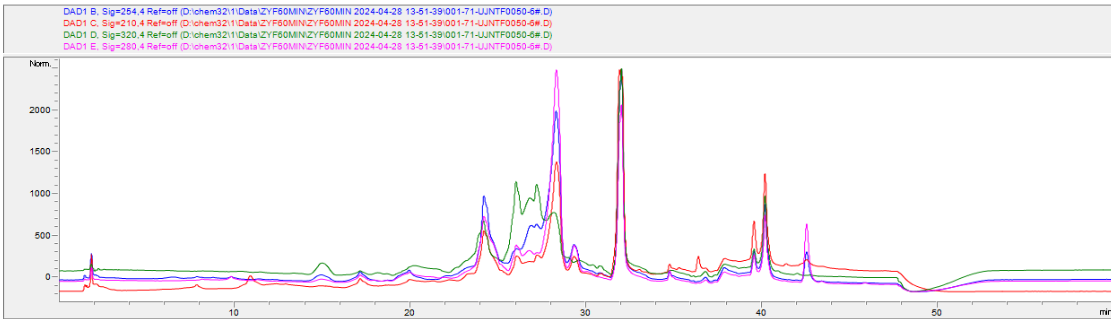

UJNSF0017-1# medium

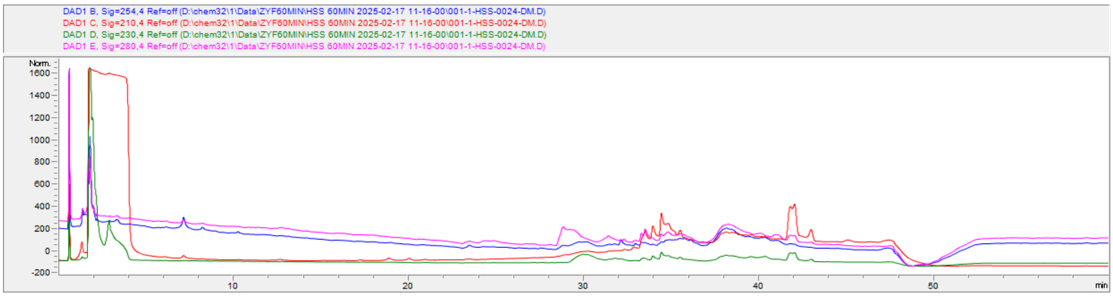

UJNSF0017-2# medium

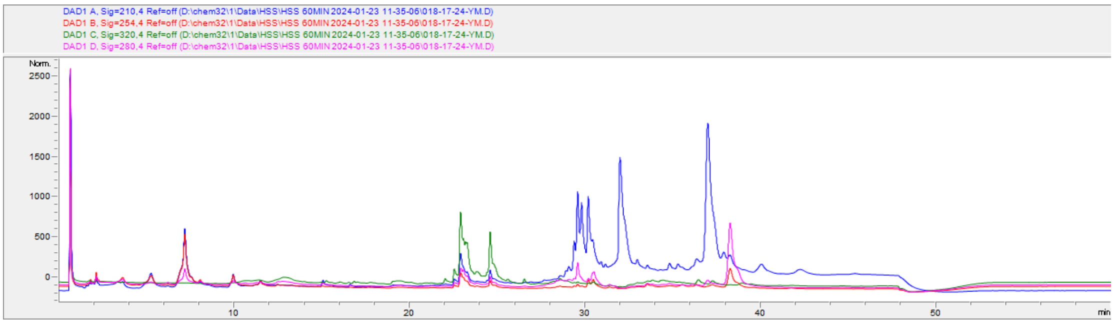

UJNSF0017-3# medium

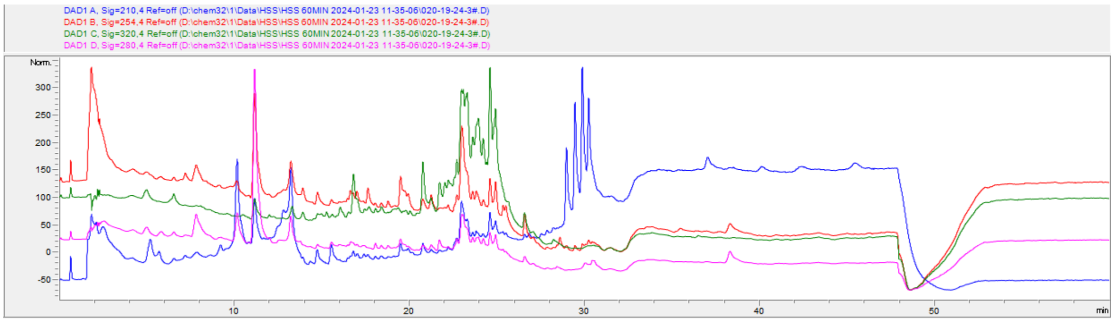

UJNSF0017-4# medium

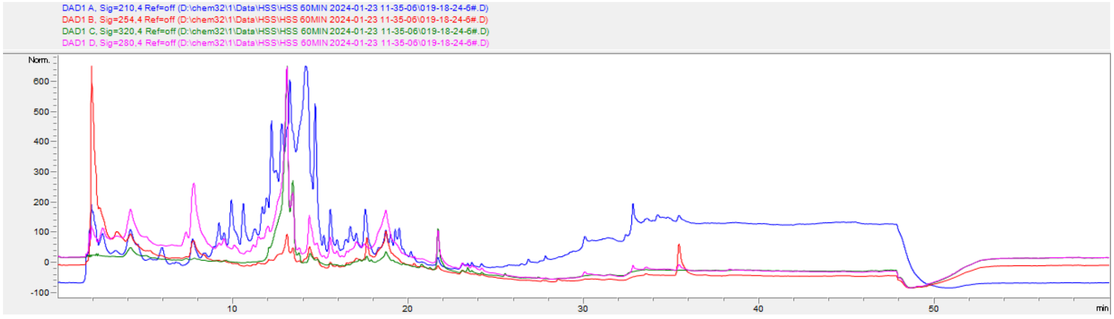

UJNSF0026-1# medium

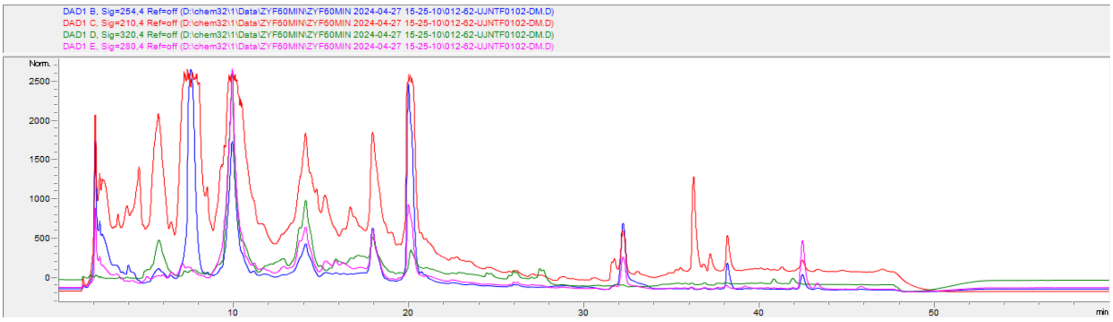

UJNSF0026-2# medium

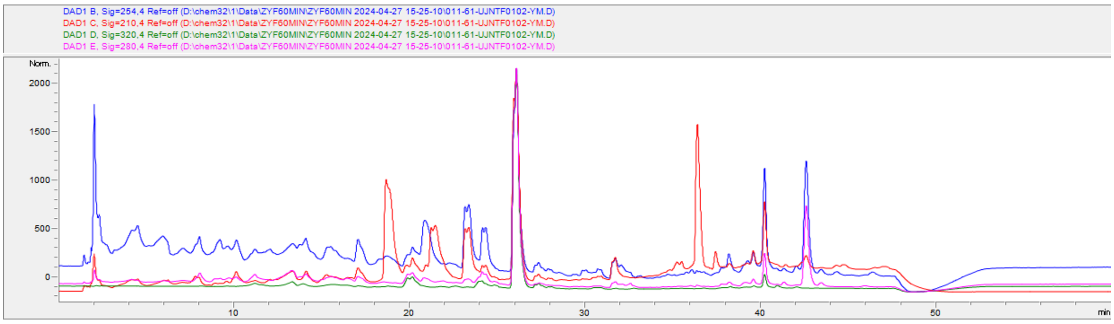

UJNSF0026-3# medium

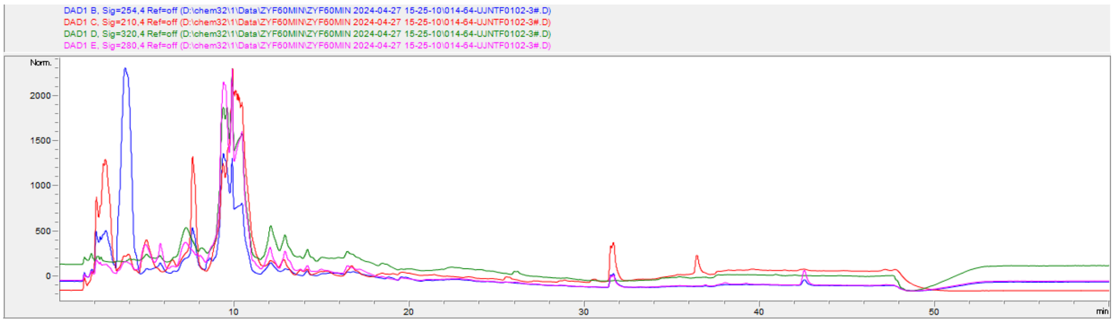

UJNSF0026-4# medium

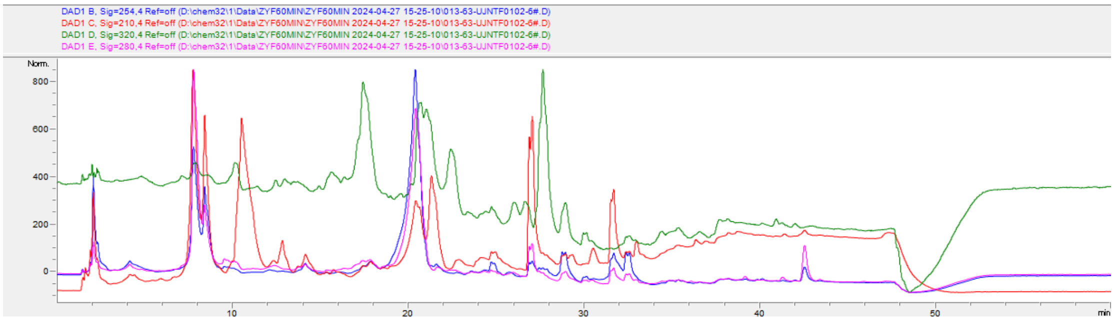

UJNSF0035-1# medium

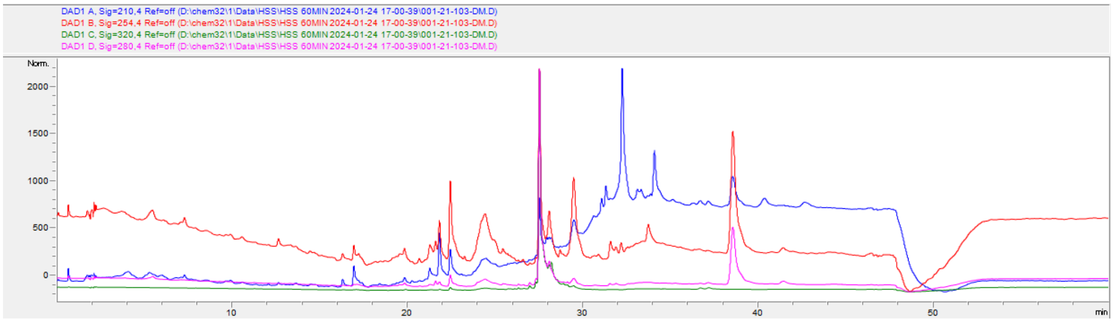

UJNSF0035-2# medium

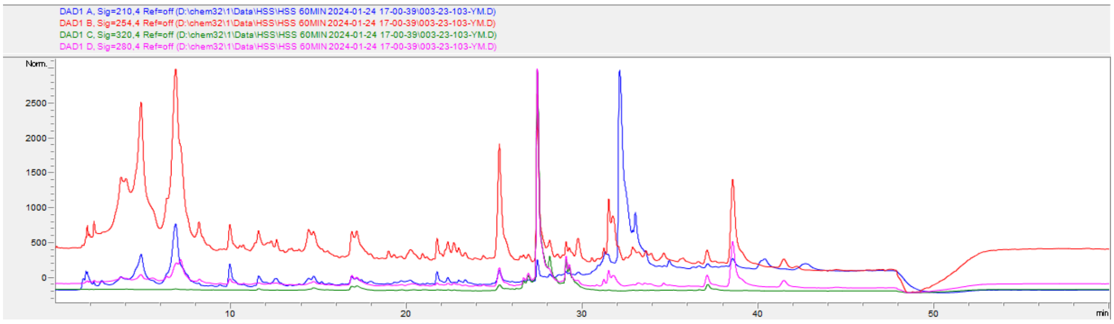

UJNSF0035-3# medium

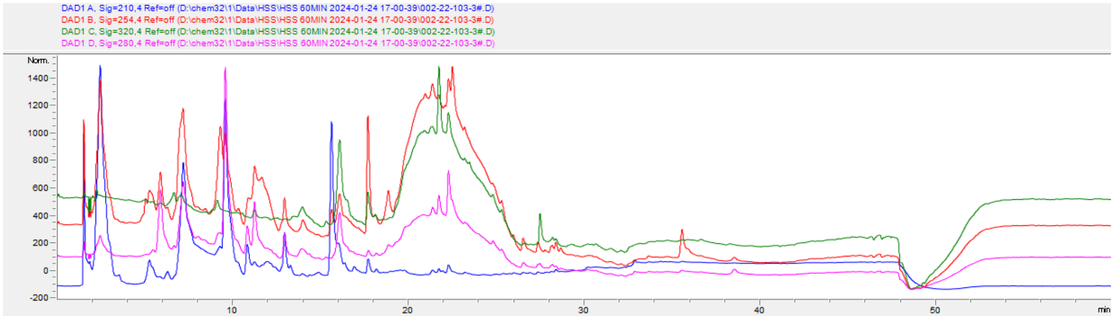

UJNSF0035-4# medium

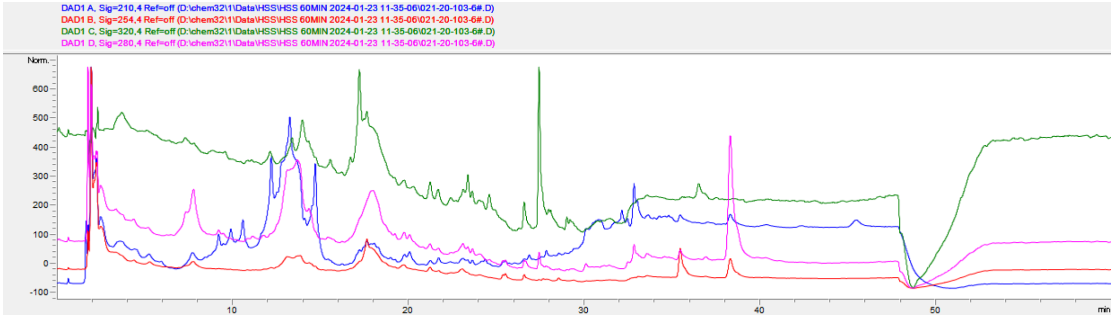

UJNSF0039-1# medium

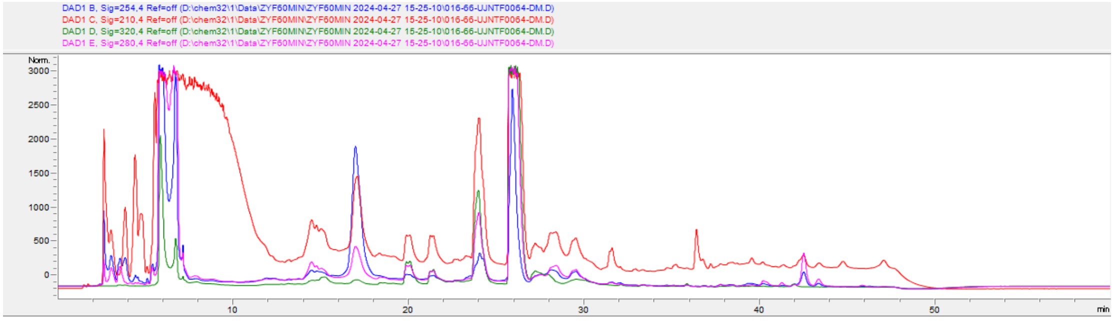

UJNSF0039-2# medium

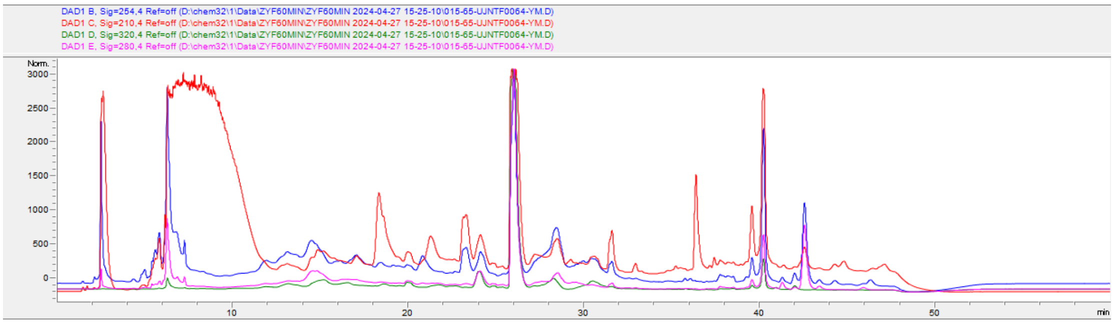

UJNSF0039-3# medium

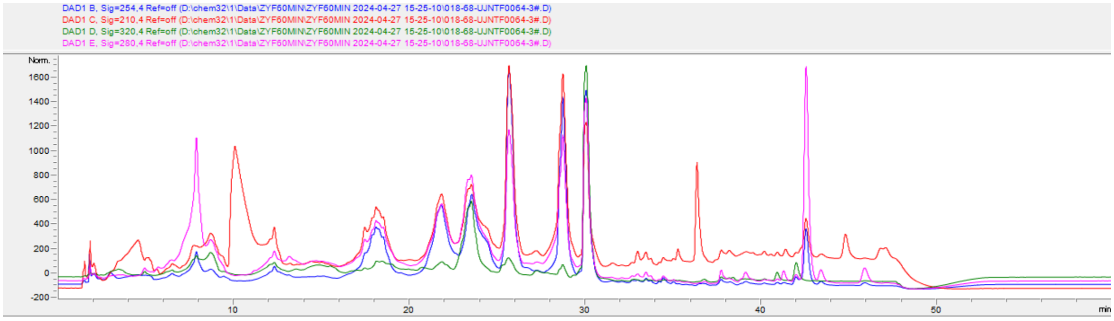

UJNSF0039-4# medium

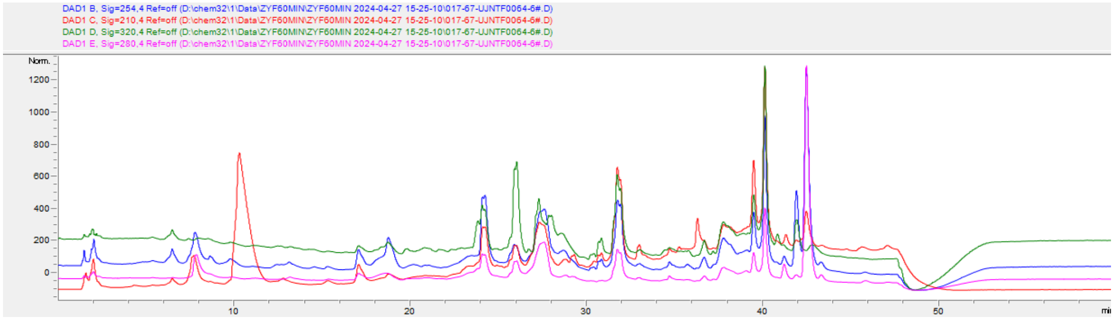

Supplement: Supplementary file 1 [file jof-11-00276-s001.zip › jof-3517347-supplementary.pdf]
